# Data, metrics, and methods for arthropod and fungal herbivory at the dawn of angiosperm diversification: The Rose Creek plant assemblage of Nebraska, U.S.A.

**DOI:** 10.1016/j.dib.2022.108170

**Published:** 2022-04-14

**Authors:** Lifang Xiao, Conrad C. Labandeira, David L. Dilcher, Dong Ren

**Affiliations:** a College of Life Sciences and Academy for Multidisciplinary Studies, Capital Normal University, Beijing 100048, China; b Department of Paleobiology, National Museum of Natural History, Smithsonian Institution, Washington, DC 20013, USA; c Department of Entomology, University of Maryland, College Park, MD 20742, USA; d Department of Biology, Indiana University, Bloomington, IN 47405, USA

**Keywords:** Cretaceous angiosperm flora, Herbivory richness and intensity, Host-plant specialization, Feeding event occurrences, Rarefaction analyses, Component communities, Non-metric multidimensional scaling, Functional feeding group stoichiometry

## Abstract

The data presented in this article are related to the research article titled “Arthropod and fungal herbivory at the dawn of angiosperm diversification: The Rose Creek plant assemblage of Nebraska, U.S.A.” (Xiao et al., 2021). These data correspond to an examination of arthropod and fungal herbivory on 2084 plant specimens from the Early Cretaceous (late Albian) Rose Creek locality of southeastern Nebraska, USA. Ten datasets have been assembled to describe and contextualize the diversity and intensity of herbivory at Rose Creek, as documented in Appendices of the online supplementary material. Appendices S4 and S5 provide a list and the frequency distributions by major clade and species/morphotype of all plant taxa examined. Appendix S6 outlines general procedures for documenting herbivory on plants and how the data was acquired. Appendix S9a and S9b provide rarefaction analyses for plant taxa to demonstrate sampling sufficiency, which is paralleled by rarefaction analyses of Appendix S9c and S9d that indicate sampling of damage types are robust. The comprehensive dataset of Appendix S12 lists plant taxa horizontally by major clade/group and species/morphotype versus vertically listed feeding classes, functional feeding groups (FFGs) and damage types (DTs). The basic metrics of DTs, feeding event occurrences, DT host-plant specialization, and number of matrix cells are displayed, with data subtotals and totals. This data matrix serves as the central source of data for the study, and records the six metrics of DT richness, DT frequency, DT host-plant specialization, percent of area herbivorized, and feeding event occurrences. Three of these metrics are used for establishing component community structure of the three most herbivorized taxa (Figs 8–10), and the relationships among plant hosts and FFGs in the non-metric multidimensional scaling analysis (Fig. 11) (Xiao et al., 2021). Appendix S15 is a list DTs, with their assigned host-plant specialization of 1 for generalized, 2 for intermediate specificity, and 3 for specialized. Appendix S16 is a table that provides plant surface areas (cm^2^) and their percentages that have been removed due to herbivory. Appendix S18 provides descriptions and ancillary data for 14 new DTs described from Rose Creek. A listing of the herbivory index (herbivorized surface area divided by total surface area) of plant assemblages and individual plant species in Appendix S19 provides comparisons among Rose Creek, other fossil, and modern plant assemblages. Lastly, Appendix S23 lists from the literature of arthropod species forming the well-documented herbivore component communities of five modern plant species to the three most herbivorized taxa at Rose Creek shown in Fig. 12. Some of the metrics used to quantitatively measure the diversity and intensity of herbivory are recent, such as feeding event occurrences, whereas others such as herbivorized surface area and host-plant specialization values have had a longer use in plant–arthropod studies.

## Specifications Table

**Table:** 

Subject	Ecology
Specific subject area	Early Cretaceous arthropod and pathogen herbivory on early angiosperms.
	The data consists of two complimentary approaches toward assessing arthropod herbivory. First, a descriptive analysis categorizes the affinities and diversity of the plant taxa and describes the spectrum of functional feeding groups (FFGs) and damage types (DTs) on leaves and other organs of the plant groups. Second, quantitative metrics for the entire plant assemblage details the two basic approaches of herbivory richness and herbivory intensity. Herbivory richness is evaluated by the three metrics of DT richness, component community structure (a composite measure), and the degree of herbivory specialization. By contrast, herbivory intensity is determined by the three metrics of DT frequency, herbivorized surface area, and feeding event occurrences.
Type of data	Photographic images; data matrices; univariate, bivariate, and multivariate plots; and tables.
How data were acquired	For herbivore damage on plant specimens, data capture was done visually with assistance of relevant plant literature for Cretaceous plants [2], sources for DT determinations [3], [4], [5], and experience of the coauthors. Light microscopy was used for examination and photographic documentation. Photography was done with a Canon 50D camera and a Canon EF-S60 mm f/2.8 macro lens, Nikon SMZ 25 microscope with a Nikon DS-Ri 2 digital camera system, Digital imaging and processing was based on *Image J* capture of specimen data, and Adobe® Illustrator, Drawer, and Photoshop software. Data analyses were implemented by R and Excel.
Data format	Raw.
Parameters for data collection	Plant fossils were previously prepared for removal of overlying sedimentary matrix. Photography was done with a combination of overhead flood lighting and low-angle spot lighting, respectively, to provide color balance and accentuate plant and damage detail. For the herbivorized surface area study, camera filters were used to emphasize details of the insect damage, differentiation of leaf margin versus rock matrix, and damaged versus undamaged portions of plants.
Description of data collection	All recognizable plant specimens (leaves, pinnules, axes, roots, reproductive material) greater in area than about 0.25 cm^2^ were identified. When possible, herbivory data were collected, including DT richness, component community structure for the three most herbivorized taxa, DT plant-host specialization values, DT frequency, total and herbivorized surface area, and feeding event occurrences. All data were entered into excel data files. Comparisons were made between the herbivorized surface area (herbivory index) of the Rose Creek plant assemblage and its constituent major clades and species/morphotypes versus other analogous taxa and plant assemblages from the fossil and modern records. The stoichiometry of the major functional feeding groups – a composite ectophytic feeding, piercing and sucking, mining, and galling – was compared for the three most herbivorized Rose Creek taxa with five modern, well documented taxa. Relevant sources [1], [2], [3], [4], [5] informed many of these decisions.
Data source location	Rose Creek locality: South of the city of Fairbury, in Jefferson County, Nebraska, USA. The topographic map coordinates are NW1/4, SE 1/4, Sec.14, T 1 N, R2E. The geographic coordinates are 40°03.01′N, 97°10.12′W) [6]. Institutional repository: Florida Museum of Natural History, University of Florida, 1659 Museum Road, Gainesville, Florida, 32,611–7800, USA.
Data accessibility	Within the article [1] and online supplementary data linked to this article at http://dx.doi.org/10.17632/9rxg3tc8s2.2.
Related research article	L. Xiao, C.C. Labandeira, D.L. Dilcher, and D. Ren. Arthropod and fungal herbivory at the dawn of angiosperm diversification: The Rose Creek plant assemblage of Nebraska, U.S.A. Cret. Res. (2021), (https://doi.org/10.1016/j.cretres.2021.105088) [1].

## Value of the Data

• This study uses highly sampled plant-host data and arthropod and pathogen herbivory data from one of the diverse, abundant, and well-preserved floras dominated by angiosperms from the Early Cretaceous. Very little is generally known of plant–arthropod and plant–pathogen interactions from compression–impression floras of the mid Mesozoic [7], including the time interval during initial angiosperm diversification [8]. This is the sole deposit examined intensively for the entire Cretaceous Period employing the functional feeding group–damage type (FFG–DT) system [3] for fossil herbivory analysis. Although several studies provide insight into arthropod herbivory during this time interval, they are either based on a restricted number of DTs and low sample size, such as the Crato [9,10] and Soap Wash [11] localities, or the locality has a robust sample size, such as the Hatira plant assemblage of Israel [12], but the FFG–DT system was not used, and instead arthropod and fungal damage was determined by a coarser grained ichnotaxonomic classification.
• The combination of six metrics for assessing herbivory provide the most accurate snapshot of herbivore feeding guilds to date of a single plant assemblage in the fossil record. One of these metrics, feeding event occurrences, is a detailed method of assessing arthropod herbivory intensity at the level of an individual feeding session at a moment in time by an individual arthropod feeding on its host-plant organ, such as a leaf. Feeding event occurrences exceed the value of herbivorized surface area in capturing the extent of herbivory on plant hosts. Although used in other studies of plant–arthropod interactions [13], non-metric multidimensional scaling provides alternative data to that of feeding event occurrences by highlighting the relationships between surface area of plant clades/groups at the level of the entire plant assemblage.
• The presence of a pattern of arthropod damage on successive floras can document longer-term herbivory trends [13] that can be used to test various hypotheses, such as the trophic feedback of arthropod herbivores to a changing regional flora during the Paleocene–Eocene Thermal Maximum [14], or the responses of arthropod herbivores to the ecological crises at the Cretaceous–Paleogene [15] and Permian–Triassic boundaries [16]. For the current study, several analyses (e.g., [17]) based on compilations of arthropod family diversity through time at the level of the geologic stage indicate that the diversity of arthropods, including herbivores, failed to increase during the 30 million-year-long interval of initial angiosperm diversification. This taxic-based hypothesis has never been tested using plant–arthropod interaction data from diverse, well-preserved, and temporally relevant fossil floras. The examination of the 103 million-year-old Rose Creek plant assemblage is part of a comparative study examining arthropod and fungal herbivory compared to a 22 million-year-older, gymnosperm dominated flora of the Yixian Formation from Northeastern China.
• Finally, these data are of interest to a broad community of ecologists, botanists, entomologists, paleobotanists, paleoentomologists and particularly researchers who study fossil and modern plant–arthropod associations.

## 1 Data Description

The following account describes the sequence of appendices in the online supplementary material related to data, methods, metrics, and analyses supporting the Rose Creek study [1]. Appendices S1–S3 provide ancillary accounts at a global level of plant-organismic associations present during the Early Cretaceous pertaining to the Rose Creek locality. Appendix S4 displays the botanical context of the Rose Creek locality, whereas Appendices S5–S10 discuss the procedures for processing and databasing the Rose Creek plant assemblage, including criteria for establishing plant taxa, distinguishing herbivory from detritivory, rarefaction analyses of the plant specimens and damage types (DTs), and a recent study of arthropod damage on Rose Creek flowers [5]. The rarefaction analyses of plant specimens and damage types versus surface areas (Appendix S9) indicate that sampling is adequate. Appendices S11, S12, and S16 supply the raw data of functional feeding groups, DTs, and DT host-plant specialization. Photographic images represent continuation of Figs. 2–6 of Xiao et al. [1], presented here as Appendix S13 with Figs. S1–S15 that documents photographically the extraordinary breadth of the functional feeding groups (FFGs) and damage types (DTs) at Rose Creek. Appendix S14 provides brief discussions of the fossil histories of the 11 functional feeding groups occurring at Rose Creek. The host specialization assignments for each damage type (DT) are listed in Appendix S15. The composition of the plant taxa included in the nonmetric multidimensional scaling analysis (Fig. 11) is provided in Appendix S17. Descriptions and metadata are presented for 14 new Rose Creek DTs that represent six FFGs, in Appendix S18. These new DTs will be included in forthcoming Version 4 of the *Guide to Insect (and Other) Damage Types on Compressed Plant Fossils* [3]. Appendices S19–S25 address in greater detail issues broached in the Discussion section of [1].

## 2 Experimental Design, Materials and Methods

During the 30 million-year-long interval (125–95 Ma) of angiosperm diversification, arthropod herbivores and pathogens diversified, based on a variety of independent phylogenetic evidence. However, currently there is no compelling evidence for substantially increased arthropod herbivore or pathogen damage to any early angiosperm flora at this time. For this reason, we have evaluated arthropod herbivore and pathogen damage on one of the earliest, angiosperm dominated localities that is well documented – the Rose Creek plant assemblage.

Data were collected from 2084 specimens from the Rose Creek plant assemblage of the Dakota Formation, near Fairbury, southeastern Nebraska, USA, and were analyzed. Rose Creek strata are composed of fine-grained sandstones, dark-hued siltstones, and gray claystones that occasionally display lignitic partings [6] and were deposited in a fluvial–deltaic–estuarine depositional system that received considerable freshwater input [2].

Rose Creek plant taxa were identified overwhelmingly based on previous research [2], with addition of new morphotypes. The abundance of each plant taxon/morphotype was tallied. In addition, plant taxa from several localities of the Dakota Formation were tabulated [18]. Herbivore arthropod and fungal damage on each plant item was assigned to a distinct FFG, DT [3], and feeding event occurrence, and the total and herbivorized areas in cm^2^ of each plant item was recorded. Host specificity was assessed by the three levels of DT host-plant specialization, with generalized assigned a value of 1, intermediate specificity a value of 2, and specialized a value of 3. The total and herbivorized surface area of each plant item was measured by *Image J*, and the resulting herbivory index was used to indicate herbivory intensity. Component community structure of the three most abundant plant taxa was comprehensively analyzed using the three metrics of DT frequency, surface area herbivorized, and feeding event occurrences. Rarefaction analyses of plant specimens and damage types versus surface area sampled across the Rose Creek plant assemblage was implemented through an R platform with *iNEXT* package. A non-metric multidimensional scaling analysis among FFGs and plant clades/groups was implemented by *R* with a *vegan* package. Evaluations of the Rose Creek arthropod herbivory with other fossil and modern plant species and assemblages were based on contrasts with herbivory indices and with the stoichiometry of major FFGs consisting of a composite external feeding, piercing and sucking, mining, and galling of well-documented modern plant species.

## Ethics Statement

This paper and its online supplementary material are not currently being considered for publication elsewhere.

## CRediT authorship contribution statement

**Lifang Xiao:** Methodology, Formal analysis, Data curation, Writing – original draft, Writing – review & editing. **Conrad C. Labandeira:** Conceptualization, Methodology, Formal analysis, Data curation, Writing – review & editing, Supervision. **David L. Dilcher:** Methodology, Resources, Data curation, Writing – review & editing. **Dong Ren:** Data curation, Writing – review & editing, Supervision.

## Declaration of Competing Interest

The authors declare that they have no known competing financial interests or personal relationships which have, or could be perceived to have, influenced the work reported in this article.

## Data Availability

Data, metrics, and methods for arthropod and fungal herbivory at the dawn of angiosperm diversification: The Rose Creek plant assemblage of Nebraska, U.S.A. (Original data) (Mendeley Data).

## Appendix Supplementary Materials

Image, application 1

Image, application 2

Image, application 3

## References

[1] Xiao L., Labandeira C., Dilcher D., Ren D. (2021). Arthropod and fungal herbivory at the dawn of angiosperm diversification: the Rose Creek plant assemblage of Nebraska, U.S.A. Cretac. Res..

[2] Upchurch G.R., Dilcher D.L. (1990). Cenomanian angiosperm leaf megafossils, Dakota Formation, Rose Creek locality, Jefferson County, southeastern Nebraska. U.S. Geol. Surv. Bull..

[3] Labandeira C.C., Wilf P., Johnson K.R., Marsh F. (2007). https://profiles.si.edu/display/sro_20734.

[4] Carvalho M.R., Wilf P., Barrios H., Windsor D.M., Currano E.D., Labandeira C.C., Jaramillo C.A. (2014). Insect leaf-chewing damage tracks herbivore richness in modern and ancient forests. PLoS One.

[5] Xiao L.F., Labandeira C.C., Dilcher D.L., Ren D. (2021). Florivory of Early Cretaceous flowers by functionally diverse insects: implications for early angiosperm pollination. Proc. R. Soc. B Biol. Sci..

[6] Retallack G.J., Dilcher D.L. (2012). Outcrop versus core and geophysical log interpretation of Mid-Cretaceous paleosols from the Dakota Formation of Kansas. Palaeogeogr. Palaeoclim. Palaeoecol..

[7] Pinheiro E.R.S., Iannuzzi R., Duarte L.D.S. (2016). Insect herbivory fluctuations through geological time. Ecology.

[8] Labandeira C.C. (2013). A paleobiologic perspective on plant–insect interactions. Curr. Opin. Plant Biol..

[9] Pires E.F., Sommer M.G. (2009). Plant–arthropod interaction in the Early Cretaceous (Berriasian) of the Araripe Basin. Brasil. J. S. Am. Earth Sci..

[10] Filho E.B.D.S., Adami-Rodrigues K., Lima F.J.D., Bantim R.A.M., Wappler T., Saraiva A.A.F. (2017). Evidence of plant–insect interaction in the Early Cretaceous flora from the Crato Formation, Araripe Basin, Northeast Brazil. Hist. Biol..

[11] Arens N.C., Gleason J.B. (2016). Insect folivory in an angiosperm-dominated flora from the Mid-Cretaceous of Utah, USA. Palaios.

[12] Krassilov V.A., Rasnitsyn A. (2008).

[13] Maccracken S.A., Labandeira C.C. (2020). The middle Permian south ash pasture assemblage of north-central Texas: coniferophyte and gigantopterid herbivory and longer-term herbivory trends. Int. J. Plant Sci..

[14] Currano E.D., Wilf P., Wing S.L., Labandeira C.C., Lovelock E.C., Royer D.L. (2008). Sharply increased insect herbivory during the Paleocene–Eocene Thermal Maximum. Proc. Natl. Acad. Sci. USA.

[15] Wilf P., Labandeira C.C., Johnson K.R., Ellis B. (2006). Decoupled plant and insect diversity after the end-Cretaceous extinction. Science.

[16] Labandeira C.C., Kustatscher E., Wappler T. (2016). Floral assemblages and patterns of insect herbivory during the Permian to Triassic of Northeastern Italy. PLoS One.

[17] Labandeira C.C., Sepkoski J.J. (1993). Insect diversity in the fossil record. Science.

[18] Wang H.S. (2002).

