# Supplementary material for: Data, metrics, and methods for arthropod and fungal herbivory at the dawn of angiosperm diversification: The Rose Creek plant assemblage of Nebraska, U.S.A
Source: Data Brief. 2022 Apr 14;42:108170. doi: 10.1016/j.dib.2022.108170 (PMC9058965; doi:10.1016/j.dib.2022.108170)
Supplement: Supplementary file 1 [file mmc1.docx]

ONLINE SUPPLEMENTARY MATERIAL

**Arthropod and fungal herbivory at the dawn of angiosperm diversification:**

**The Rose Creek plant assemblage of Nebraska, U.S.A.**

Lifang Xiao^1,2^, Conrad C. Labandeira^1–3*^, David L. Dilcher^4^,

and Dong Ren^1*^

^1^College of Life Sciences and Academy for Multidisciplinary Studies, Capital Normal University, 100048, Beijing, China;

^2^Department of Paleobiology, National Museum of Natural History, Smithsonian Institution, Washington, DC 20013, USA;

^3^Department of Entomology, University of Maryland, College Park, MD 20742, USA;

^4^Department of Biology, Indiana University, Bloomington, IN 47405, USA.

* Correspondence Authors: DR, [rendong@cnu.edu.cn](mailto:rendong@cnu.edu.cn) ; CCL, [labandec@si.edu](mailto:labandec@si.edu)

**Outline**

**Overview**………………………………………………………………..………………………….. 4

**Appendix S1:** Angiosperm associated lineages present during the

Early Cretaceous……………………………………………………………………………….… 4

**(a)** Plants………………………………………………………………………………………….. 4

**(b)** Arthropods………………………………………………………………………………….... 5

**(c)** Fungi…………………………………………………………………………………………... 5

**Appendix S2****:** Arthropod and pathogen interactions with plants during

early angiosperm diversification………………………………………………………..…….… 5

**(a)** The Crato, Soap Wash, Comox, and Valizhgen plant assemblages…………….……. 5

**(b)** Mid-Cretaceous floras from Israel………………………………………………….……… 6

**Appendix S3:** Historical backdrop to the current study………………………………………... 6

**Appendix S4:** List of plant taxa from the Dakota Formation by locality……………………… 8

**Appendix S5:** Frequency distribution of the Rose Creek plant assemblage………………. 13

**Appendix S6:** Procedures for documenting herbivory on plants……………………………. 14

**(a)** Initial procedures……………………………………………………………………………. 14

**(b)** Database structure…………………………………………………………………………. 14

**(c)** Photographic documentation……………………………………………………………… 14

**(d)** Herbivorized surface area metric…………………………………………………………. 14

**(e)** Feeding event occurrences metric……………………………………….…….…………. 15

**Appendix S7:** Criteria for establishing plant taxa……………………………………………... 16

**Appendix S8:** Criteria for recognizing herbivory from detritivory……………………………. 16

**(a)** Reaction tissue……………………………………………………………………………… 16

**(b)** Stereotyped damage pattern……………………………………………………………… 17

**(c)** Microstructural features……………………………………………………………………. 17

**(d)** Tissue, organ, or species specificity……………………………………………………… 17

**(e)** Ecological uniformitarianism………………………………………………………………. 17

**Appendix S9:** Rarefaction analyses of the Rose Creek plant assemblage………………... 18

1. Rarefaction–extrapolation curves of specimen number vs surface area

(cm^2^) for the Rose Creek plant assemblage and its major plant clades……………………... 18

**(b)** Rarefaction–extrapolation curves of specimen number vs surface area

(cm^2^) for the Rose Creek plant assemblage and its major species………………………….…. 18

**(c)** Rarefaction–extrapolation curves of damage types (DTs) vs surface area

(cm^2^) for the Rose Creek plant assemblage and its major plant clades……………………... 19

**(d)** Rarefaction–extrapolation curves of damage types (DTs) vs surface area

(cm^2^) for the Rose Creek plant assemblage and its major species………………………….…. 19

**Appendix S10:** Evaluation of Rose Creek florivory………………………………………….. 20

**Appendix S11**: Functional feeding groups (FFGs) and feeding classes…………………… 20

**Appendix S12:** Raw data of plant clades/species showing feeding class,

functional feeding group, damage type, host specificity, and feeding

event occurrences……………………………………………………………….…………….. 21

**Appendix S13:** Continuation of photographic documentation of herbivory……………….. 22

**(a)** **Fig. S1:** Component herbivore community of *Pandemophyllum*

*kvacekii* I……………………………………………………………………………………. 22

**(b)** **Fig. S2:** Component herbivore community of *Pandemophyllum*

*kvacekii* II…………………………………………………………………………………… 23

**(c)** **Fig. S3:** Component herbivore community of *Pandemophyllum*

*kvacekii* III…………………………………………………………………………………... 24

**(d) Fig. S4:** Component herbivore community of *Pandemophyllum*

*kvacekii* IV………………………………………………………………………………….. 25

**(e) Fig. S5:** Component herbivore community of *Pabiania variloba* I………………...…. 26

**(f)** **Fig. S6:** Component herbivore community of *Pabiania variloba* II………………...… 27

**(g) Fig. S7:** Component herbivore community of *Pabiania variloba* III…………..…...…. 28

**(h) Fig. S8:** Component herbivore community of *Pabiania* *variloba* IV……………….…. 29

**(i) Fig. S9:** Component herbivore community of *Pandemophyllum*

*attenuatum*…………………………………………………………………………………. 30

**(j) Fig. S10:** Component herbivore community of *Densinervum kaulii*…………………. 31

**(k) Fig. S11:** Component herbivore community of *Anisodromum* *wolfei*………………… 32

**(l) Fig. S12:** Component herbivore community of New Genus A and

*Didromophyllum* *basingeri*………….………………………..…………………………… 33

**(m) Fig. S13:** Component herbivore communities of *Pandemophyllum*

sp. 1, *Acritodromum* *ellipticum*, and *Reynoldsiophyllum* *nebrascense*……………… 34

**(n) Fig. S14:** Herbivore associations on unidentified plant hosts I…………….………… 35

**(o) Fig**. **S15:** Herbivore associations on unidentified plant hosts II…………………..….. 36

**Appendix S14:** Brief fossil records of functional feeding groups……………………………. 37

**(a) Hole feeding**………………………………………………………………………………. 37

**(b) Margin feeding**……………………………………………………………………………. 37

**(c) Skeletonization**…………………………………………………………………………... 37

**(d) Surface feeding**…………………………………………………………………………… 37

**(e) Oviposition**………………………………………………………………………………… 38

**(f) Piercing and sucking**……………………………………………………………………. 38

**(g) Mining**………………………………………………………………………………………. 38

**(h) Galling**……………………………………………………………………………………… 39

**(i) Seed predation**……………………………………………………………………………. 39

**(j) Borings**…………………………………………………………………………………….. 39

**(k) Pathogens**…………………………………………………………………………….…… 40

**Appendix S15:** Host-plant specialization data. Generalized, intermediate

and specialized damage types (Ds) on host plants………………………………………… 41

**Appendix S16:** Percent removal of host plant herbivorized surface area…………………. 44

**Appendix S17:** Composition of plant taxa analyzed by NMDS……………………………... 47

**Appendix S18:** New damage type (DT) descriptions………………………………………… 47

**(a) DT276** (Surface feeding)………………………………………………………………….. 47

**(b) DT383** (Piercing and sucking)……………………………………………………………. 48

**(c) DT392** (Piercing and sucking)……………………………………………………………. 49

**(d) DT234** (Mining)…………………………………………………………………………….. 49

**(e) DT386** (Galling)……………………………………………………………………………. 50

**(f) DT398** (Galling)……………………………………………………………………………. 51

**(g) DT160** (Borings)…………………………………………………………………………… 51

**(h) DT174** (Pathogens)……………………………………………………………………….. 52

**(i) DT242** (Pathogens)……………………………………………………………………….. 52

**(j) DT381** (Pathogens)……………………………………………………………………….. 53

**(k) DT382** (Pathogens)……………………………………………………………………….. 52

**(l) DT385** (Pathogens)……………………………………………………………………….. 54

**(m) DT387** (Pathogens)……………………………………………………………………….. 55

**(n) DT388** (Pathogens)……………………………………………………………………….. 56

**Appendix S19:** Herbivorized surface area comparisons. Rose Creek

(A) compared to equivalent data from fossil (B) and modern (C–I)

studies of individual species and bulk floras…………….………………………………….. 57

**Appendix S20:** Reasons for elevated herbivory levels at Rose Creek……………………. 60

**(a)** Methodological issues…………………………………………………………………….. 60

**(b)** Intrinsic elevated herbivory richness…………………………………………………….. 60

**Appendix S21:** Five methodological reasons for elevated damage

type (DT) diversity at Rose Creek……………………………………………………………. 61

**Appendix S22:** The persistence of herbivore component communities……………….….. 62

**Appendix S23:** Documentation for Figure 12–Herbivore component

communities of five modern plant hosts……………………………………………………… 63

**Appendix S24:** Modern methods of assessing arthropod herbivory………………….…… 66

**Appendix S25:** Hypotheses explaining modern plant–arthropod herbivory………….…… 66

**Supplementary material references**…………………………………………………………. 67

**Overview**

Appendices of the Supporting Information furnish data, extended documentation, and a review of the relevant literature that bolster various aspects of the plant–arthropod interactions of the Rose Creek locality presented in the main text (Xiao et al., 2021c) and linked *Data in Brief*. Appendices S1 to S3 provide ancillary accounts at a global level of plant-organismic associations present during the Early Cretaceous relevant to the Rose Creek locality. Appendix S4 displays the botanical context of the Rose Creek locality, whereas Appendices S5–S10 discuss the procedures for processing and databasing the Rose Creek plant assemblage, including criteria for establishing plant taxa and distinguishing herbivory from detritivory, rarefaction analyses of the plant specimens and damage types (DTs), and a recent study of arthropod damage on Rose Creek flowers. The rarefaction analyses of plant specimens and damage types versus surface areas (Appendix S9) indicate adequate sampling. Appendices S11, S12 and S16 supply the basic data of functional feeding groups, raw plant damage, and host specificity. Photographic images represent continuation of Figures 2 to 6 of the main text (Xiao et al., 2021c), presented here as Appendix S13 with figures S1–S15 that documents photographically the extraordinary breadth of the functional feeding groups (FFGs) and damage types (DTs). Appendix S14 provides brief discussions of the fossil histories of the 11 functional feeding groups occurring at Rose Creek. The host specialization assignments for each damage type (DT) are listed in Appendix S15. The composition of the plant taxa included in the nonmetric multidimension analysis (Fig. 11) (Xiao et al., 2021c) is provided in Appendix S17. Descriptions and data are presented for the 14 new Rose Creek DTs, representing six FFGs, in Appendix S18. These new DTs will be included in forthcoming Version 4 of the *Guide to Insect (and Other) Damage Types on Compressed Plant Fossils* (Labandeira et al., 2007). Appendices S19–S25 address in greater detail issues broached in the Discussion section of the manuscript.

**Appendix S1:** **Angiosperm associated lineages present during the Early Cretaceous**

**(a) Plants**

There is a broad consensus regarding the general sequence of appearance of eleven, earliest (or basal) appearing lineages of angiosperms (Wikström et al., 2001; Soltis and Soltis, 2004; Magallón and Castillo, 2009; Bell et al., 2010; Doyle, 2012), and to a lesser extent other lineages (Zeng et al., 2014). The sequence of (*Amborella* + Nymphaeales) + [Austrobaileyales + {Chloranthaceae + monocot clade} + {⁅Canellales + Piperales⁆ + ⁅Magnoliales + Laurales⁆} + {Ceratophyllales + eudicots}] is a consistent pattern of early angiosperm cladogenesis. Exceptions to this sequence include positioning of the Chloranthaceae relative to the monocot clade that is one or two nodes removed in two studies, and if the Hydatellaceae was included in the analysis of most studies.

**(b) Arthropods**

Principal plant-associated arthropod lineages that diversified during the late Early Cretaceous were almost all the modern groups. Diversifying groups included grasshoppers, crickets and katydids (Orthoptera: Ensifera and Caelifera) (Song et al., 2015); jumping plant lice (Hemiptera: Psyllidae) (Hodkinson, 1985); scale insects (Hemiptera: Coccoidea) (Vea and Grimaldi, 2016); planthoppers, leafhoppers and treehoppers (Hemiptera: Auchenorrhyncha) (Szwedo, 2002; Song and Liang, 2013); stink bugs, squash bugs, flat bugs and seed bugs (Hemiptera: Pentatomorpha) (Yao et al., 2012); metallic wood-boring beetles (Coleoptera: Buprestidae) (Evans et al., 2015); the four major beetle (Coleoptera) lineages of Tenebrionoidea (darkling beetles and relatives), Scarabaeoidea (scarab beetles and relatives), Chrysomeloidea (leaf and longhorn beetles), and Curculionoidea (weevils) (McKenna et al., 2009; Wang et al., 2013a; but see Gómez-Zurita et al., 2007); ditrysian moths (Lepidoptera: Ditrysia) (Wahlberg et al., 2013); sawflies (Hymenoptera: Tenthredinoidea) (Leppanen et al., 2012; Isaka and Sato, 2015); and mites (Krantz and Lindquist, 1999). Based on molecular data, some lineages, such as leaf-rolling moths (Lepidoptera: Tortricidae) (Fagua et al., 2017), and seed beetles (Coleoptera: Bruchinae) (Kergoat et al., 2014), likely diversified after the initial expansion of angiosperms, and time lags are known to occur between the origin of a potential host-plant lineage and the time that they are colonized (Percy et al., 2004; Lopez-Vaamonde et al., 2006). Pollinator lineages (Xiao et al., 2021a) that often involve foliage feeding, include tumbling flower beetles (Coleoptera: Mordellidae) (Bao et al., 2019); short-winged flower beetles (Coleoptera: Kateretidae) (Peris et al., 2020); mosquitoes (Diptera: Culicidae) (Hartkopf-Fröder et al., 2011); aculeate wasps (Hymenoptera: Aculeata) (Grimaldi et al., 2019); and an early bee (Hymenoptera: Apoidea) (Danforth and Poinar, 2011).

**(c) Fungi**

Many pathogens are intricately associated with angiosperms (Dick, 1982; Liu et al., 2015), a feature documented for the Early Cretaceous. The dominant, identifiable groups are at Rose creek are the Ascomycota and Basidiomycota (Labandeira and Prevec, 2014). In particular, the diversification of Agaricomycetes, especially taxa involving ectomycorrhizal associations, occurred during this interval (Lutzoni et al., 2018).

**Appendix S2:** **Arthropod and pathogen interactions with plants during early angiosperm diversification**

**(a)** **The Crato, Soap Wash, Comox, and Valizhgen plant assemblages**.

Several studies illuminate certain aspects of arthropod herbivory at this time. The Crato plant assemblage, of mid Aptian age (ca 118 Ma), from the Araripe Basin of Northeast Brazil, is perhaps the best-studied flora, as documented by studies that examine limited numbers of specimens (Pires and Sommer, 2009; Filho et al., 2017). These studies show a richness of arthropod damage, including margin feeding, skeletonization, oviposition, leaf mines, and galls, but the specimen sample levels are very low. The Soap Wash plant assemblage, occurring at the Albian–Cenomanian boundary (100 Ma) of central Utah, U.S.A., is a moderately sampled and evaluated flora, consisting of 50 arthropod-herbivore damage types representing hole feeding, margin feeding, skeletonization, surface feeding, and piercing and sucking – but no endophytic damage – that was found on 152 leaves identifiable to a plant host taxon or morphotype (Arens and Gleason, 2016). The study evaluated arthropod-damaged leaves for damage-type richness, percentage of leaf surface area herbivorized, host specificity level, and the effect of leaf mass per area, a measure of palatability to folivores. Other, more limited studies examined arthropod interactions on a plant species from a single locality, such as borings from the seed cone of the conifer *Acanthostrobus* *edensis* Klymiuk, Stockey & Rothwell from the Turonian Comox Formation of British Columbia, Canada (Klymiuk et al., 2015). Occasionally, the description of a single notable interaction on a plant specimen was made, such as the ichnotaxon *Paleogallus* *kamchaticus* Vasilenko, Maslova et Herman, a gall from Turonian Valizhgen Formation of northwestern Kamchatka, Russia (Vasilenko et al., 2016).

**(b)** **Mid-Cretaceous floras from Israel**

The most extensive study has been an examination of the lower to middle Albian (ca. 110 Ma) Hatira plant assemblage at Makhtesh Ramon of the northern Negev and the mid Turonian (92 Ma) Ora plant assemblage at Gerofit, in southern Israel (Krassilov et al., 2004, 2007; Krassilov, 2008a, 2008b; Krassilov and Rasnitsyn, 2008; Krassilov and Shuklina, 2008). This study (Krassilov and Rasnitsyn, 2008) temporally brackets and is analogous to the Rose Creek plant assemblage of late Albian age (103 Ma) presented here, although the methods of documentation and analysis are considerably different. The Gerofit plant assemblage is highly diverse, represented by 50 plant species that represent 16 major lineages of angiosperms that inhabited mangrove, marsh, aquatic and mesic woodland habitats. Plant hosts from both Israeli floras harbor 28 ichnogenera and 74 ichnospecies of damage of representing a wide variety of oviposition (five ichnogenera, 12 ichnospecies), galling (ten ichnogenera, 25 ichnospecies), mining (11 ichnogenera, 28 ichnospecies), as well as lesser amounts for hole feeding, piercing and sucking, and larval leaf cases. Foliar margin feeding, skeletonization and surface feeding are present but lack formal designations. Arthropod damage of the Ora and Gerofit plant assemblages was classified using an ichnotaxonomic system (Krassilov and Rasnitsyn, 2008) that is independent of damage-type system presented here (Labandeira et al., 2007), and supplemented by subsequently published addenda (Currano et al., 2008; Sarzetti et al., 2009; Wang et al., 2009; Wing et al., 2009; Wappler et al., 2009, 2012; Winkler et al., 2010; Stull et al., 2013; Carvalho et al., 2014; Labandeira, 2014; Schachat et al., 2014, 2015; Ding et al., 2014, 2015; Donovan et al., 2014, 2016; Meng et al., 2017; Labandeira et al., 2018; Xu et al., 2018; Lin et al., 2019; Correia et al., 2020).

**Appendix S3:** **Historical backdrop to the current study**

The testing of the hypothesis—the ecologic expansion of angiosperms during the Early Cretaceous had no effect on herbivory levels when gymnosperms were dominant—requires that a study analogous to that of Rose Creek be implemented on an earlier, Early Cretaceous gymnosperm-dominated plant assemblage. Such a study has been completed (Xiao et al., 2022) for a well-documented, diverse, abundant, and well-preserved gymnosperm flora for an evaluation of the proposed before-and-after contrast. The broader, theoretical motivation for a test of this hypothesis is to use the results of the plant–arthropod associational data from these two studies that contrast in approach with many earlier, taxic-based, studies that indicate arthropod family-level diversity did not increase with the Cretaceous expansion of angiosperms from 130 to 90 Ma. These taxic studies extend back about 30 years and require a brief historical overview.

A study documenting the global family-level diversity of insects from the Early Devonian about 410 million years ago to the Holocene Epoch 11 thousand years ago indicated an absence in insect diversification that paralleled the dramatic evolutionary and ecological expansion of angiosperms (Labandeira and Sepkoski, 1993). However, this flat trendline of insect diversity during the 130-to-90-million-year interval of angiosperm diversification was highly contentious (Grimaldi, 1999; Crepet, 2000). Notably, an earlier study (Dmitriev and Zherikhin, 1988) and subsequent studies (Jarzembowski and Ross, 1993, 1996; Alekseev et al., 2001) demonstrated the same pattern, based largely on independently assembled datasets (Rasnitsyn, 1988; Dmitriev and Zherikhin, 1988; Labandeira, 1994; Jarzembowski and Ross, 1996). Recently, this pattern has been upheld by an updated family-level dataset (Condamine et al., 2016) and an analysis using a methodologically different capture-mark-recapture approach (Schachat et al., 2019). A crucial aspect regarding this issue is the absence of any independent data on plant–insect interactions from well-preserved and diverse floras during the relevant time interval that potentially could address the above hypothesis. The issue specifically is whether increases in the richness, intensity, host-specialization and component-community structure of insect (and mite) herbivores and pathogens significantly differed during this interval from a gymnosperm-dominated flora of the “before” study (Xiao et al., 2022) to an angiosperm-dominated flora in the “after” study. The “after” study is our examination here of plant–arthropod interactions of the Rose Creek plant assemblage of the Dakota Formation.

**Appendix S4: List of plant taxa from the Dakota Formation by locality**

|  | **-----------------------Kansas----------------------** | | | | **----------Nebraska----------** | | | **Minnesota** |
| --- | --- | --- | --- | --- | --- | --- | --- | --- |
|  | Hosing-ton III | Acme Brick | Linnenber-  ger’s Ranch | Braun’s Ranch | Rose Creek | Pleasant Dale | Spring-field | Courtland |
| Megafloral Plant Taxa | |  |  |  |  |  |  |  |
| CHAROPHYTES |  |  |  |  |  |  |  |  |
| *Sphaerochara* sp. 1 |  |  |  |  | **X** |  |  |  |
| LYCOPODS |  |  |  |  |  |  |  |  |
| **Isoetales** |  |  |  |  |  |  |  |  |
| *Isoetites phyllophila* | X |  |  |  | **X** |  |  |  |
| *Isoetes* sp. 1 | X |  |  |  | **X** |  |  |  |
| HORSETAILS |  |  |  |  |  |  |  |  |
| **Equisetales** |  |  |  |  |  |  |  |  |
| *Equisetum burchardtii* |  | X |  |  |  |  |  |  |
| FERNS |  |  |  |  |  |  |  |  |
| **Schizaeales** |  |  |  |  |  |  |  |  |
| *Anemia dicksoniana* | X | X | X | X | **X** |  |  |  |
| *Anemia dakotensis* | X | X |  | X | **X** |  |  |  |
| *Anemia* sp. 1 |  | X |  |  |  |  |  |  |
| **Aspidales** |  |  |  |  |  |  |  |  |
| *Asplenium* sp*.* 1 |  |  |  |  | **X** |  |  |  |
| **Filicales** |  |  |  |  |  |  |  |  |
| *Cladophlebis constricta* |  |  |  | X | **X** |  |  |  |
| *Cladophlebis inclinata* |  |  |  |  | **X** |  |  |  |
| *Cladophlebis parva* |  |  |  |  | **X** |  |  |  |
| *Cladophlebis* sp. 1 |  |  |  |  | **X** |  |  |  |
| **Dicksoniales** |  |  |  |  |  |  |  |  |
| *Coniopteris hymenophylloides* | |  |  |  | **X** |  |  |  |
| **Gleicheniales** |  |  |  |  |  |  |  |  |
| *Gleichenia comptoniaefolia* | X | X | X | X | **X** |  |  |  |
| *Gleichenia delicatula* | X | X | X | X | **X** |  |  |  |
| *Gleichenia* sp. 1 |  |  |  |  | **X** |  |  |  |
| *Matonidium americanum* | |  |  |  | **X** |  |  |  |
| *Matonidium brownii* | X |  |  |  | **X** |  |  |  |
| **Salviniales** |  |  |  |  |  |  |  |  |
| *Marsilea johnhallii* | X |  |  |  |  |  |  |  |
| GYMNOSPERMS |  |  |  |  |  |  |  |  |
| **Pinales** |  |  |  |  |  |  |  |  |
| Conifer Cone | X | X | X | X | **X** | X |  | X |
| Conifer Foliage | X | X | X | X | **X** | X |  | X |
| *Brachyphyllum crassum* |  | X | X | X |  |  |  |  |
| *Brachyphyllum* sp. 1 |  | X | X | X |  |  |  |  |
| *Pinus* sp. 1 |  |  |  |  |  |  |  | X |
| *Dammarites caudatus* |  | X |  |  |  |  |  |  |
| **Cycadales** |  |  |  |  |  |  |  |  |
| Cycad Foliage |  | X |  |  |  |  |  |  |
| ANGIOSPERMS |  |  |  |  |  |  |  |  |
| **Austrobaileyales** |  |  |  |  |  |  |  |  |
| *Longstrethia varidentata* |  |  |  |  | **X** |  |  |  |
| *Longstrethia aspera* | X |  |  |  |  |  |  |  |
| **Chloranthales** |  |  |  |  |  |  |  |  |
| *Crassidenticulum cracendentis* | |  |  |  |  |  |  | X |
| *Crassidenticulum decurrens* | X |  |  | X | **X** |  |  |  |
| *Crassidenticulum* sp. |  |  |  | X | **X** |  |  | X |
| *Crassidenticulum trilobum* | X |  |  | X |  |  |  |  |
| *Crassidenticulum* cf. *trilobum* | X |  |  |  |  |  |  |  |
| *Crassidenticulum landisiae* | |  |  | X |  |  |  |  |
| *Yangia glandifolia* |  |  |  | X |  |  |  |  |
| *Densinervum kaulii* |  |  |  |  | **X** |  |  | X |
| *Landonia calophylla* |  |  |  |  | **X** |  |  |  |
| *Landonia callii* |  |  |  | X |  |  |  |  |
| **Laurales** |  |  |  |  |  |  |  |  |
| *Pandemophyllum attenuatum* | |  |  |  | **X** |  |  | X |
| *Pandemophyllum kvacekii* | |  | X |  | **X** |  |  | X |
| *Pandemophyllum* sp. 1 | X |  | X |  | **X** |  |  | X |
| *Pabiania variloba* | X |  |  |  | **X** |  | X |  |
| *Pabiania* sp. 1 | X |  |  |  | **X** |  |  |  |
| *Pabiania groenlandica* | X |  |  |  | **X** |  |  |  |
| *Pabiania* cf. *groenlandica* | X |  |  |  |  |  |  |  |
| *Setterholmia rotundifolia* | |  |  |  |  |  |  | X |
| *Setterholmia deleta* |  |  |  |  |  |  |  | X |
| *Manchesterii macrophylla* | |  |  |  |  | X |  | X |
| *Rogersia kansense* |  |  |  | X |  | X |  | X |
| *Rogersia dakotensis* | X |  |  |  |  |  |  | X |
| *Rogersia parlatorii* | X |  |  | X |  | X |  |  |
| *Rogersia* cf. *parlatorii* |  |  |  |  |  | X |  |  |
| *Rogersia lottii* |  |  |  | X |  |  |  |  |
| *Wolfiophyllum daphneoides* | |  |  | X |  |  |  |  |
| *Wolfiophyllum heigii* |  |  |  | X |  |  |  |  |
| *Wolfiophyllum pfaffianum* | X |  |  |  |  |  |  | X |
| **Magnoliales** |  |  |  |  |  |  |  |  |
| New Genus A |  |  |  |  | **X** |  |  |  |
| *Liriophyllum siemia* |  |  |  |  |  |  |  | X |
| *Liriophyllum kansense* | X |  | X |  |  |  |  |  |
| *Jarzenia kanbrasota* | X |  |  |  |  | X |  | X |
| *Dicotylophyllum angularis* | |  |  |  | **X** |  |  |  |
| *Didromophyllum basingerii* | |  |  |  | **X** |  |  |  |
| *Reynoldsiophyllum masonii* | |  |  |  | **X** |  |  |  |
| *Reynoldsiophyllum nebrascense* | |  |  |  | **X** |  |  |  |
| **Rosidae** |  |  |  |  |  |  |  |  |
| *Anisodromum wolfei* | X |  |  |  | **X** |  |  |  |
| *Anisodromum upchurchii* | X |  |  |  |  |  |  |  |
| *Anisodromum schimpero* | X |  |  |  |  |  |  |  |
| *Citrophyllum aligera* | X |  |  |  |  |  |  |  |
| *Citrophyllum doylei* |  |  |  |  | **X** |  |  |  |
| *Dilcherocarpon combretoides* | |  |  | X |  |  |  |  |
| **Magnoliopsida unplaced** |  |  |  |  |  |  |  |  |
| *Acritodromum ellipticum* |  |  |  |  | **X** |  |  |  |
| *Aspidiophyllum denticulatum* | |  |  | X |  |  |  |  |
| *Wingia expansolobum* | X |  |  |  | **X** |  |  |  |
| *Wingia* cf. *expansolobum* | X |  |  |  |  |  |  |  |
| *Dicotylophyllum microserratum* | |  |  |  | **X** |  |  |  |
| *Dicotylophyllum myrtophylloides* | |  |  |  | **X** |  |  |  |
| *Dicotylophyllum rosafluviatilis* | |  |  |  | **X** |  |  |  |
| *Dicotylophyllum leptovenum* | X |  |  |  |  |  |  | X |
| *Dicotylophyllum carlsonii* |  |  |  |  |  |  |  | X |
| *Dicotylophyllum crasseprimus* | |  |  |  |  |  |  | X |
| *Dicotylophyllum* sp. 1 | X |  | X | X |  |  |  |  |
| *Dicotylophyllum tulipiferum* | |  |  |  |  |  |  | X |
| New Genus B |  |  |  |  | **X** |  |  |  |
| *Paleonelumbo macroloba* | X |  |  |  |  |  |  |  |
| *Trochodendroides* sp. 1 |  |  |  |  |  |  |  | X |
| *Crepetia minudentis* |  |  |  |  |  |  |  | X |
| *Gooleria crasseprima* |  |  |  |  |  |  |  |  |
| *Paleonelumbo macroloba* | X |  |  |  |  |  |  |  |
| *Glandilunatus kansense* |  |  | X |  |  |  |  | X |
| *Skogia leptoselis* | X |  |  |  |  |  |  |  |
| *Sungia delicatus* |  |  |  |  |  | X |  |  |
| **Ceratophyllales** |  |  |  |  |  |  |  |  |
| *Donlesia dakotensis* |  | X |  | X | **X** |  |  |  |
| **Proteales** |  |  |  |  |  |  |  |  |
| *Aquatifolia fluitans* | X |  |  |  |  |  |  |  |
| *Nelumbites crassinervum* | X |  |  |  |  |  |  |  |
| *Nelumbites farleyi* | X |  |  |  |  |  |  |  |
| *Sapindopsis bagleyae* | X |  |  |  |  |  |  |  |
| *Sapindopsis beekeria* | X |  |  |  |  |  |  |  |
| *Sapindopsis powelliana* | X |  |  |  |  |  |  |  |
| *Sapindopsis retallackii* | X |  |  |  |  | X |  |  |
| *Sapindopsis* sp. 1 | X |  | X |  |  |  |  |  |
| *Credneria quadratus* |  |  |  | X |  |  |  |  |
| *Credneria cyclophylla* | X |  |  |  |  |  |  | X |
| *Credneria* cf. *cyclophylla* | |  |  |  |  | X |  |  |
| *Dischidus quinquelobus* |  |  |  | X |  |  |  |  |
| *Eoplatanus serrata* |  |  |  | X |  |  |  |  |
| *Aspidiophyllum denticulatus* | |  |  | X |  |  |  |  |
| *Aspidiophyllum obtusum* |  |  |  |  |  |  | X |  |
| *Eurylobum dentatum* |  |  |  |  |  |  | X |  |
| *Platanus* sp. 1 | X |  |  | X |  |  |  |  |
| **Saxifragales** |  |  |  |  |  |  |  |  |
| *Trochodendroides elliptica* | |  |  | X |  |  |  |  |
| *Trochodendroides rhomboideus* | |  |  |  |  | X |  | X |
| *Dicotylophyllum aliquantuliserratum* | | |  |  | **X** |  |  |  |
| *Dicotylophyllum braunii* |  |  |  | X |  |  |  |  |
| *Dicotylophyllum fragilis* |  |  |  | X |  |  |  |  |
| *Dicotylophyllum coughlantii* | |  |  |  |  |  | X |  |
| *Dicotylophyllum huangia* |  |  |  | X |  |  |  |  |
| *Dicotylophyllum skogii* | X |  |  |  |  |  |  |  |
| *Hickeyphyllum sandersia* |  |  |  | X |  |  |  |  |
| *Hickeyphyllum imhofia* |  |  |  | X |  |  |  |  |
| *Jaramillophyllum celatus* | X |  |  |  |  |  |  |  |
| *Kalymmanthus walkeri* | X |  |  |  |  |  |  |  |
| *Kladoneuron ravenia* |  |  |  | X |  |  |  |  |
| *Meiophyllum expansolobum* | X |  |  |  | **X** | X |  |  |
| *Meiophyllum kowalskiae* | X |  |  |  |  |  |  |  |
| ?*Menispermites* sp. 1 |  |  |  |  |  |  | X |  |
| *Quercophyllum tenuinerve* | |  |  |  |  | X |  |  |
| *Wingia anisos* | X |  |  |  |  |  |  |  |
| **Magnoliopsida indet** |  |  |  |  |  |  |  |  |
| *Dakotanthus cordiformis* | X |  |  |  | **X** |  |  |  |
| *Prisca reynoldsii* | X |  | X |  |  |  |  |  |
| *Lesqueria elocata* | X |  |  |  |  |  |  |  |
| *Archaeanthus linnenbergeri* | |  | X |  |  |  |  |  |
| *Brasenites kansense* | X |  |  |  |  |  |  |  |
| *Caloda delevoryana* | X |  |  |  |  |  |  |  |
| **Sum (133)** | 59 | 13 | 15 | 36 | **46** | 13 | 5 | 26 |
| Proportion (%) | 44.36 | 9.77 | 11.28 | 27.07 | **34.59** | 9.77 | 3.76 | 19.55 |

**Appendix S5: Frequency distribution of the Rose Creek plant assemblage**^1^


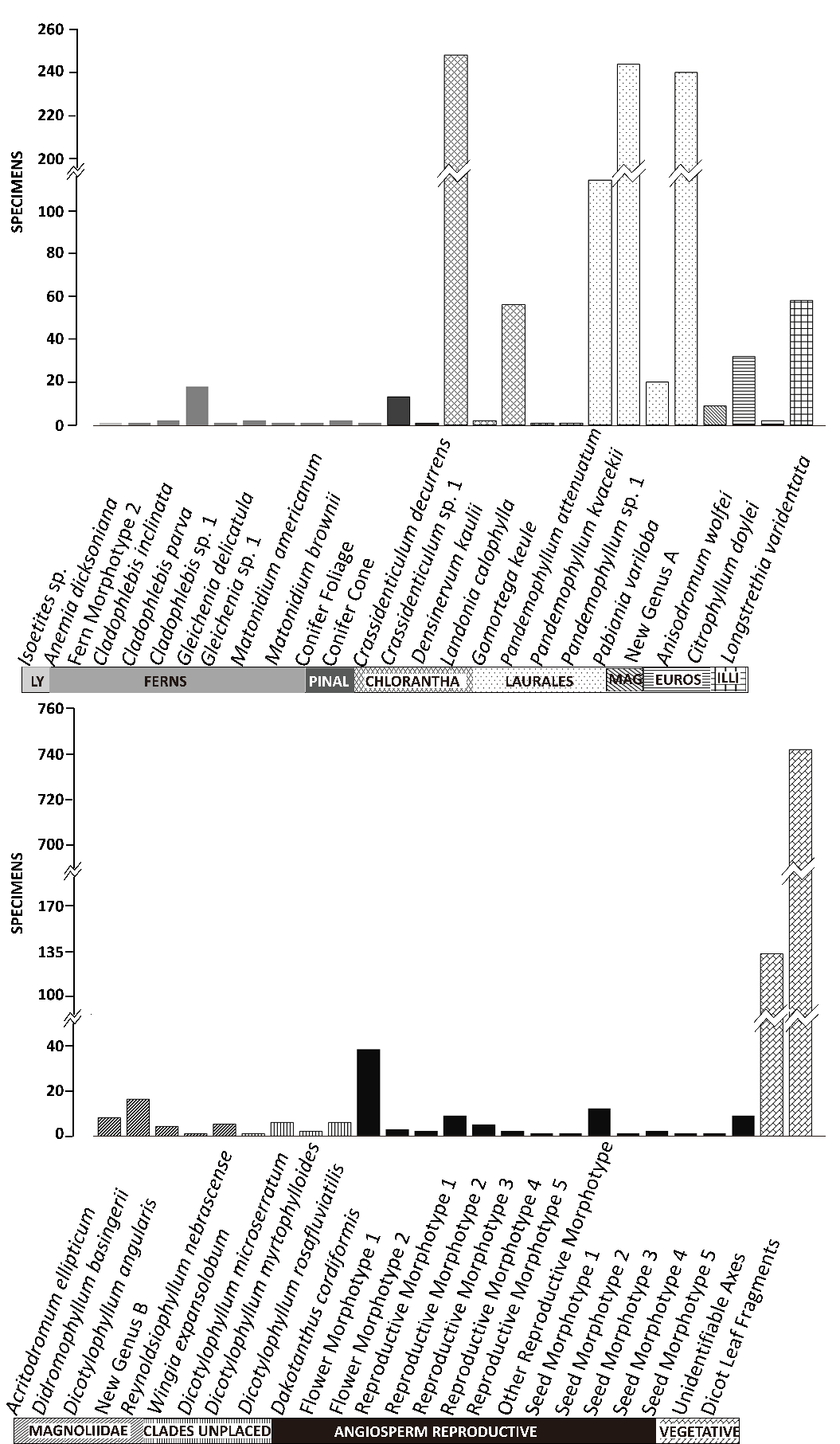


^1^Based on the 2084 specimens examined in this study.

**Appendix S6: Procedures and metrics for documenting herbivory on plants**

**(a) Initial procedures**

Specimens of the Rose Creek plant assemblage of the Dakota Formation, from the Florida Museum of Natural History of the University of Florida (UF), at Gainesville, Florida, were accessed for this study. Examination of specimens took place at the National Museum of Natural History, in Washington, DC, where they were on loan, and the balance of the flora was studied at Gainesville. An initial determination was made that excluded from identification and analyses poorly preserved specimens that did not retain delicate arthropod damage, such as punctures from piercing-and-sucking insects. Plant specimens that were well preserved but did not allow identification to genus and species or to morphotype, were retained and included in the specimen count. Fortunately, very few poorly preserved specimens were removed. After these specimens were culled, those that were greater in size than about 0.25 cm^2^ were included, and we considered all recognizable plant fragments—leaves, woody and non-woody axes, flowers, seeds and roots—as data. This left 353 mudstone slabs and 2084 plant specimens that formed the basis of the study.

**(b) Database structure**

The following identifications, measurements and comments were made for each examined plant specimen and entered into two Excel data files. (Details explaining the measurements are provided below) For the first Excel data file, we entered in each row identifications or data in the columns expressing (i) the UF specimen number; (ii) the examined plant species or morphotype; (iii) total specimen leaf area; (iv) herbivorized specimen leaf area (see [d] below); and (v) presence or absence of each of the 114 damage types (DTs) in the entire plant assemblage. For the second Excel file we listed along the top horizontally the 50 species/morphotypes and their ordinal-level taxonomic assignments; along the side vertically we listed the 114 damage types (DTs) by functional feeding group (FFG) and in an adjacent column the DT host specificity for each DT that was designated as 1 (generalist), 2 (intermediate specificity), or 3 (specialist) as established by Wilf and Labandeira (1999). In the resulting 50 x 114 matrix we entered, where relevant, the number of recorded feeding event occurrences for each DT by species/morphotype (see [e] below). This data matrix summarized the totals for feeding events by DT and FFG along the right vertical margin, and for feeding events by plant species/morphotypes and their assigned major plant groups along the bottom horizontal margin. This second data matrix had 5700 cells of which 6.70% were occupied with DT occurrences.

**(c) Photographic documentation**

Relevant and well-preserved arthropod and pathogen damage on all plant organs, such as leaves, axes, seeds and flowers, were documented with light microscopy. Macrophotographs were taken by a Canon 50D camera and a Canon EF-S60 mm f/2.8 macro lens using direct and indirect illumination from spot and strobe lights. Microphotographs were taken on the Nikon SMZ 25 microscope with a Nikon DS-Ri 2 digital camera system. Resulting photos were culled for quality and incorporated into figures using Adobe^®^ Illustrator and Photoshop software.

**(d) Herbivorized surface area metric**

The reconstructed total surface area and herbivorized surface area, if any, of each plant specimen was determined in a multistep process. (In this section, the term leaf can be substituted by another plant organs such as a stem or seed.) The first procedure was to photograph all individual plant specimens using a macrophotography setup. Later, a microphotography setup was used for detailed enlarged photos of the herbivorized leaves. These two instruments produced images that then were filtered to accentuate the leaf margin outlines by use of Adobe Illustrator ^®^ *Draw* (version 4.8) software (San Jose, California). For the second task, the outside margin of each plant specimen was digitized by use of *ImageJ*^®^ software of the National Institutes of Health (Bethesda Maryland). Those sections of the outer leaf edge missing due to herbivory, especially involving margin feeding, were reconstructed based on previous knowledge of the complete leaf outline. Third, the outer margins of the leaves were digitized, as was all instances of herbivory that previously was identified to damage type such as margin feeding, skeletonization, oviposition, mining and galling. To minimize measurement error, thin lines were used to outline the damaged areas. For example, the early stage of leaf mine DT41 is deployed as a thin, linear trajectory whose area was circumscribed by appropriately thin lines.

Estimating herbivorized areas for piercing and sucking was a challenging exercise. In most instances, it was important to distinguish complicated, numerous and miniscule areas of piercing-and-sucking punctures such as DT46, DT47 and DT48 from other DTs. It was decided that after the average area of each puncture DT was determined and counted, then it was multiplied by the number of counted or estimated punctures present. For punctures present as linear, curvilinear or looped tracks, such as DT138, DT184, and DT338, the number of actual punctures was counted (the npl function) for a given relevant DT on a leaf, and then the occurrences area of this DT was digitized (the ael function). For each of these DT occurrences the herbivorized area calculated was determined by ael x npl. Punctures patterned in densely clustered splotches such as DT281 and DT383 or DT330 blanketing major portions of a leaf were determined by digitizing the area of punctures in a 4 mm^2^ frame, which was multiplied by the number of 4 mm^2^ frames that encompassed the damaged area. Issues for estimating damaged area of piercing and sucking fortunately was not encountered by other FFGs, such as hole feeding and pathogen damage, for which direct measurement was used.

The fourth procedure was to enter, all values of total and herbivorized surface areas for each leaf in an Excel spreadsheet. The total leaf surface area consisted of the reconstructed leaf that included all subset areas attributed to herbivory. Fifth, digitized areas of each leaf were calculated for the total leaf area and the herbivorized fraction. Herbivorized areas were attributed to specific DTs and their associated FFGs for each plant specimen. Last, the total and herbivorized areas were assigned to all leaves that belonged to a species or morphotype and its affiliated taxonomic order. Total and herbivorized surface areas were summed for all species and morphotypes to provide the total and herbivorized values of the entire Rose Creek plant assemblage for calculation of the HI (herbivory index).

**(e) Feeding event occurrence metric**

Each DT is linked to one of the three categories of feeding event occurrences. Feeding event occurrences are classified as single, pattern, or bulk. The first category is a single feeding event or episode of plant consumption that results in one instance of damage. A single feeding event would consist of a single bite mark along an edge of a leaf, such as DT12, in the case of margin feeding (Gangwere, 1966), or a single, isolated puncture on a leaf in the example of DT46, for piercing-and-sucking (Whittaker, 1984).

The second category is a feeding event consisting of a pattern, which involves an episode of consumption where multiple instances of the same behaviorally linked damage occur within a confined area of the plant and is created by the same individual herbivore feeding at a moment in time. Examples of pattern feeding events are numerous vein-surrounded skeletonizations in a confined space on a leaf (DT20), produced in a single event by a skeletonizing insect (Martin, 2017). Another example is multiple parallel rows of closely spaced lesions (DT54) produced by an ovipositing dragonfly while perched at a single spot (Sarzetti et al., 2009). A feeding event producing multiple examples that result in a pattern of associated damage constitutes a single feeding event occurrence.

A third, rare category is a bulk feeding event that applies principally to compound mite or insect galls whose numerous, miniscule chambers within the gall structure are difficult to tabulate; Consequently, the gall is given an estimated figure of the number of chambers that reflect the number of individuals present. Each gall chamber that houses a feeding individual, constitutes a feeding event occurrence.

Although this process of tabulating individual feeding events can be tedious for single feeding events of piercing-and-sucking DTs and mite galls, it does provide a separate, most fine-grained and independent measure of herbivore intensity from the plant fossil record. Moreover, feeding event data tabulated as DT frequencies for particular plant-host taxa can provide interaction strengths for DT–plant host links in bipartite food webs (Dormann et al., 2008).

**Appendix S7: Criteria for establishing plant taxa**

Two philosophical approaches have been used to establish the identities of plants from the Rose Creek plant assemblage. The first approach involved assignment of fossil angiosperm leaves to modern genera based on superficial resemblance and often without critical examination of key features of fossil leaf architecture and cuticular anatomy (Upchurch and Dilcher, 1990). This process of “picture matching” (Dilcher, 1974) resulted in rarely recognizing extinct lineages (Wolfe, 1973; Dilcher, 1974; Dilcher and Kovach, 1986). Accordingly, rejection of this traditional approach was crucial particularly for descriptions early floras, such as the Dakota Formation that had few, if any, taxa that could be confidently assigned to modern genera (Dilcher, 2000). Linnaean binomial identifications of angiosperm taxa in this report are based on architectural, cuticular and other anatomic criteria for recognition of the leaf taxa, outlined in Upchurch and Dilcher (1990).

A second, more recent, approach supplementing the first approach is the morphotype system that provisionally establishes a morphotype tag or code for a distinct, identifiable plant organ, typically a leaf, which does not merit a Linnaean binomial name at the time first description (Ellis et al., 2009). The rationale for this system is absence of sufficient morphological features at the time of initial description of the fossil, for formal placement in a Linnaean genus and species (Peppe et al., 2008). The establishment of provisional morphotypes allows ecological analyses, including plant–arthropod interaction studies, of a known plant morphotype or an entire flora to occur before the work of determining a Linnaean binomial can be justified (Jud, 2014). A temporary establishment of a morphotype, such as New Genus A or Fern Type 2, allows comparison to similar specimens from the fossil record to establish a more rigorous taxonomic circumscription of the taxon in question (Peppe et al., 2008).

**Appendix S8: Criteria for recognizing herbivory from detritivory**

It is essential for a study that is centered on arthropod and pathogen herbivory of plants to establish evidence that distinguishes herbivory, or consumption of live tissue, from detritivory, the consumption of dead tissue. (We note that under the category of arthropod herbivory, we also include mites; for example, five of the 25 galls are attributable to mites.) Five categories of evidence are used to establish the presence of herbivory on fossil material.

**(a) Reaction tissue**

First, distinctive reaction tissue along a damaged surface is produced as a response to arthropod feeding. Examples include cut leaf edges (Mithöfer et al., 2005), plugs that seal punctures (Shackel et al., 1991; Walling, 2008), scars of oviposition lesions (Hamilton, 1980), and the thin reaction tissue of blotch leaf mines (Nieukerken et al., 2016). Pathogen damage from epiphyllous fungi, also produces reaction tissue. Pathogen reaction tissue consists of a reaction front, typically a broad zone, with a diffusion gradient extending outward from the initial fungal colonization site of the leaf surface that encompasses variously necrotic tissue of the fungal blotch (Agrios, 2005).

**(b) Stereotyped damage pattern**

A second criterion indicating herbivory is presence of a distinctive, stereotyped pattern of damage. For hole feeding, the damage consists of particular hole sizes, shapes and positions on a leaf consistent with a known feeding motif of an arthropod group or clade. An example is the leaf beetle *Phratora* *vitellinae* L. on willow, *Salix* *fragilis* L., in Eastern Europe (Urban, 2006b) that produces a highly distinctive skeletonization. Such patterns are determined by arthropod behavior, grazing levels, and plant response (Edwards and Wratten, 1983), inconsistent with abiotic damage such as tearing of leaves or fracturing of stems (Vincent, 1990; Clissold, 2007).

**(c) Microstructural features**

A third specification are microstructural features of plant damage associated with arthropod feeding patterns. For externally feeding arthropods, examples include sinistral or dextral patterns of leaf feeding (Bieńkowski, 2010a), small cuspules occurring along the broader cusp edge of a leaf excision (Gangwere, 1966), and strands of unconsumed veins along a leaf margin produced by a chewing insect (Clissold, 2007). For internal leaf feeding arthropods, examples are a stylet sheath surrounding a leaf puncture made by a piercing-and-sucking insect (Pollard, 1968) or mite, and a distinctive frass trail in a mine characteristic of a leaf-mining clade (Winkler et al., 2010). Gall formation provides noteworthy examples (Larew, 1981), as galled host-plant tissue represents control of the plant by the arthropod inducing the gall. A zone of hyperplasic tissue (enlarged cells) surrounding the gall results from the induction phase early in gall formation (Formiga et al., 2011) and hypertrophic tissue (cell multiplication) forms the walls during gall development (Álvarez et al., 2013).

**(d) Tissue, organ, or species specificity**

A fourth criterion is a pattern of damage resulting from targeting of a particular plant tissue or organ, or host-plant species, for consumption by a monophagous arthropod. An example is the distinctive foliar galls that occur on particular species of *Eucalyptus* (Myrtaceae) in Australia (Hardy and Gullan, 2010). Such a preferential consumption pattern is rarely accomplished by detritivorous arthropods that often target fungi but have typically eclectic feeding habits in consuming dead plant tissues that lack or have chemically denatured structural or secondary chemical defenses (Hanski, 1989). The inverse of this pattern is avoidance of antiherbivore plant structures such as leaf teeth (Keathley and Potter, 2011) or fibrous veins (Grubb et al., 2008).

**(e) Ecological uniformitarianism**

Fifth, a relevant issue for the recent part of the fossil record involves well-documented, modern patterns of herbivory from the entomological and agricultural literature. An example, reproduced with facsimile accuracy, occurs in Late Cretaceous and Cenozoic fossil plants. One case study is a gall-midge gall (Diptera: Cecidomyiidae) on ash *Fraxinus* *bilinica* (Unger) Z. Kvaček and Hurník (Oleaceae) from a lower Miocene (20 Ma) deposit in the Most Basin of the Czech Republic has the same structure as that produced by the modern gall midge *Dasineura* *fraxinea* Kieffer on *Fraxinus* *excelsior* L. (Knor et al., 2013). A second case study involving near-identical modern and fossil insect damage are the covers of armored scale insects on leaves of angiosperm hosts from middle Eocene (47–44 Ma) deposits in Germany, attributed to present-day Aspidiotinae scale insects (Hemiptera: Diaspididae) (Wappler and Ben-Dov, 2008).

**Appendix S9: Rarefaction analyses of the Rose Creek plant assemblage**

**(a)** Rarefaction–extrapolation analyses of specimen number vs surface area (cm^2^) for the Rose Creek plant assemblage and its major clades. Each clade has 1000 replications for resampling and 95% confidence intervals (Colwell et al., 2012; Hsieh et al., 2016).


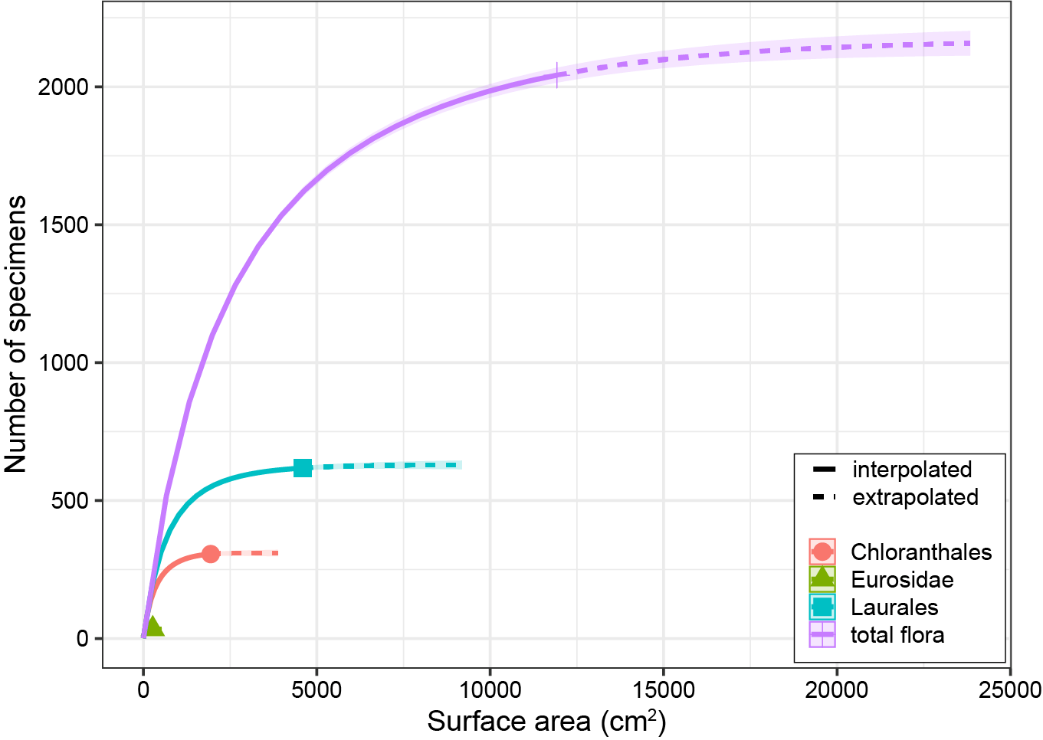


**(b)** Rarefaction–extrapolation analyses of specimen number vs surface area (cm^2^) for the Rose Creek plant assemblage and its major species. Nine most abundant species have 1000 repli-cations for resampling and 95% confidence intervals (Colwell et al., 2012; Hsieh et al., 2016).


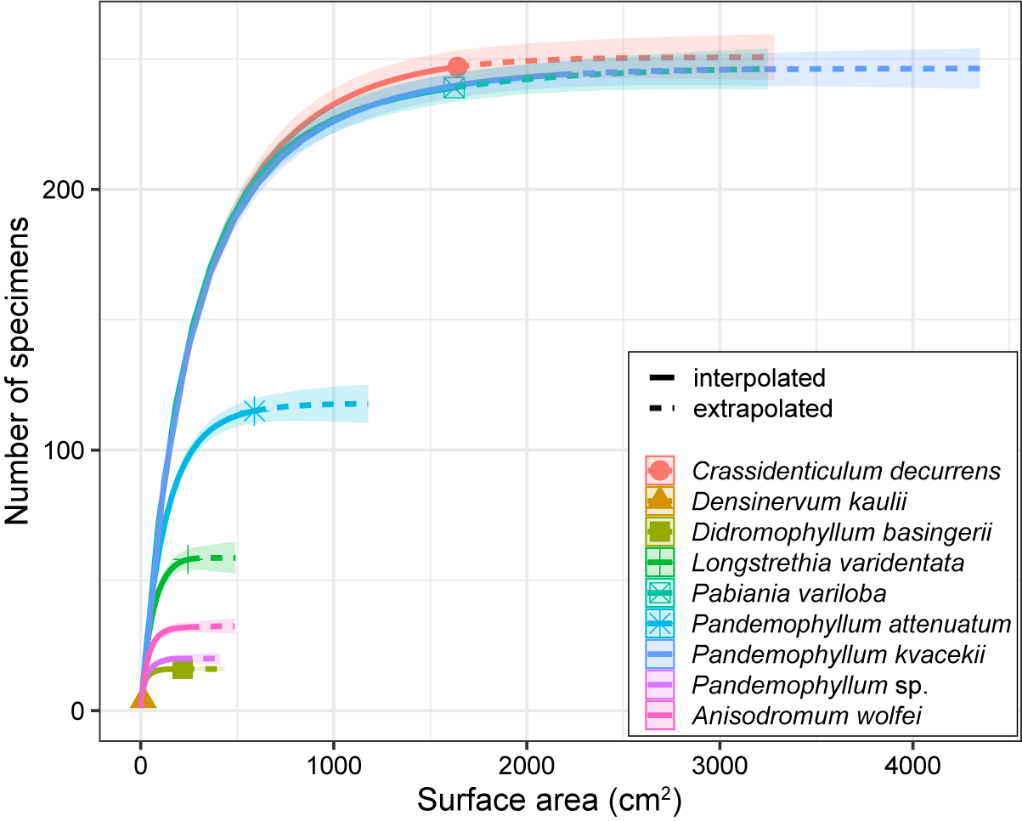


**(c)** Rarefaction–extrapolation analyses of damage types (DTs) vs surface area (cm^2^) for the Rose Creek plant assemblage and its major clades. Each clade has 1000 replications for resampling and 95% confidence intervals (Colwell et al., 2012; Hsieh et al., 2016).


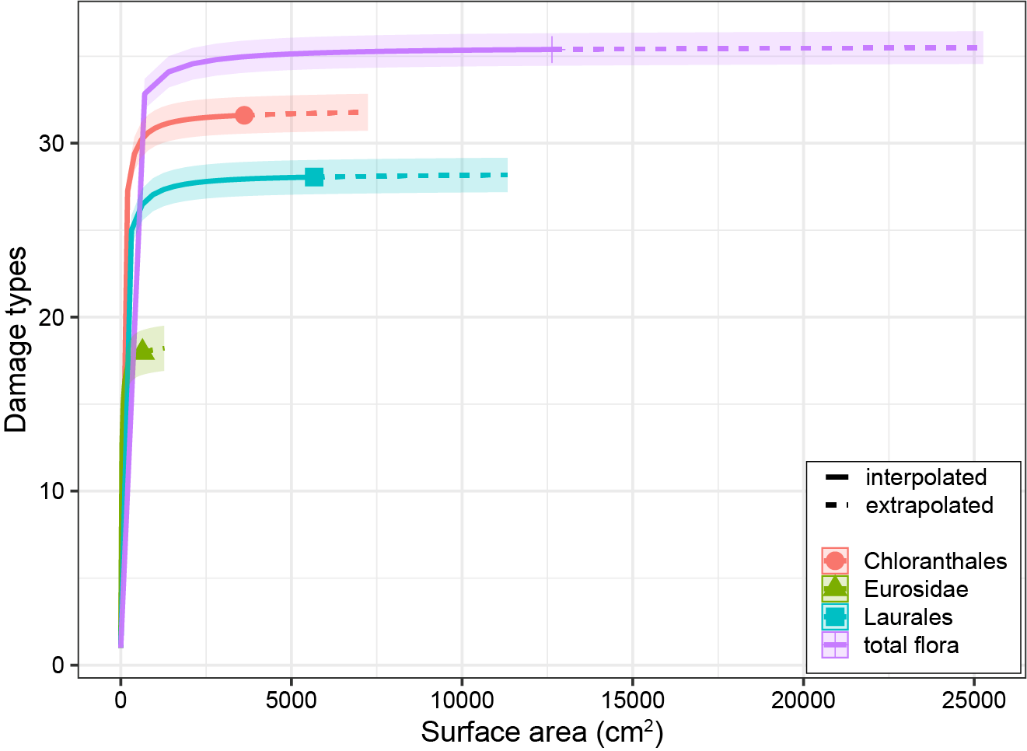


**(d)** Rarefaction–extrapolation analyses of damage types (DTs) vs surface area (cm^2^) for the Rose Creek plant assemblage and its major species. Nine most abundant species have 1000 repli-cations for resampling and 95% confidence intervals (Colwell et al., 2012; Hsieh et al., 2016).


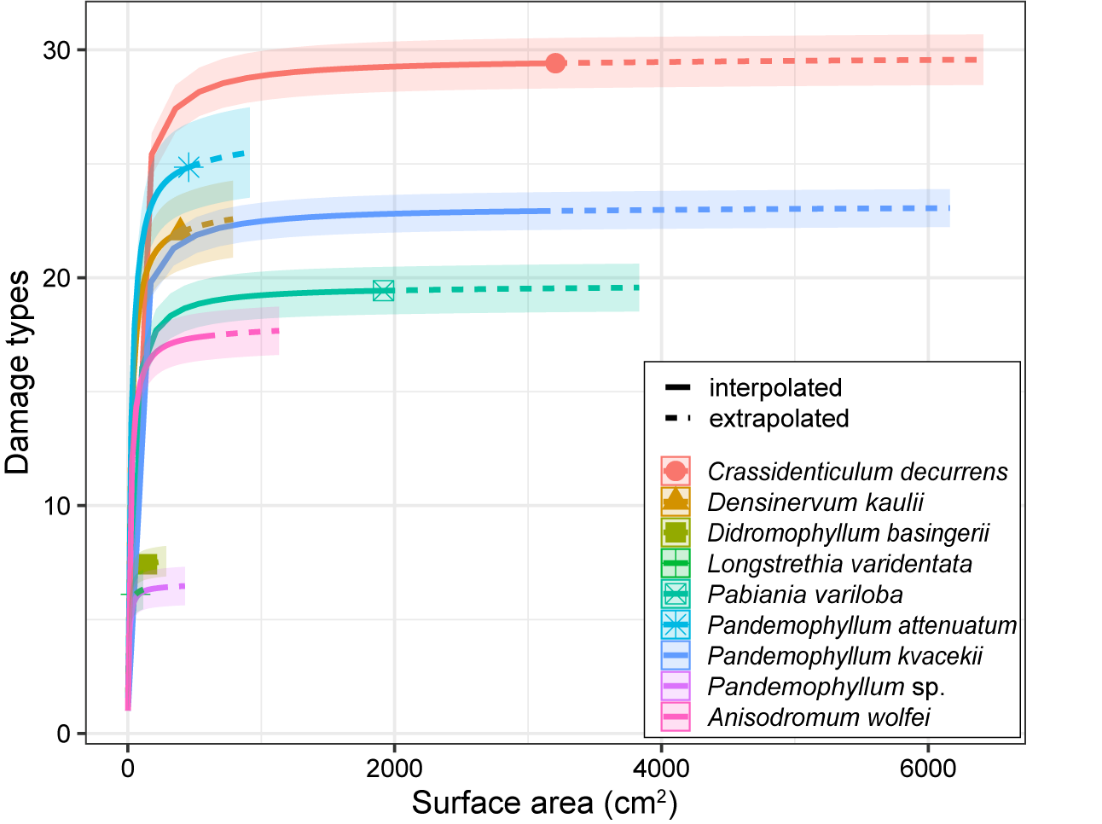


**Appendix S10: Evaluation of Rose Creek florivory**

Florivory consists of herbivory of flowers, particularly their petals of (Frame, 2003) that are typically consumed by insect pollinators (McCall and Irwin, 2006). The Rose Creek plant assemblage produced nine flower and reproductive axis morphotypes that consisted of *Dakotanthus* *cordiformis*, known as the Rose Creek Flower (Basinger and Dilcher, 1984; Manchester et al., 2018); two other unnamed flower morphotypes; and six reproductive axis morphotypes. Of these nine taxa/morphotypes, only one flower type showed examples of florivory. *Dakotanthus* *cordiformis* exhibited hole feeding of DT1, DT2 (Leavitt and Robinson, 2006; Bieńkowski, 2010b), margin feeding of DT405 (Koeniger et al., 2017), and piercing and sucking of DT402 (Childers and Achor, 1991, Xiao et al., 2010b). These data are analyzed in a study that examines florivory across the several major localities of the Dakota Formation (Xiao et al., 2021a).

**Appendix S11: Functional feeding groups (FFGs) and feeding classes**

The eleven FFGs detailed in this report are divided into four broader feeding classes. First, ectophytic consumers are external feeders in which the arthropod is located external to the plant host and consists of the hole feeding, margin feeding, skeletonization and surface feeding FFGs. Second, is ectoendophytic consumers in which the ovipositor or mouthparts is located internally within host plant tissues, but the body is externally positioned, and consists of the oviposition and piercing-and-sucking FFGs. Third is endophytic consumers that are internal feeders in which the arthropod is located within the host’s tissues, and consists mining, galling, seed predation, and borings FFGs. A fourth feeding class consists of pathogens, that consume plants by absorption or diffusion of nutrients through an advancing mycelial or bacterial front. The extensive development of pathogen damage of the Rose Creek plant assemblage has resulted in half (7/14) of the newly described DTs that previously have not been assessed in plant–arthropod interaction studies, although informal identifications have been made (Dilcher, 1974; Labandeira and Prevec, 2014). We include oviposition as an FFG because it represents a distinctive type of damage facilitated by a piercing or slicing insect ovipositor that penetrates plant tissues, similar to piercing and sucking, and has a well-represented fossil record (Beamer, 1928).

**Appendix S12:** Raw data of plant clades/species of feeding class, functional feeding group, damage type, host specificity, and feeding event occurrences


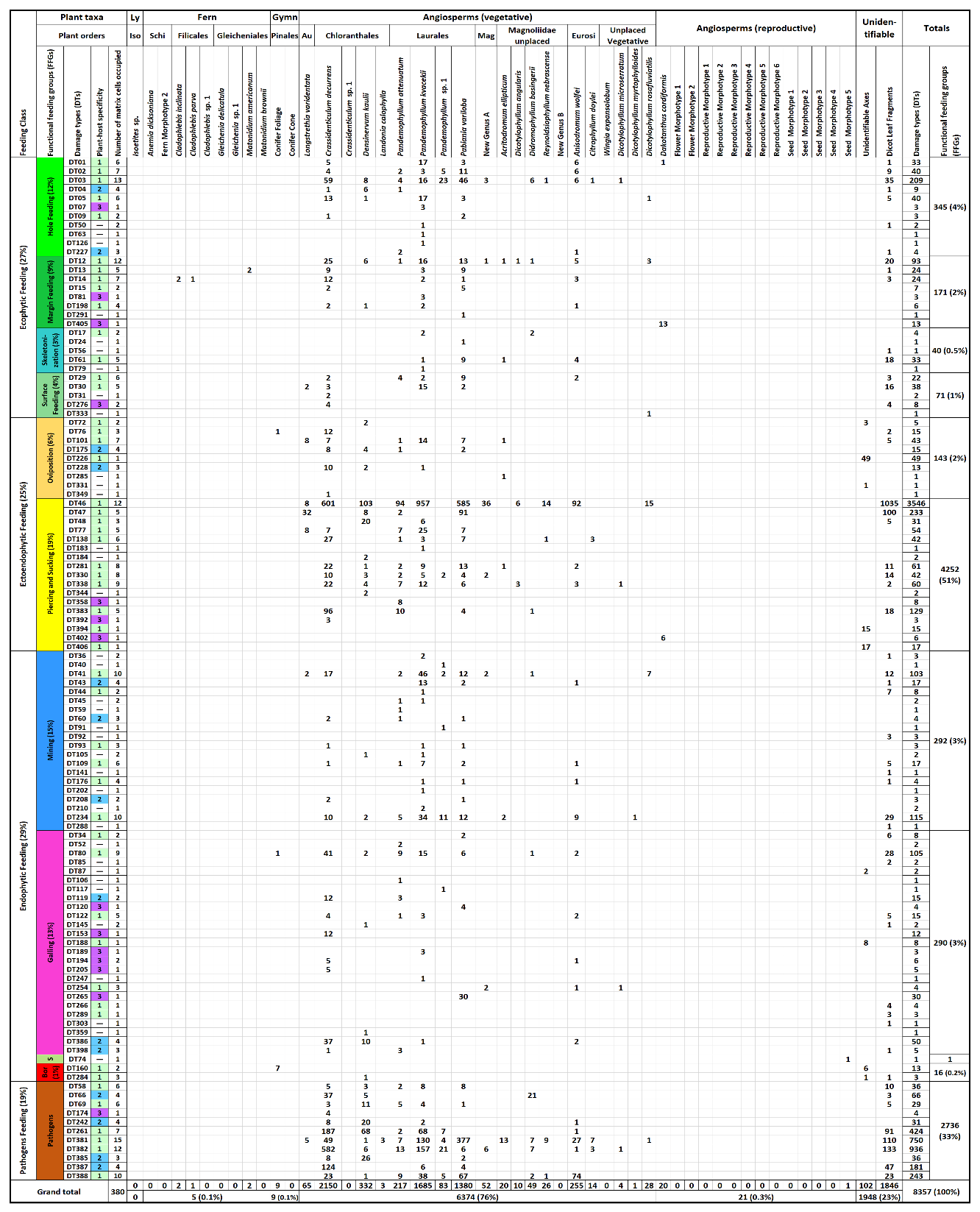


Abbreviations: Au, Austrobaileyales; Bor, Borings; Eurosi, Eurosidae; Gymn, Gymnosperms; Iso, Isoetales; Ly, Lycopoda; Mag, Magnoliidae; S, Seed Predation; Schi, Schizaeales.

**Appendix S13:** **Figs. S1–S15.** **Continuation of photographic documentation of herbivory**

**
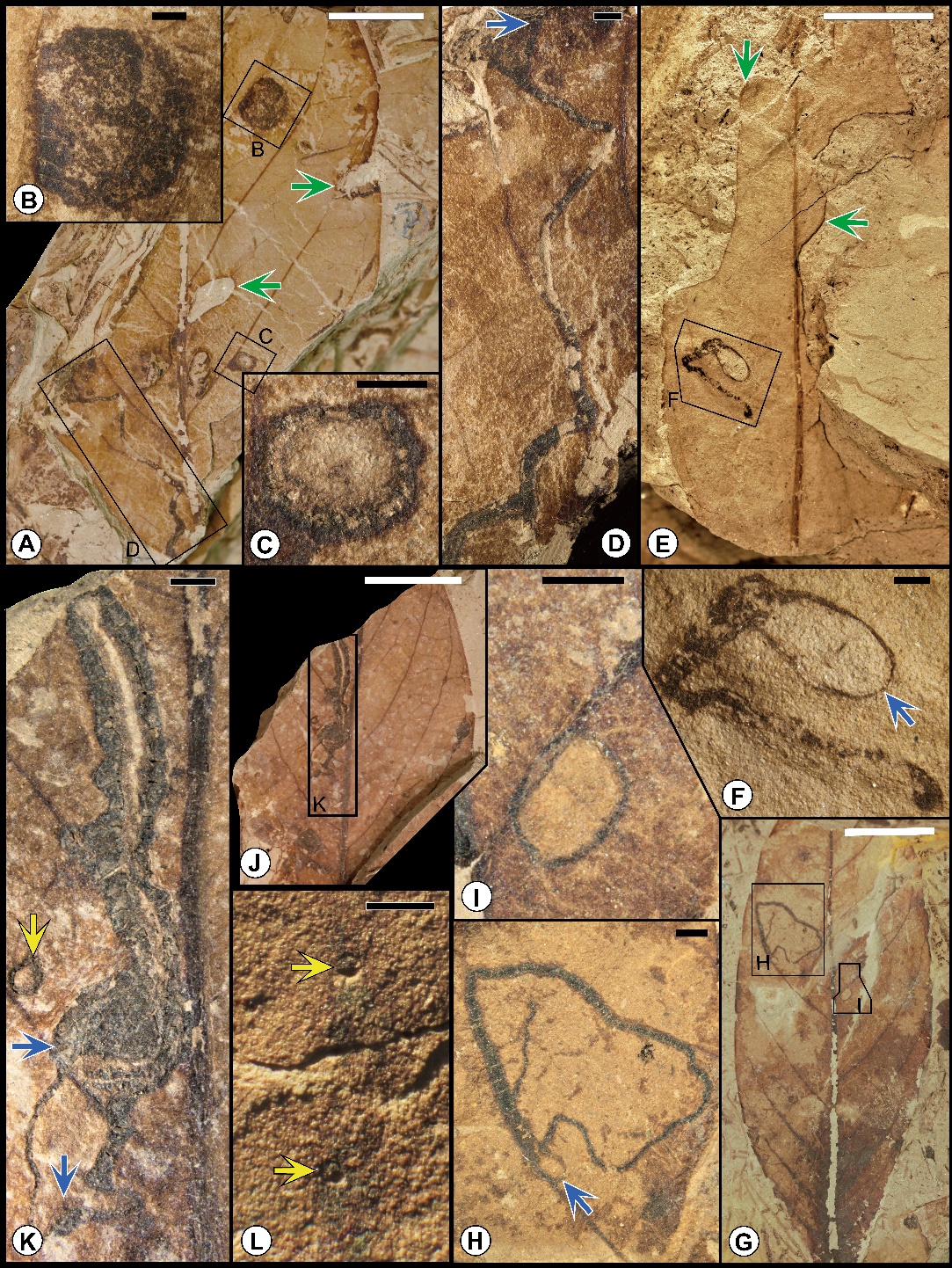
**

**(a) Fig. S1**. The

Component herbivore community of *Pandemophyllum* *kvacekii* I, showing six FFGs of hole feeding, margin feeding, surface feeding, piercing and sucking, mining, and pathogen damage. Specimen UF-16137-1 at (**A**), consisting of hole and trenched margin feeding DT15 (green arrows) and the pathogen necroses of DT388 enlarged in (**B**), DT261 enlarged in (**C**) with a central fructification, and an incomplete DT234 mine displaying a triangular oviposition lesion (blue arrow) in (**D**). A small leaf mine of DT176 occurs on UF-16132 at (**E**), enlarged in (**F**), has an outsized terminal chamber (blue arrow) in a leaf that also has extensive DT12 (left green arrow) and DT14 (right green arrow) margin feeding. At (**G**) is UF-12723 that contains the mine DT41, magnified in (**H**), showing control by major veins and a bulbous terminal chamber (blue arrow); also present is DT30 surface feeding, magnified in (**I**). UF-16142-1 in (**J**) shows a necrosis of the pathogen DT381(not show) and a complete mine of DT234, enlarged in (**K**), that displays the initial oviposition site (lower blue arrow) and ovate terminal chamber (upper blue arrow); also present is piercing-and-sucking DT46 (yellow arrow) with thick reaction tissue, and DT138 adjacent to small DT1 hole feeding (not displayed here). UF-16148 at (**L**) is a DT392 example of piercing and sucking along the major vein. Scale bars: white, 10 mm; black, 1 mm. See Figs. S2–S4 in the online supplementary data that continues documentation of the *Pandemophyllum kvacekii* herbivore component community. Scale bars: white, 10 mm; black, 1 mm.


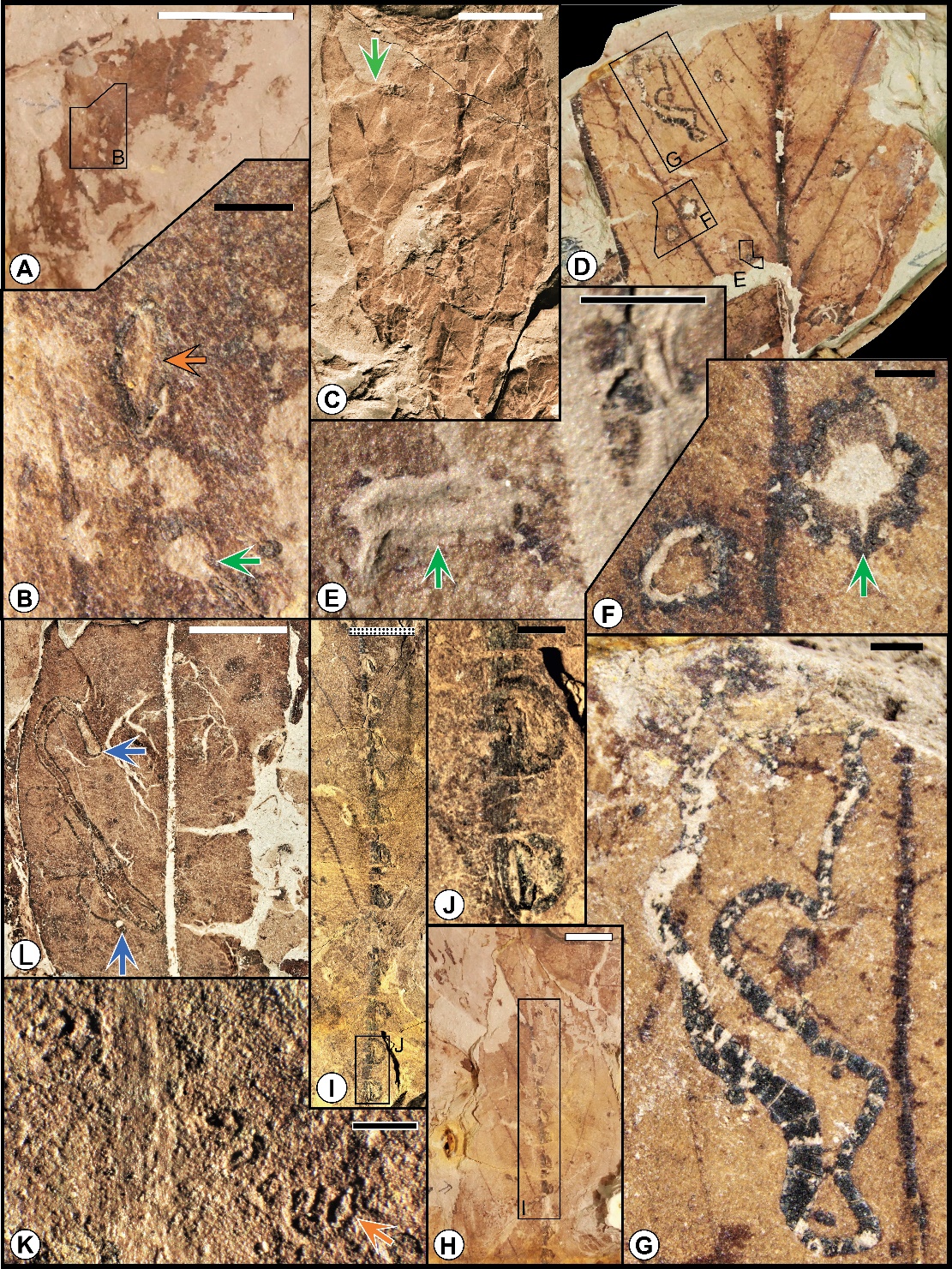


**(b) Fig. S2.** The component herbivore community of *Pandemophyllum* *kvacekii* II, displaying five FFGs of hole feeding, margin feeding, surface feeding, oviposition, and mining. Fragmentary specimen UF-16221-4 in (**A**) shows DT101 oviposition (orange arrow) marks aligned parallel to secondary veins, enlarged in (**B**) and poorly preserved DT227 hole feeding (green arrow). UF-4884 in (**C**) shows an example of DT12 (green arrow). At (**D**) is UF-16144-1 that has DT103 surface feeding enlarged in (**E**) that displays a robust reaction rim (green arrow), two DT2 hole feeding areas with a widened outer wall enlarged in (**F**), an incomplete DT234 leaf mine, enlarged in (**G**). UF-16221-3 at (**H**) shows a series of eight, ovate, DT228 oviposition lesions, magnified in (**I**), two of which are further magnified in (**J**) showing flared lesion scars. At (**K**) is UF-204001, consisting of a series of 11 DT101 oviposition lesions, oriented oblique to the midvein (orange arrow). UF-16098-1 at (**L**) consists of a complete DT234 mine with initial oviposition lesion (bottom blue arrow) and terminal chamber (top blue arrow) that co-occurs with DT3 hole feeding (not shown). Scale bars: white, 10 mm; striped, 5 mm; black, 1 mm.


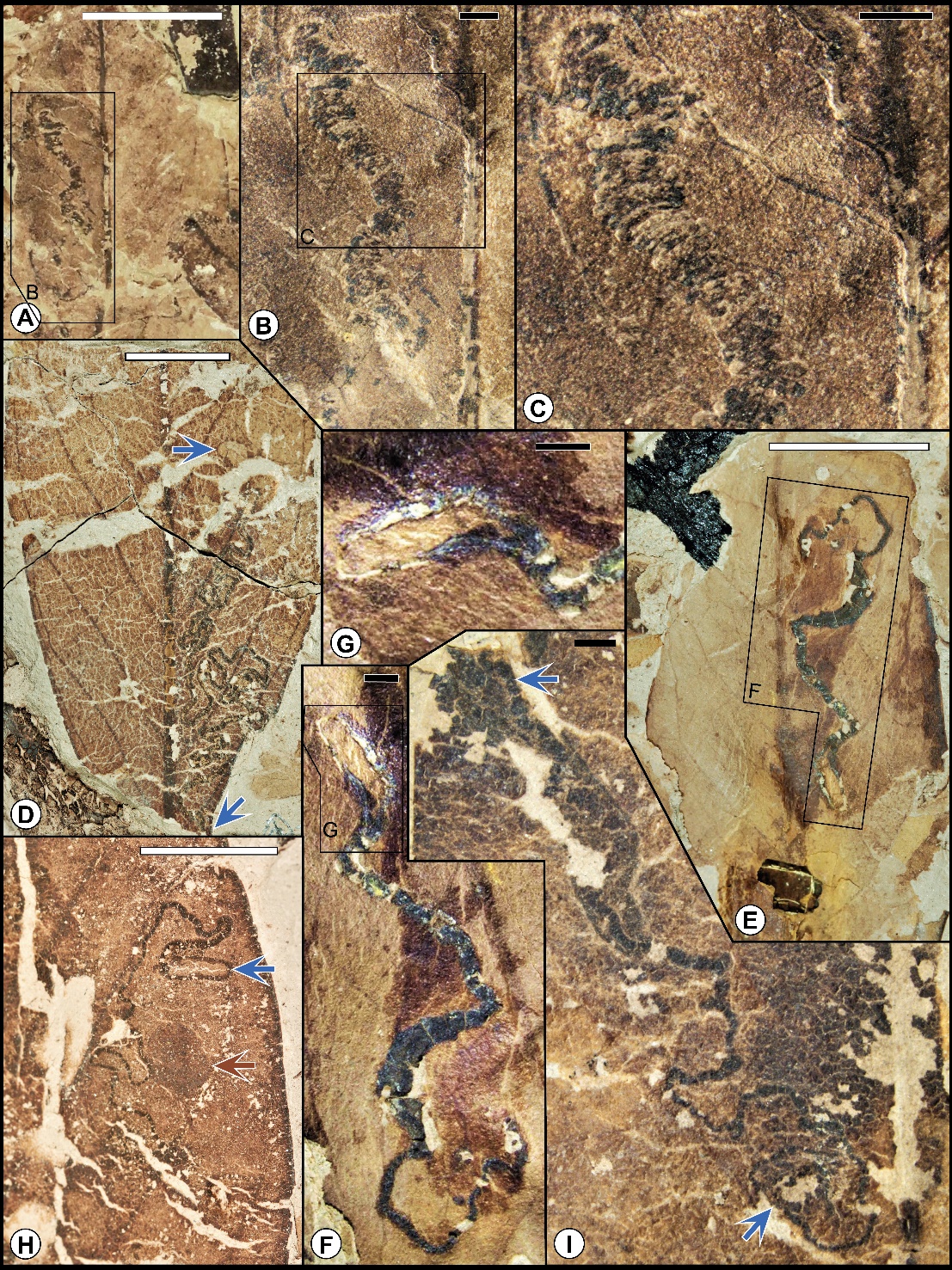


**(c) Fig. S3.** The component herbivore community of *Pandemophyllum* *kvacekii* III, illustrating two FFGs of mining and pathogen damage. UF-16160-1 at (**A**) with (not shown) DT46 piercing and sucking, a DT210 mine, and displayed DT93 mine showing an extensive, tightly sinusoidal, frass trail enlarged in (**B**) further enlarged in (**C**) to provide details of the frass looping. UF-16140 at (**D**) consists of a complete DT234 mine at the right side of the leaf that displays an oviposition site (bottom blue arrow), extensive looping of the frass trail and a terminal chamber (top blue arrow). UF-12722-8 at (**E**) is a DT45 leaf mine, enlarged in (**F**) and earlier frass trail enlarged at (**G**). This mine occurs with curvilinear rows of DT338 punctures (not displayed). UF-16098-2 at (**H**) with a highly sinuous DT41 mine at the right side of the leaf that has an elongate-elliptical terminal chamber (blue arrow) that is adjacent to a DT381 pathogen necrosis (brown arrow). UF-16109-1 at (**I**) illustrating a DT41 mine that has an oviposition area (lower blue arrow), a mine containing solid frass, and a terminal chamber (upper blue arrow). Scale bars: white, 10 mm; black, 1 mm.


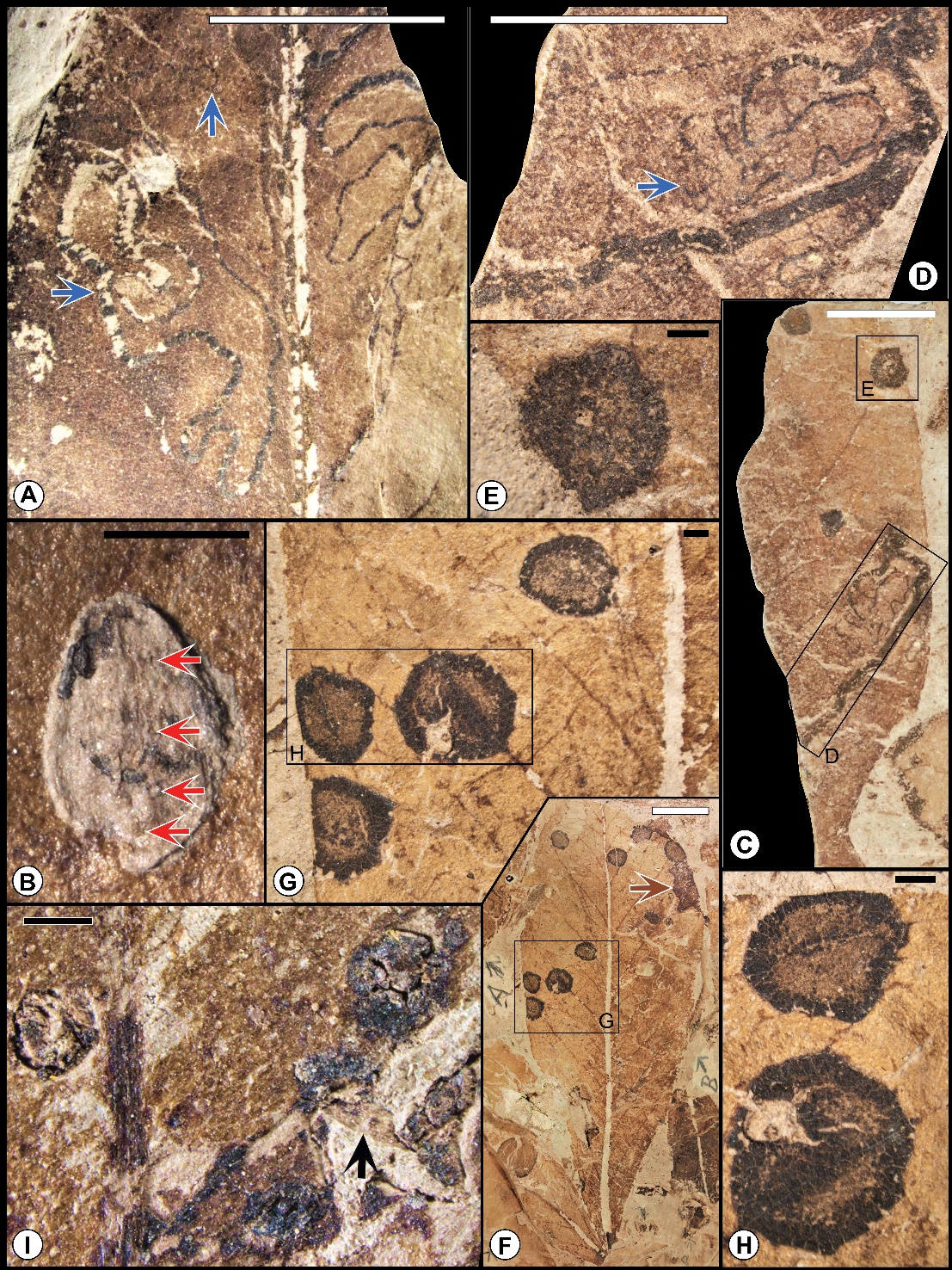


**(d) Fig. S4.** The component herbivore community of *Pandemophyllum* *kvacekii* IV, with examples of four FFGs of piercing and sucking, mining, seed predation, and pathogen damage. Specimen UF-12712 at (**A**), exhibiting one complete DT41 mine at left with an oviposition site (upper blue arrow) and circular terminal chamber (lower blue arrow), and two DT41 mines at right, together with (not shown) DT388 pathogen and DT58 pathogen necroses. Seed type 5, occurring on the leaf surface of a *Pandemophyllum*, shows four punctures (red arrows) of DT74 seed predation at (**B**). UF-16145-1 at (**C**) is a complete DT234 mine with an oviposition site (top blue arrow) and terminal chamber (bottom blue arrow) enlarged in (**D**), and pathogen necroses of DT388 highlighted in (**E**), showing a centrally undefined area surrounded by a mycelial mat with a diffuse reaction front. UF-16123-2 at (**F**) contains one example of DT381 at upper right (brown arrow), and about ten examples of DT388, four of which are enlarged in (**G**), and two of which are further enlarged at (**H**). UF-16098 at (**I**) shows a DT77 piercing-and-sucking scale mark at bottom, and two DT388 necroses at the top (not shown). The structure indicated by the black arrow is unknown. Scale bars: white, 10 mm; black, 1 mm.


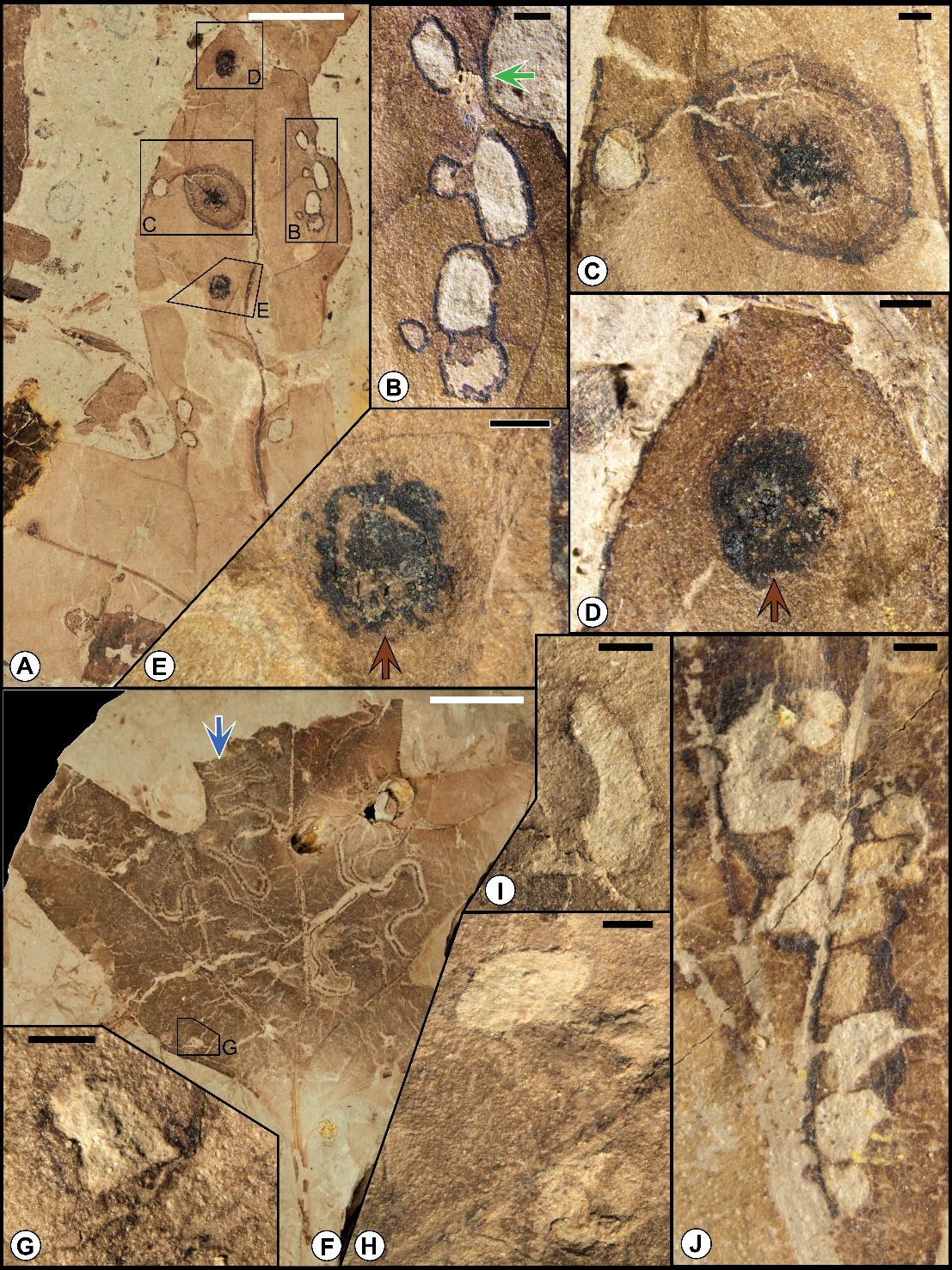


**(e) Fig. S5.** The component herbivore community of *Pabiania variloba* I, showing five FFGs of hole feeding, margin feeding, skeletonization, mining, and pathogen damage. At (**A**) is UF-16191, containing piercing-and-sucking DT46 (not shown), hole feeding (DT1, DT3) and margin feeding (DT12) enlarged at (**B**), and fungal necroses of DT387 with degraded central fructifications enlarged at (**C**), (**D**) and (**E**) that display an inner central zone of fructifications that are often displaced to the mycelial edge (brown arrows) outer reaction fronts. The thallus at (D) may have an area of fungivore consumption indicated by a brown arrow. At (**F**) is UF-12708p/c that has two DT234 mines, the left one indicated by the blue arrow, and extensive DT3 hole feeding indicated at (**G**), (**H**) and (**I**). UF-16218 at (**J**) is an example of skeletonization DT61 present along a major vein. Scale bars: white, 10 mm; black, 1 mm. See Figs. S6–S8 in the online supplementary data that continues documentation of the *Pabiania* *variloba* herbivore component community. Scale bars: white, 10 mm; black, 1 mm.


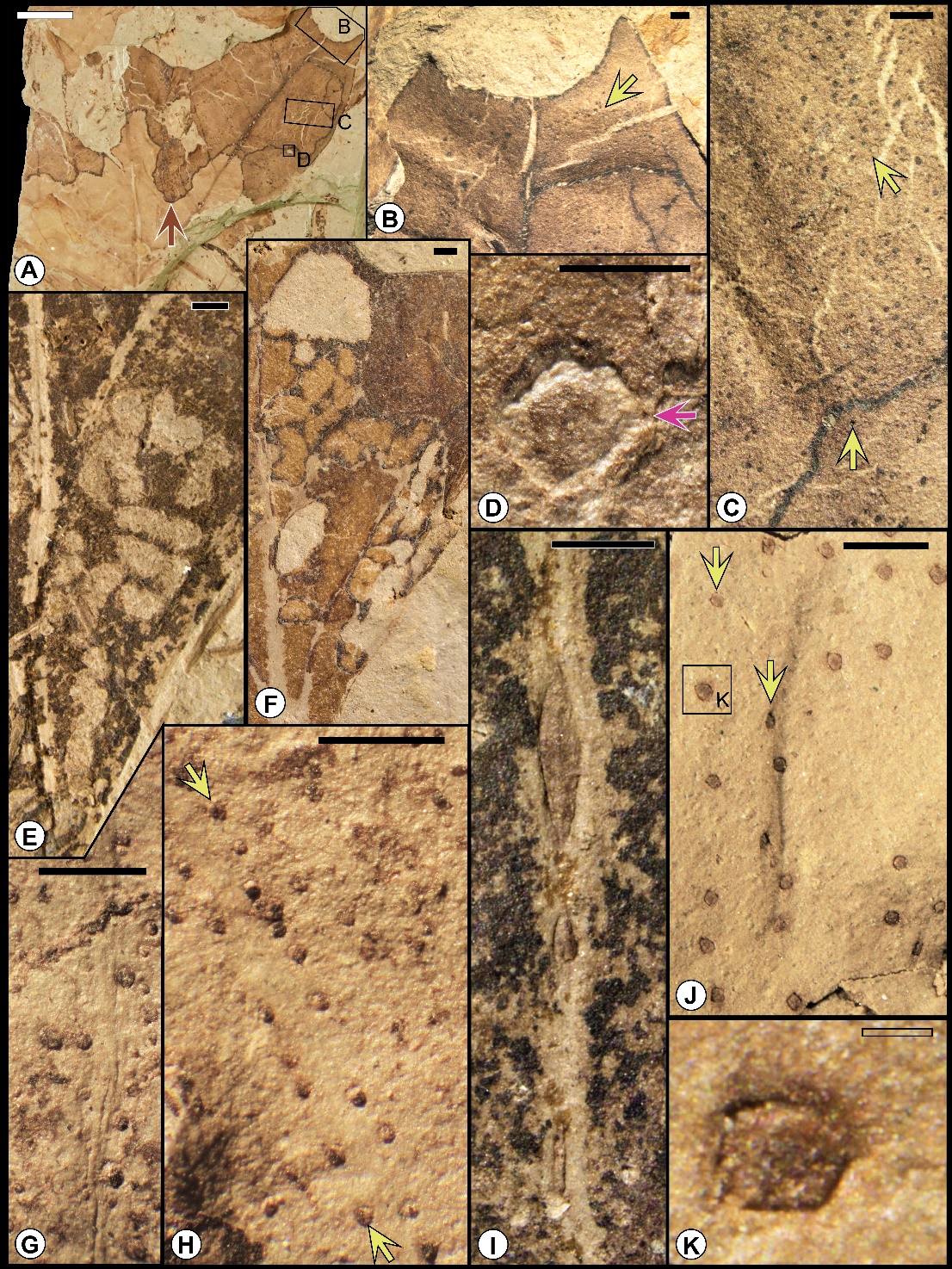


**(f) Fig. S6.** The component herbivore community of *Pabiania* *variloba* II, showing seven FFGs of hole feeding, margin feeding, skeletonization, oviposition, piercing and sucking, galling, and pathogen damage. UF-12713 at (**A**) displays a DT385 pathogen necrosis, with the brown arrow delimiting a reaction front, and DT13 margin feeding and DT46 piercing and sucking (yellow arrow) occurring at the leaf-lobe apex in (**B**); part of randomly distributed DT46 and curvilinear rows of DT338 (yellow arrows) are present in (**C**), of which one cratered gall DT247 is in (**D**). UF-12697 at (**E**) shows the stereotyped pattern of DT9 hole feeding. UF-16215 at (**F**) consists of DT381 necroses, DT2, DT3 and DT5 hole feeding indicated by the presence of underlying matrix, and examples of DT61 skeletonization indicated by a lighter brown hue than that of the unherbivorized leaf. UF-3323 at (**G**) are scattered DT47 punctures, DT138 along the vertical vein, and multiple linear rows of DT338 that are enlarged in (**H**), indicated by yellow arrows. Also, at UF-12697 from specimen (E) is oviposition DT175 at (**I**), displaying lesions that are serially arranged along a major vein. At UF-3323 at (**J**) are linear rows of DT138 punctures along the sides of major veins, and DT338 curvilinear rows of punctures in non-veined regions of the blade (yellow arrows) amid scattered DT46 punctures, enlarged in (**K**). Scale bars: white, 10 mm; black, 1 mm; empty, 0.5 mm.


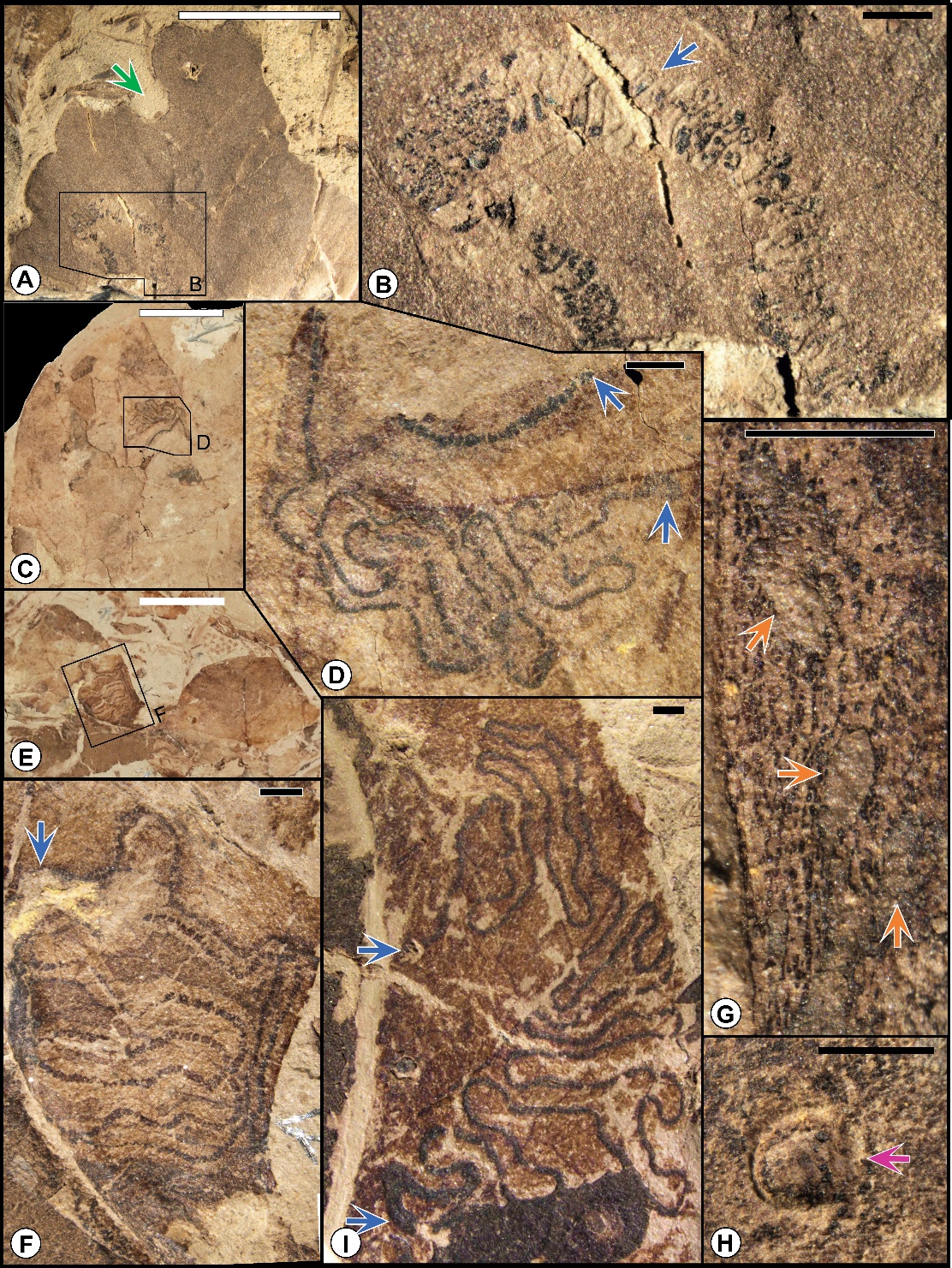


**(g) Fig. S7.** The component herbivore community of *Pabiania* *variloba* III, displaying five FFGs of margin feeding, oviposition, mining, galling and pathogen damage. Specimen UF-12684 at (**A**) exhibiting DT12 margin feeding (green arrow) and a DT93 mine at bottom-left highly enlarged in (**B**), showing a tig sinusoidal, medial frass trail and leaf mine border (blue arrow). At (**C**) in UF-16166-2 is a highly winding DT41 leaf mine, magnified in (**D**) and showing a widening of the mine at the blue arrow. At (**E**), UF-16195 displays a highly winding DT41 leaf mine, magnified in (**F**) that is circumscribed by primary veins but not secondary veins and shows a terminal chamber bereft of frass (blue arrow). UF-16195 at (**G**) shows three DT76 oviposition lesions on or alongside a major vein (orange arrows), and a small DT303 gall enlarged at (**H**) displaying a thick gall wall (magenta arrow). UF16173-1 at (**I**) displays a highly convoluted DT41 mine, alongside (not illustrated) DT381 pathogen necrosis at bottom and small (not illustrated) DT80 galls. Scale bars: white, 10 mm; black, 1 mm.


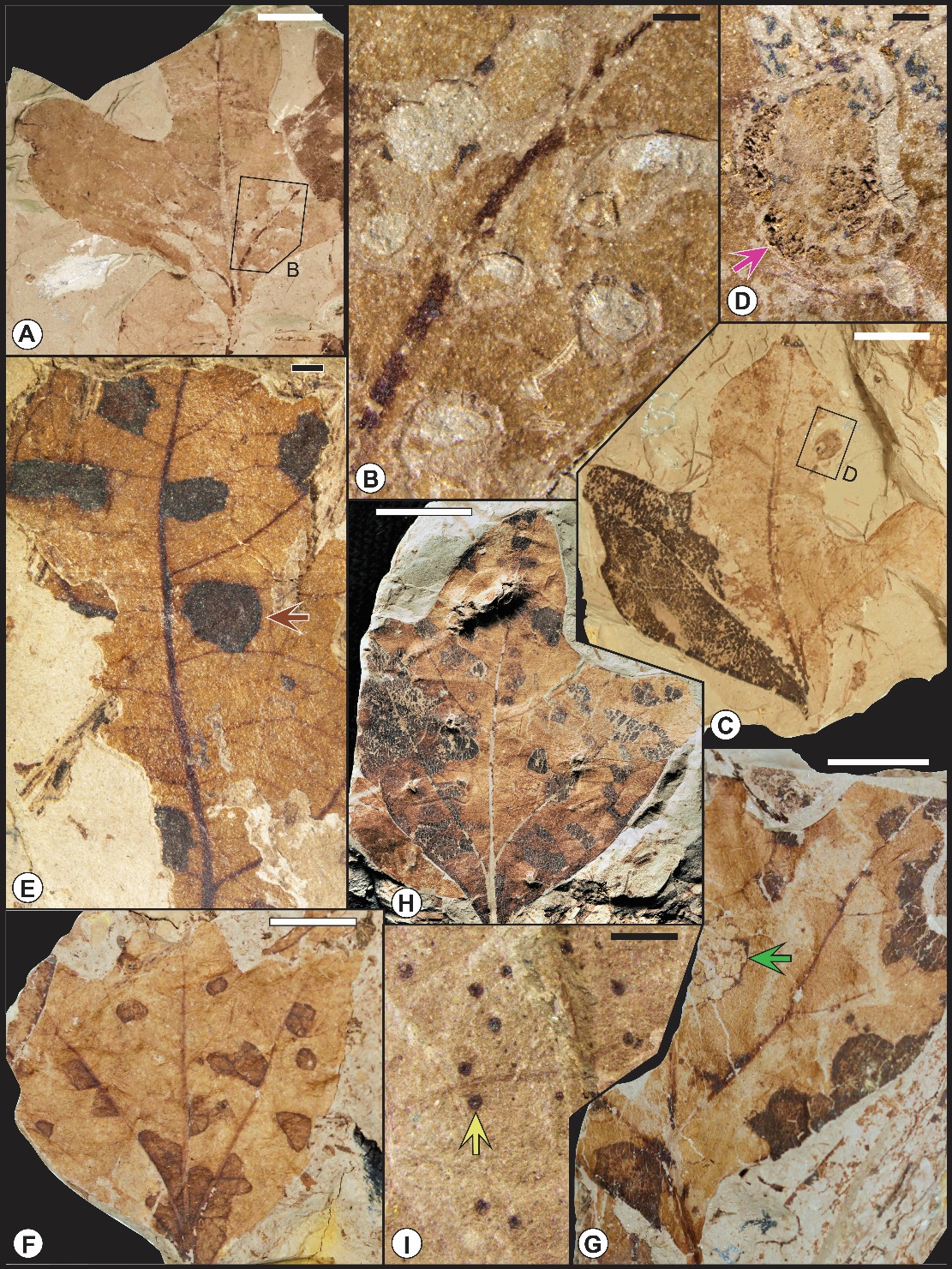


**(h) Figure S8.** The component herbivore community of *Pabiania* *variloba* IV, illustrating four FFGs of skeletonization, piercing and sucking, galling, and pathogen damage. Specimen UF-16166 at (**A**), showing a cluster of loosely aggregated DT265 galls occurring at the leaf base, enlarged in (**B**) and present in a field of pervasive DT330 piercing-and-sucking damage (not shown). UF-12710 at (**C**) shows a DT120 gall, enlarged considerably in (**D**), showing a sclerified outer wall (magenta arrow). Specimens from (E) to (H) show the pervasiveness of DT381 pathogenic damage. At (**E**) is UF-16101-10, showing about nine examples of irregularly shaped DT381 necroses, each with a reaction front (brown arrow). UF-16206 at (**F**) displays a second example of several, polygon necroses of the DT381 pathogen. A third example of DT381 pathogen necroses occurs along the leaf margin of UF-16178 at (**G**), and the DT24 skeletonization near the major vein (green arrow) of UF-16178 at (**G**). A fourth example of DT381 is present in UF-7835 at (**H**), which shows various shaped necroses present along the outer perimeter of the leaf. At (**I**) is UF-12648, displaying DT46 punctures (yellow arrow). Scale bars: white, 10 mm; black, 1 mm.


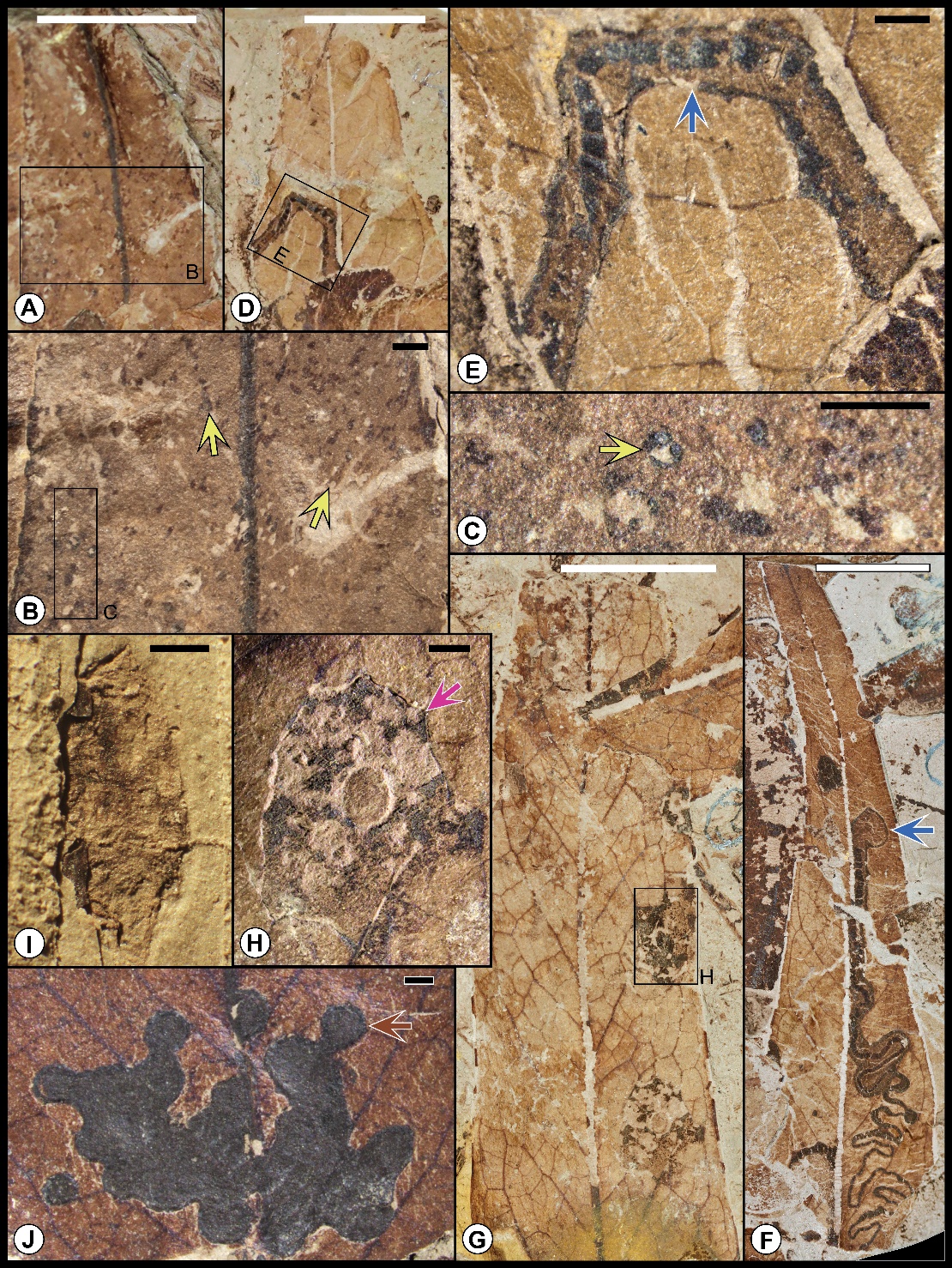


**(i) Fig. S9.** The component herbivore community of *Pandemophyllum attenuatum*, with examples of four FFGs of piercing and sucking, mining, galling, and pathogens. Specimen UF-16117 at (**A**), displaying three DTs of piercing and sucking, enlarged in (**B**) that includes randomly positioned DT46 punctures, a linear row of punctures along a major vein for DT138 (yellow arrow at right), a curvilinear row of punctures between major veins for DT338 (yellow arrow at left), enlarged at (**C**). UF-16135-2 at (**D**) is a mine fragment made by a late instar of the DT234 leaf miner, enlarged in (**E**), with the mine wall and reaction tissue indicated (blue arrow). At (**F**) is UF-16196-1 with a near-complete DT234 mine with a terminal chamber (blue arrow). This leaf also has surface feeding (DT29), piercing and sucking (DT46), galling (DT80), and pathogens (DT381, DT388) (not shown). (**G**) in UF-12722-1, showing DT3 hole feeding, DT46 piercing and sucking (both inconspicuous) and the distinctive gall DT398 with wispy extensions into adjacent veins, enlarged in (**H**). The magenta arrow points to the gall wall and a wispy extension into a vein. At (**I**) is UF-4802 is the ovoidal petiolar gall DT122, with DT2 hole feeding and DT80 galling present on the same leaf (not shown). At (**J**) is a mature, multilobed necrosis of DT388 originating from a leaf base, showing colonization of the leaf surface by about nine, advancing mycelial extensions. One of these mycelial fronts is shown by a brown arrow. Scale bars: white, 10 mm; black, 1 mm.


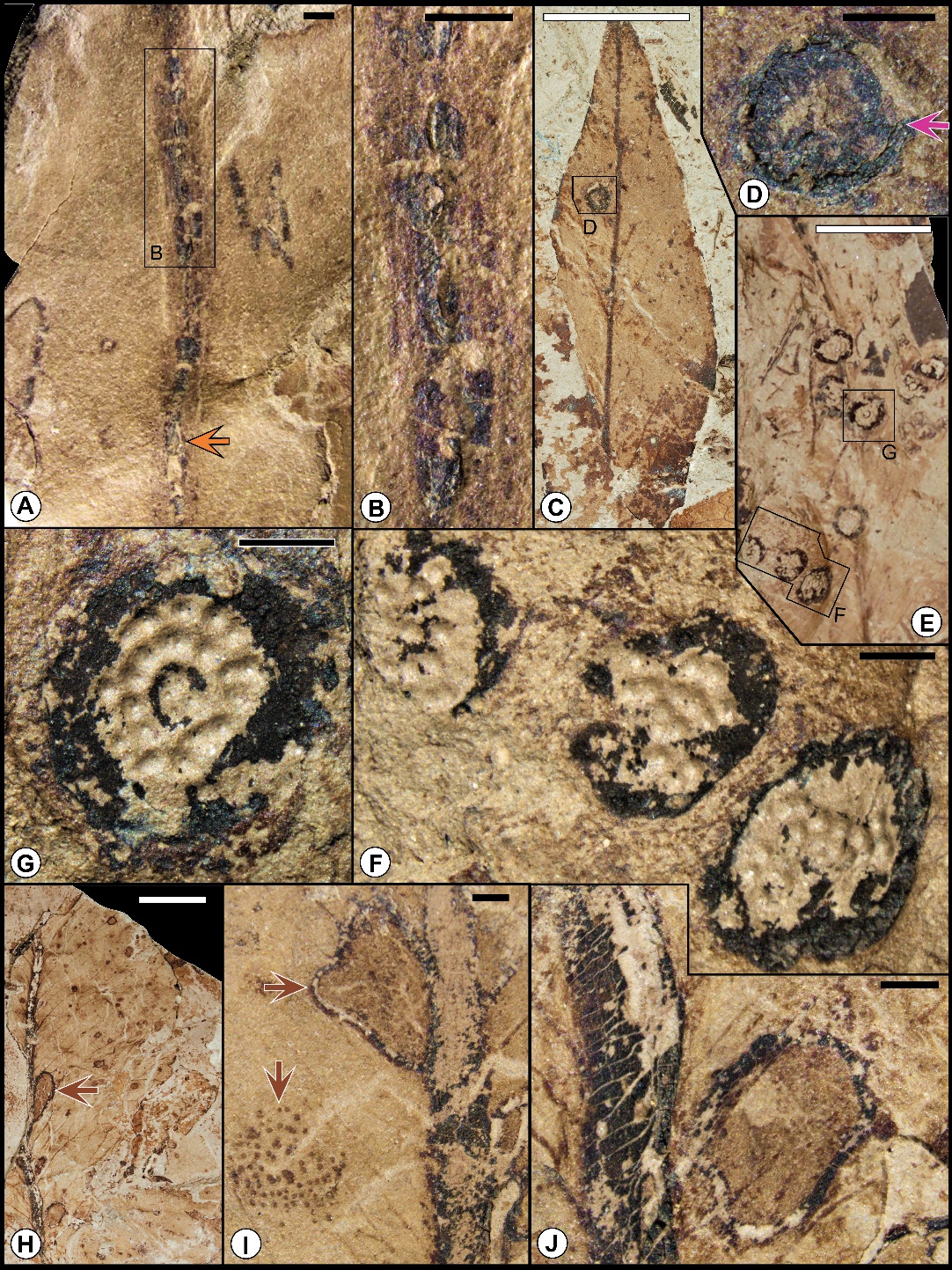


**(j) Fig. S10.** The component herbivore community of *Densinervum* *kaulii*, showing four FFGs of oviposition, mining, galling, and pathogen damage. Specimen UF-16141-2 at (**A**), contains a poorly preserved DT234 mine, DT76 oviposition (orange arrow), and DT228 representing serial oviposition on the midrib, enlarged in (**B**). At (**C**) is UF-12722-1 containing a hardened DT359 gall with a thick wall and small central chamber, enlarged in (**D**). At (**E**) is UF-16131, that has the distinctive “golf ball gall” of DT386, ornamented by rings of shallow hemispheroidal bumps; a row of three DT386 galls is enlarged in (**F**), and a well-preserved specimen with a central black ring is enlarged in (**G**). At (**H**) and (**I**) is UF-16105, a leaf consisting of necroses of the three pathogens of DT58 (brown arrow), DT69 enlarged in (**J**) and indicated by the upper brown arrow in (**I**), and DT66 that occurs at the lower-left of (I), designated by the lower brown arrow. (Part (I) is the base of the leaf that is not figured in part (J).) Scale bars: white, 10 mm; black, 1 mm.


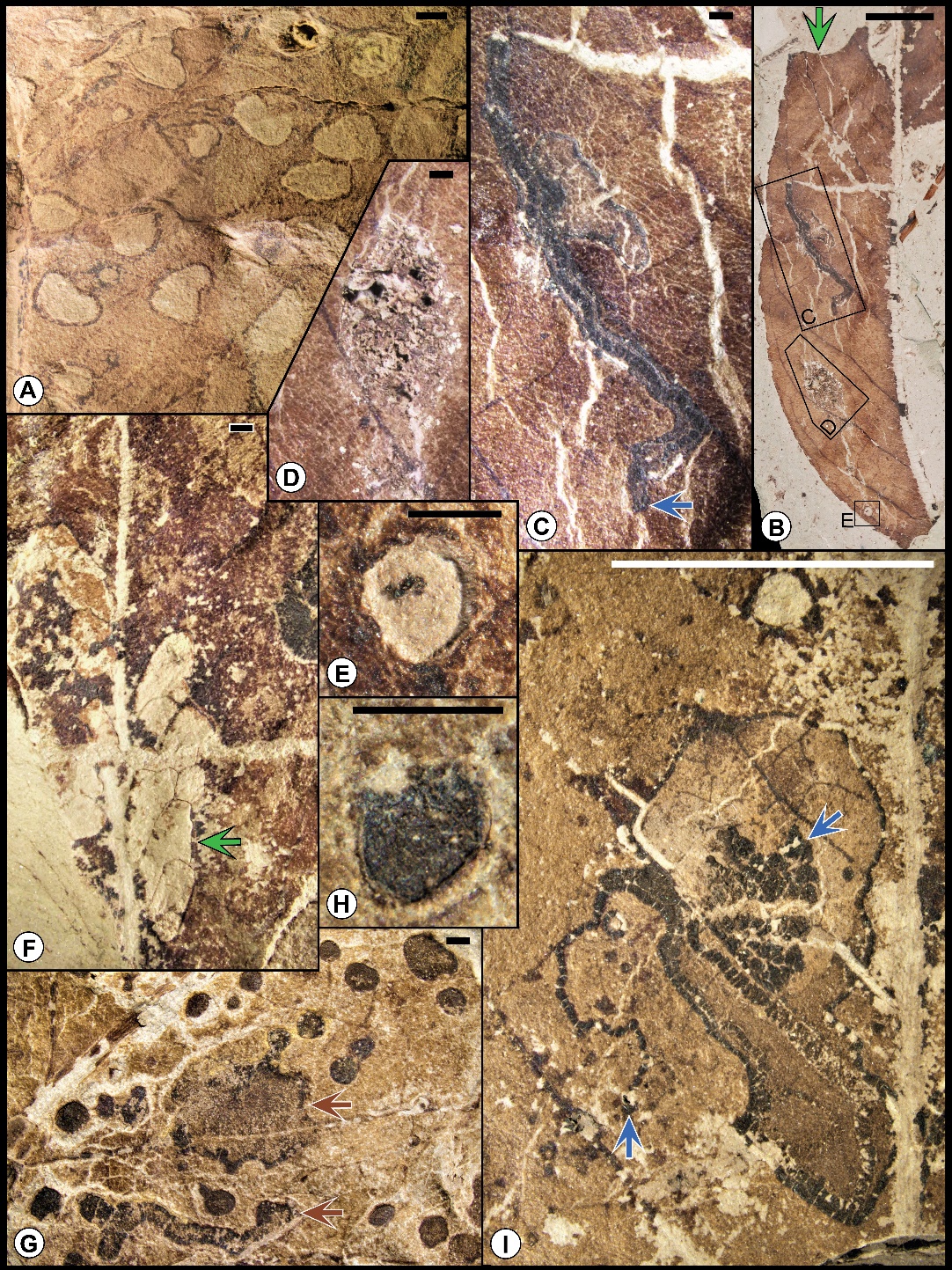


**(k) Fig. S11**. The component herbivore community of *Anisodromum* *wolfei*, displaying six FFGs of hole feeding, margin feeding, skeletonization, mining, galling, and pathogen damage. Specimen UF-7884-2 at (**A**) consists of extensive hole feeding of DT3 with some interspersed DT1 holes. At (**B**) is UF-16198-1, displaying a complete DT234 mine, with the terminal chamber enlarged in (**C**) and indicated by a blue arrow, a DT275 gall consisting of several internal, circular structures, enlarged in (**D**), and DT3 hole feeding enlarged in (**E**). (The possibility remains that (D) is a coprolite.) At (**F**) is UF-16193-1, showing skeletonization (green arrow), accompanied by diffuse necroses of pathogen DT388. At (**G**) is UF-5401-1 has circular to ovoidal to polylobate necroses of pathogen DT388, one of which is enlarged in (**H**), and more irregularly to serpentine shaped forms of pathogen DT381 (brown arrows). This leaf also contains DT48 piercing and sucking and a DT43 mine that are not displayed. UF-12724 at (**I**) consists of DT2 hole feeding at the top, DT46 and DT138 piercing and sucking (not shown), and a DT234 mine whose oviposition site (lower blue arrow) and terminal chamber (upper blue arrow) are indicated. Scale bars: white, 10 mm; black, 1 mm.


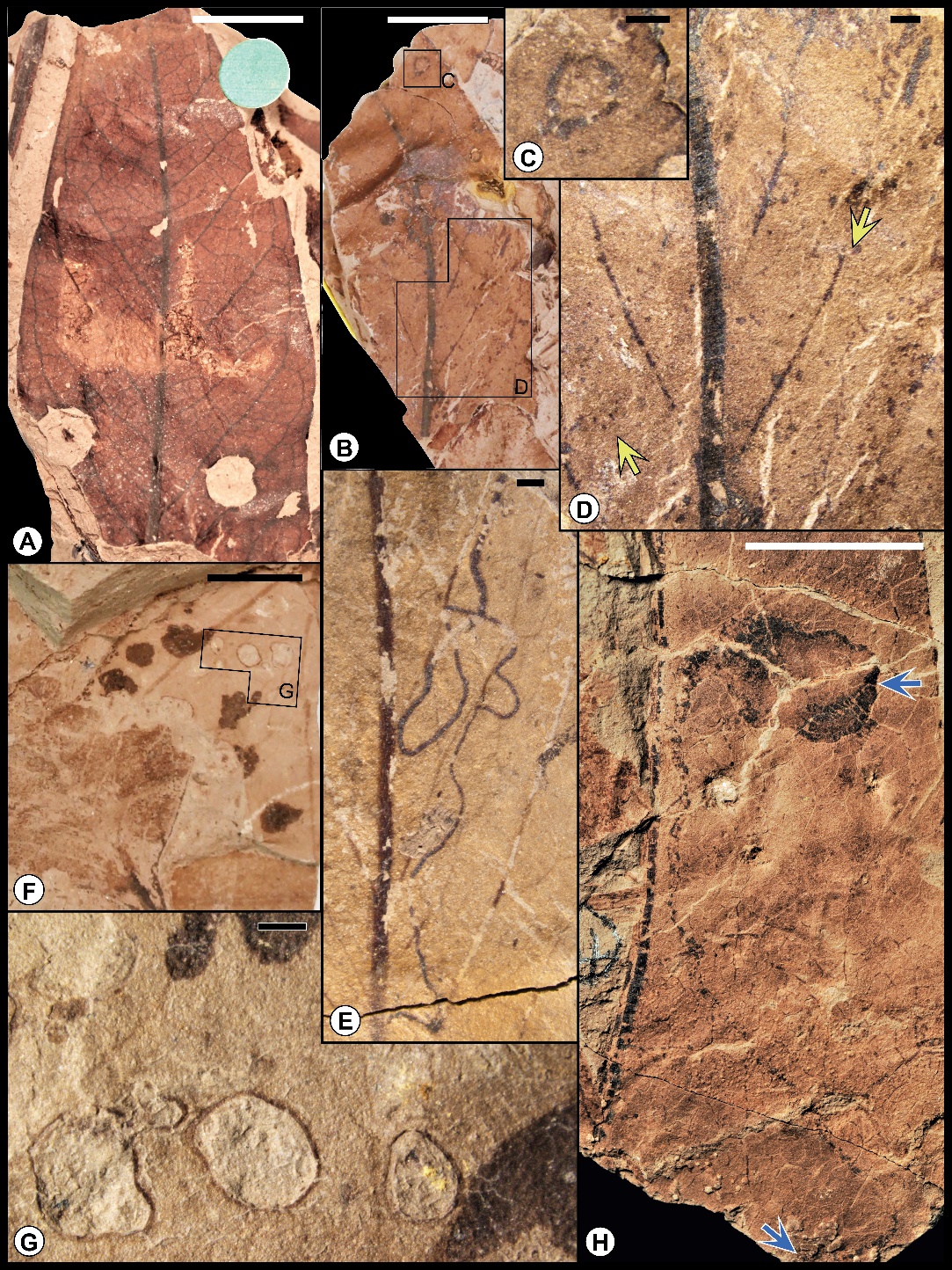


**(l) Fig. S12.** The component herbivore community at (A–E) of New Genus A, showing four FFGs of hole feeding, piercing and sucking, mining, and pathogen damage. Specimen UF-16175 at (**A**) shows two examples of DT4 hole feeding, with the hole at left probably breached from taphonomic processes. This leaf also is associated with DT3 hole feeding. At (**B**) is UF-16199-3, showing ambient DT46 piercing and sucking, DT382 pathogen enlarged at (**C**), and DT381 pathogen necroses probably responsible for apical leaf deformation enlargement at (**D**), displaying three types of piercing and sucking: random DT46, DT138 (right yellow arrow) demarcating punctures along a major vein, and DT338 (left yellow arrow) indicating punctures unassociated with veins. At (**E**) is UF-16199-1, illustrating an unusual version of mine DT41.

The component herbivore community at (F–H) is *Didromophyllum* *basingeri*, showing three FFGs of hole feeding, mining, and pathogen damage. Specimen UF-16099 at (**F**) consists of DT2 and DT3 hole feeding, enlarged in (**G**), and circular to rounded polylobate necroses of DT381. At (**H**) is UF-12947 that houses the distinctive, rare leaf mine DT59 characterized by a frass trail with a minimal width increase and an outsized terminal chamber. Scale bars: white, 10 mm; black, 1 mm.


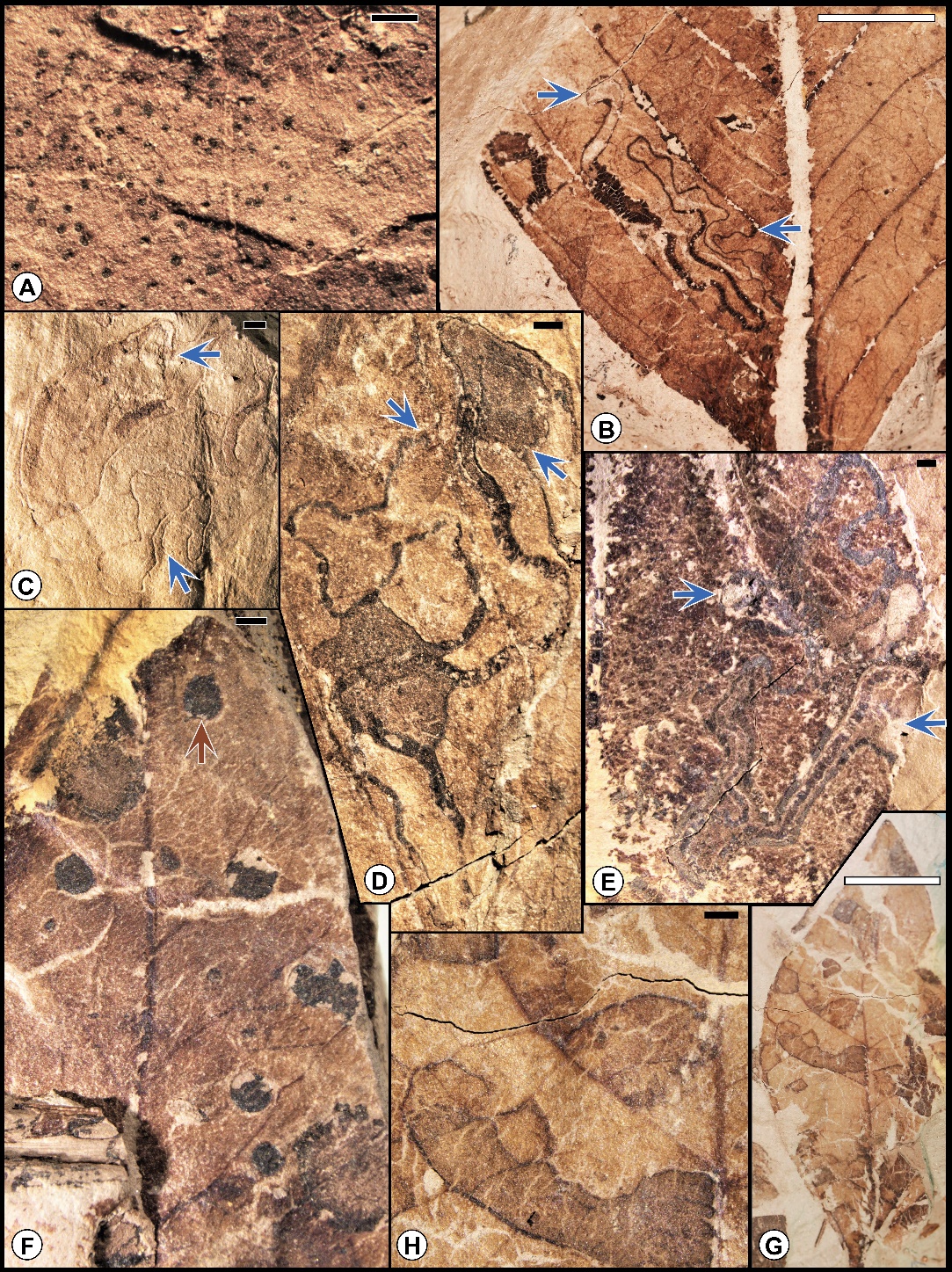


**(m) Fig. S13.** The

component herbivore communities of *Pandemophyllum* sp. 1 (A–E), records four FFGs of hole feeding, piercing and sucking, mining, and pathogen damage. Specimen UF-7855p at (**A**) shows piercing and sucking that occurs as random circular punctures (DT46), random elliptical punctures (DT47), fields of clustered punctures between adjacent veins (DT329), and highly arcuate to closed loop of punctures avoiding major veins (DT358). Specimen UF-16138 at (**B**) displays a complete DT234 mine with an oviposition site (upper blue arrow) and terminal chamber (lower blue arrow), and DT242 and DT261 fungal necroses in proximity to each other. UF-16134-1 at (**C**) shows a complete DT234 mine with oviposition site (bottom blue arrow), terminal chamber (top blue arrow), and adjacent DT3 holes (not shown). UF-12716 at (**D**) consists of two DT234 mines with an oviposition and a terminal chamber indicated (blue arrows) but that lack details of their frass trails, whereas UF-16141-1 at (**E**), harbors a well preserved DT234 mine with oviposition site and terminal chamber (blue arrows) and terminal chamber amid a darkened area among the two mines that represents a DT381 necrosis.

The component herbivore community of *Acritodromum* *ellipticum* on UF-16146-1 at (**F**) consists of about 11 necroses of the DT388 pathogen. This leaf also has DT381 pathogen necroses and a DT234 mine (not displayed).

The component herbivore community of *Reynoldsiophyllum* *nebrascense* in UF-16118-1 at (**G**) shows the ovoidal to polygonal pathogen necroses of DT381, enlarged in (**H**). Scale bars: white, 10 mm; black, 1 mm.


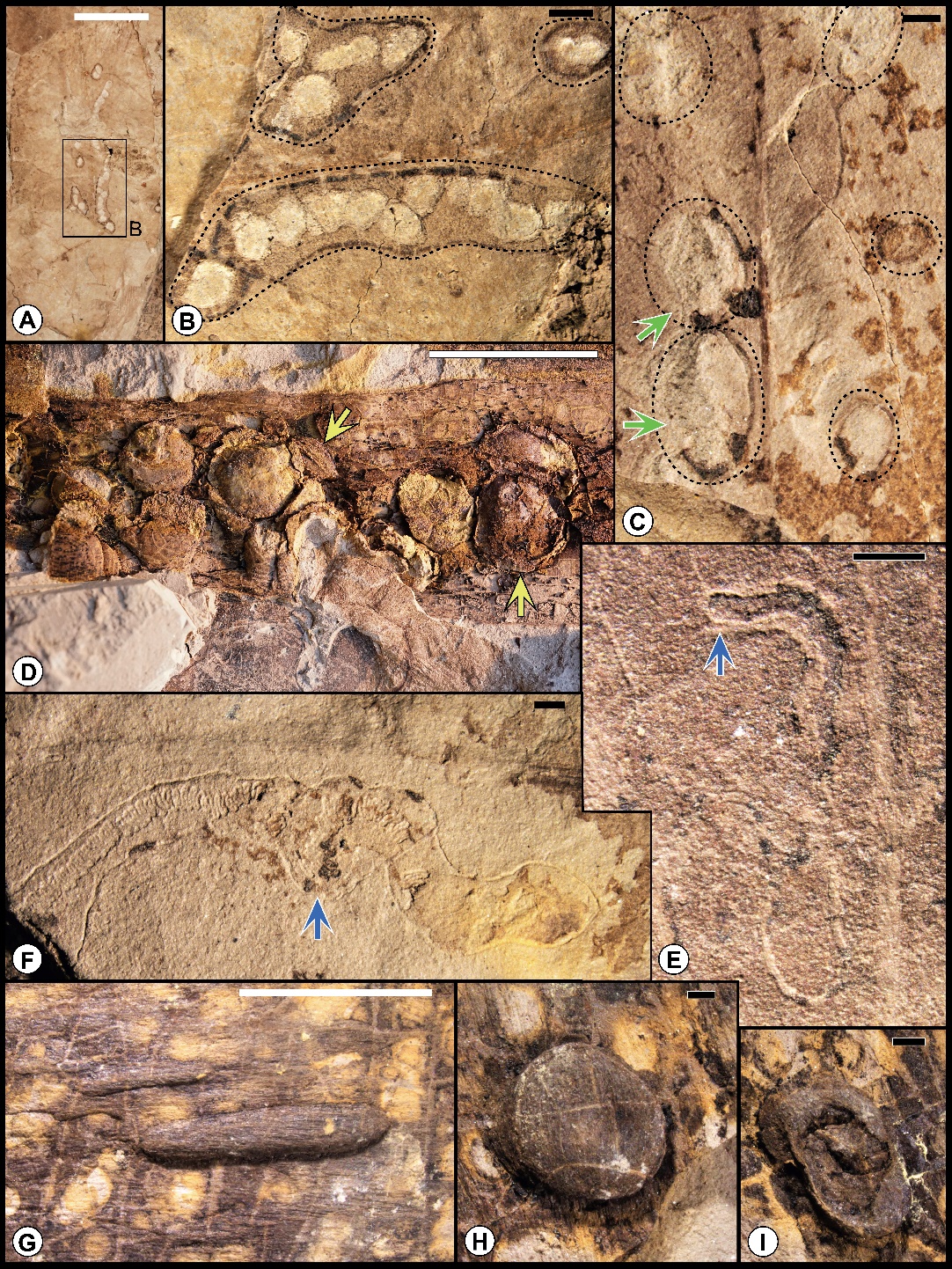


**(n) Fig. S14.** Herbivore associations on unidentified plant hosts I, displaying six FFGs of hole feeding, skeletonization, surface feeding, piercing and sucking, mining and borings. Specimen UF-12996 at (**A**) consists of several examples of DT276 surface feeding, enlarged in (**B**) that shows darkened tissue and darker reaction rims surrounding the 16 surface-fed cells (points with dash outline frame). UF-16221-2 in (**C**) displays DT29 surface feeding (green arrow with dash outline frame) and DT227 hole feeding that occur on both sides of a major vein. UF-12710 at (**D**) displays 13 mostly complete scale insect (DT394) coverings or their molds clustered on an unidentified twig; the scales have poorly developed instar growth rings and reveal immature scales (left yellow arrow) and adult scales (right yellow arrow). UF-16166-4 at (**E**) shows a DT41 mine that was aborted before completion of the terminal chamber (blue arrow). UF-16200-2 at (**F**) showing an excellently preserved end phases of a DT234 mine, revealing effects of the last instars prior to completion of the terminal chamber; the bulge of frass identified by the blue arrow indicates a larval instar molt. UF-16193-12 shows borings DT160 on a small, woody branch showing the boring in longitudinal (**G**), transverse (**H**) and oblique (**I**) views. Scale bars: white, 10 mm; black, 1 mm.


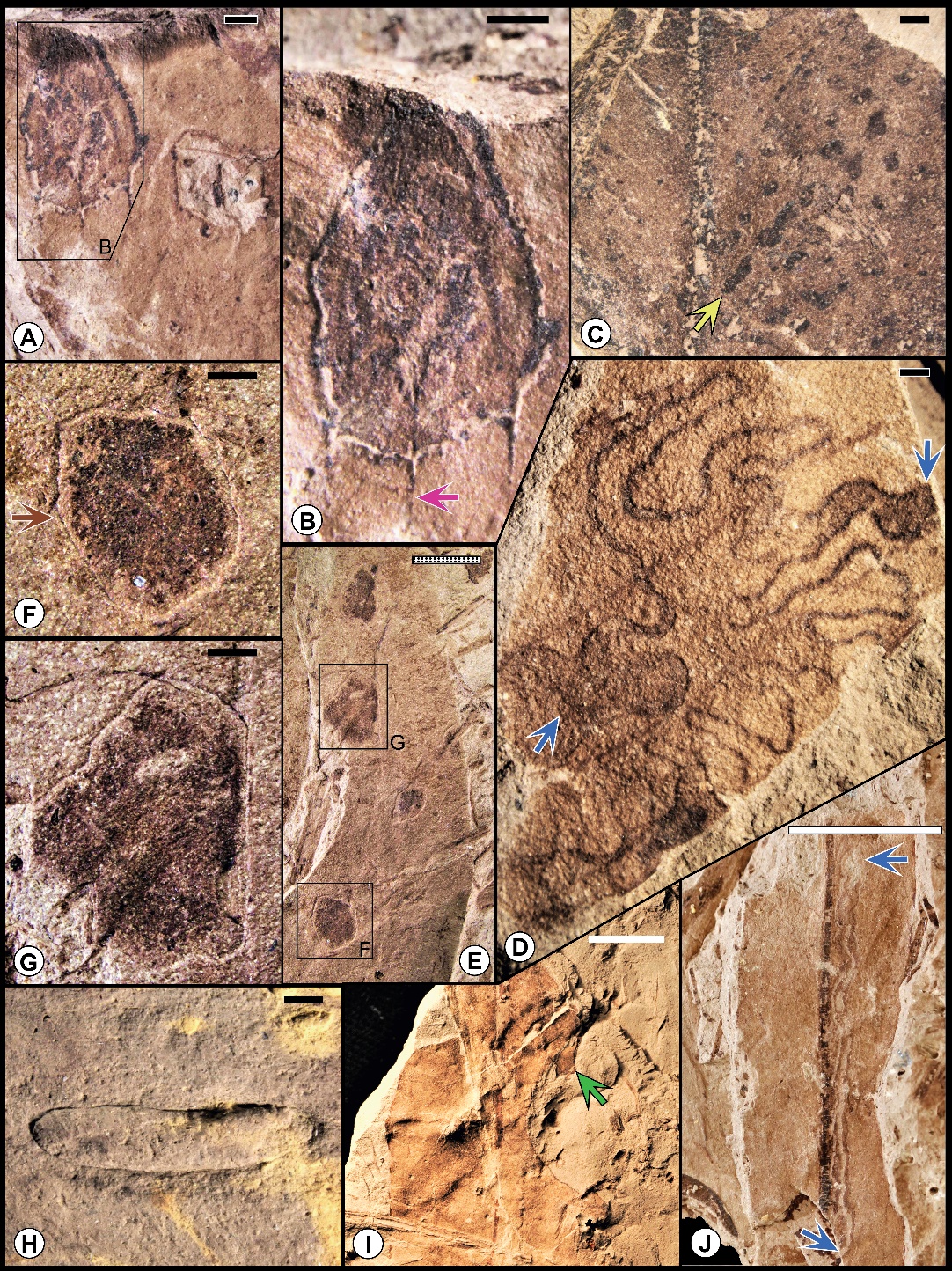


**(o) Fig. S15.** The herbivore associations on unidentified plant hosts II, displaying six FFGs of hole feeding, margin feeding, galling, mining, borings, and pathogen damage. Specimen UF-16197-17 at (**A**) shows DT3 and new gall DT398 distinguished by a thick outer wall with extensions into the adjacent minor and major veins, enlarged in (**B**) with one of the extensions indicated (magenta arrow). UF-4700-1 at (**C**) records linear rows of DT138 punctures along four major veins, one of which is demarcated (yellow arrow). UF-16122-4 at (**D**) consists of two, highly convoluted DT41 leaf mines with lobate terminal chambers (blue arrows). UF-16196-13 at (**E**) consists of several examples of pathogen DT388, with two necroses enlarged in (**F**) and (**G**) that straddle major veins and exhibit eccentrically placed inner areas that apparently bear fructifications. UF-16132-1 at (**H**) displays a longitudinal section of a boring DT160 on a small woody branch. UF-4882 at (**I**) consists of DT12 margin feeding, with continuous cusp formation indicated by a green arrow. UF-16126-2 at (**J**) shows a relatively short version of a DT234 mine, with the oviposition site and terminal chamber indicated by the bottom and top blue arrows, respectively. Scale bars: white, 10 mm; black, 1 mm.

**Appendix S14:** **Brief fossil records of the functional feeding groups**

The account below presents brief fossil histories of the 11 FFGs occurring on Rose Creek plants. The time intervals discussed below, in millions of years, are Devonian Period (419–359), Mississippian Period (359–323), Pennsylvanian Period (323–299), Permian Period (299–252), Triassic Period (252–201), Jurassic Period (201-145), Cretaceous Period (145–66), Paleogene Period (66–23), Neogene Period (23–2.8), and Quaternary Period (2.8–0) (Walker et al., 2018).

**(a) Hole feeding**

The earliest damage recorded by a hole feeder is on a foliose liverwort from the Middle Devonian (Labandeira et al., 2014). Very rare hole feeding damage reappears in latest Pennsylvanian floras (Xu et al., 2018), a pattern that extends to Permian floras (Beck and Labandeira, 1998; Labandeira et al., 2016). Hole feeding remains rarely encountered in Triassic floras (Labandeira et al., 2016, 2018) and in preangiospermous floras of the Early Cretaceous (Xiao et al., 2022). Hole feeding becomes more common in angiosperm dominated floras of the Late Cretaceous (Maccracken et al., 2021) and especially Cenozoic, where the spectrum of DTs increases substantially (Labandeira et al., 2002; Wappler et al., 2009).

**(b) Margin feeding**

The earliest occurrence of margin feeding occurs along the edges and tips of foliose liverworts from the Middle Devonian (Labandeira et al., 2014) and is followed by limited occurrences in the Mississippian (Iannuzzi and Labandeira, 2008). Unlike hole feeding, which is virtually absent during the Pennsylvanian, margin feeding is probably the most common FFG (Scott and Taylor, 1983) but consists only of three common modes of damage: DT12, DT13, DT14, and DT15 (Xu et al., 2018). This sparse pattern continues throughout the Permian (Schachat et al., 2014; Maccracken and Labandeira, 2020), doubling in the number of DTs by the Late Triassic (Labandeira et al., 2018), which remains the same in angiosperm dominated floras before and after the end-Cretaceous ecological crisis. An increase in a few margin feeding DTs is present in many Cenozoic floras (Labandeira, 2002).

**(c) Skeletonization**

Skeletonization has a poor fossil record and is absent in floras antedating the Permian. During the Permian, skeletonization is very rare and, when present, is associated with gigantopterid seedferns (Beck and Labandeira, 1998). The FFG has been anecdotally documented on a Late Triassic fern (Feng et al., 2014), where the FFG is also represented by one DT in a very highly sampled flora. Skeletonization remains uncommon in even highly sampled, angiosperm-dominated floras of the Paleogene (Currano et al., 2008), but becomes more conspicuous in Neogene floras (Dong et al., 2018).

**(d) Surface feeding**

Surface feeding has a pattern similar to skeletonization in the fossil record. The earliest evidence for surface feeding begins in the Middle Devonian with removal of surface cellular material from a foliose liverwort (Labandeira et al., 2014). During the Pennsylvanian, surface feeding is mainly found on ferns, seed ferns and Sphenophyllum (Xu et al., 2018; Correia et al., 2020). Surface feeding reappears in the Early Permian where it is more common than skeletonization and occurs mostly on gigantopterid seedferns (Beck and Labandeira, 1998; Schachat et al., 2015). The number of DTs doubles by the Late Triassic but remains uncommon in preangiospermous floras (Labandeira et al., 2016). At the Jurassic, surface feeding is relatively rare, only DT25, DT29, DT30 and DT103 have been recorded, mainly occurring on broad-leaved conifers, Bennettitinales, and cycads (Ding et al., 2015; Na et al., 2018; Santos et al., 2021). Surface feeding becomes more pervasive in angiosperm dominated floras of the Late Cretaceous (Maccracken et al., 2021) and Cenozoic (Wappler et al., 2012; Gunkel and Wappler, 2015; Donovan et al., 2016).

**(e) Oviposition**

Although oviposition lacks a record preceding the Pennsylvanian, there is a rich of a variety of oviposition damage mostly on plant stems beginning with the Pennsylvanian (Laaß and Hoff, 2015; Correia et al., 2020), contemporaneous with the earliest occurrences of winged insects (Béthoux et al., 2004). Ovipositional damage continues into the Permian, but mostly on foliage (Prevec et al., 2009; McLoughlin, 2011; Maccracken and Labandeira, 2020). Triassic floras display a similar spectrum of oviposition DTs as Paleozoic floras (Labandeira et al., 2018), but subsequent Jurassic and Early Cretaceous gymnosperm dominated floras have an increased variety of DTs on both stems and foliage (Gnaedinger et al., 2014; Na et al., 2014; Lin et al., 2019). Notably, ovipositional richness in one flora expanded to 15 DTs (Xiao et al., 2022). With the exception of DT349, oviposition at Rose Creek lacks distinctive, complex patterns. By contrast, the Cenozoic exhibits high ovipositional richness and the predominance of behaviorally complex DTs produced by damselflies (Hellmund and Hellmund, 2002; Sarzetti et al., 2009; Gnaedinger et al., 2014).

**(f) Piercing and sucking**

Evidence for piercing and sucking occurs in the Middle Devonian (Labandeira et al., 2014). Although piercing-and-sucking damage is minimal during almost all of the Pennsylvanian, in the latest part of the period there was a major expansion of damage by early lineages of mobile, hemipteroid insects and by presumably sessile scale insects (Xu et al., 2018). Evidence from both plant damage (Wang et al., 2009; Schachat et al., 2014, 2015) and body fossils (Shcherbakov et al., 2009) indicate an active piercing-and-sucking community during the Permian. By the end of the Triassic, the major groups of Hemiptera (cicadas, whiteflies, aphids, scale insects, and bugs), Thysanoptera (thrips), and probably mites were present (Krantz and Lindquist, 1979; Shcherbakov, 2008, 2011). The precursors to modern treehoppers, planthoppers and leafhoppers were present in the Upper Triassic (Shcherbakov, 2011), which may have produced various linear, curvilinear, meandering, and looping trajectories and clusters of stylet marks seen at Rose Creek (Figs. 4a, b, f; S6g, h, j; S8i; S13a). One Late Triassic flora harbors four DTs; little is known of Jurassic piercing and sucking; and one Early Cretaceous flora exhibits 13 DTs. While damage data is unavailable for Myanmar Amber, approximately four million years younger than the Rose Creek locality, apparently all major groups of Hemiptera were present (Szwedo, 2002, 2004). Piercing-and-sucking damage on Cenozoic floras appears to be less complicated, lacking most of the complex trajectories and clustered arrangements found at Rose Creek.

**(g) Mining**

The earliest occurrence of a leaf mine is from the Early Triassic (Krassilov and Karasev, 2008), likely made by a beetle. Definitive mines have yet to be verified in Paleozoic floras (Ding et al., 2014). Leaf mines are rare to common in Mesozoic floras (Rozefelds, 1988; Ding et al., 2014; Labandeira et al., 2018), and are structurally diverse, abundant, and occur on a wide variety of fern and seed-plant hosts from the Late Triassic of South Africa (Labandeira et al., 2018) and *Cladophlebis* ferns from the Okubata plant assemblage of Japan (Imada et al., 2022). Leaf mines have a sporadic occurrence in the Jurassic, consisting of single or a few occurrences in a few deposits (Xiao et al., 2022), and the ephemeral volcanic island (Eastern Spain) (Santos et al., 2021), though they occur with considerably greater diversity and or more plant-host species at Rose Creek. Leaf mines have been documented from three localities within about 10 million years before and after the Rose Creek plant assemblage. The mid Albian Patapsco Formation of Virginia, USA, at about 107 Ma (Jud and Sohn, 2016), yields a ranunculalean angiosperm host with possibly the earliest dipteran mine. Three localities of the Dakota Formation, including the Rose Creek locality (Labandeira et al., 1994; Doorenweerd et al., 2015), exhibit a diverse assemblage of leaf mines on early appearing angiosperms. The mid-Cenomanian beds at Nammoura, Lebanon, at 96 Ma, yielded a single mine on the ginkgophyte leaf *Pseudotorellia* sp. (Krassilov and Bacchia, 2000). The more plant diverse, mid-Turonian, Ora Formation at about 92 Ma, from the Negev of Israel, has produced diverse blotch and serpentine leaf mines on angiosperms (Krassilov, 2007, 2008; Krassilov and Rasnitsyn, 2008). Many modern leaf-mining lineages of modern Coleoptera, Lepidoptera, Diptera, and Hymenoptera are documented in numerous Cenozoic floras (Labandeira, 2002).

**(h) Galling**

The gall record of continental plants is an ancient one that extends to the Middle Devonian (Labandeira et al. 2014; Labandeira, 2021). The gall record of plants from the Pennsylvanian Period inhabiting coal swamps and adjacent environments consists of galls on axial tissues whereas the subsequent Permian Period records a shift to several basic forms of foliar galls (Schachat et al., 2015; Schachat and Labandeira, 2015). There are scattered occurrences of galls across Triassic and Jurassic floras (Labandeira et al., 2016, 2018; Ding et al., 2015; McLoughlin et al., 2015). The Rose Creek plant assemblage, however, represents a dramatic increase in gall morphotypes, documented by 25 DTs. The mid to late Cretaceous displays a spectrum of gall types that expanded considerably with angiosperm diversification (Scott et al., 1994; Vasilenko, 2005; Krassilov, 2007, 2008a, 2008b; Vasilenko et al., 2016). This expansion of gall morphotypes and DTs proliferated into the Cenozoic (Wappler, 2010; Knor et al., 2013; Gunkel and Wappler, 2015; Labandeira, 2021) and is supported by a variety of evolutionary studies (Nyman et al., 2000; Stireman III, 2010). Several, modern gall morphotypes produced by lineages of psyllids, pemphigids, and phylloxerids (Hemiptera); gall sawflies and gall wasps (Hymenoptera); gall midges (Diptera); and gall mites (Acari) are identical in structure to gall fossils found in the mid Cenozoic (Eocene) deposits of Northern Europe (Labandeira, 2021).

**(i) Seed predation**

Seed predation extends to the Early Pennsylvanian (Scott and Taylor, 1983; Labandeira, 2002). During the early Permian, seed predation expanded to several seed-plant hosts, such as cordaites from a glossopterid flora from Southeastern Brazil that included four DTs (Dos Santos et al., 2020), and late Paleozoic, Angaran flora from central Russia (Sharov, 1973; Shcherbakov et al., 2009). Although three DTs on seeds are documented from the Late Triassic of South Africa (Labandeira et al., 2018), seed predation has a spotty fossil record to the recent (Collinson and Hooker, 1991; Mikulás et al., 1998; Labandeira et al., 2018). Given the abundance of megagametophytic tissues in gymnosperms and endosperm in angiosperms, it appears that seed predation is an underutilized feeding strategy throughout the fossil record.

**(j) Borings**

Because of the habitats that insects occupy to produce borings, their occurrence in the fossil record is associated with three-dimensionally preserved carbonate or silica permineralized fossil wood. Pennsylvanian occurrences are limited, overwhelmingly present as carbonate permineralized axes of coal-swamp-associated trees (Labandeira and Phillips, 2002). During the Permian there were rare occurrences of borings in silicified punky wood (Naugolynkh and Ponomarenko, 2010) and in conifer trunks (Feng et al., 2017). Mesozoic occurrences are limited to conifer wood from the Late Triassic of Arizona, USA (Tapanila and Roberts, 2012), Middle Jurassic of Argentina (García-Massini, 2012), and earliest Cretaceous of Brazil (Pires and Sommer, 2009). The incidence of insect borings in Cenozoic permineralized woods increases considerably (Radwanski, 1977; Rajchel and Uchman, 1998; Labandeira et al., 2001). Apparently, insect borings in Cretaceous angiosperm woods are very rare but become more abundant in the Cenozoic.

**(k) Pathogens**

The fossil history of pathogens on adpression floras has been poorly documented even though the microfossil record of fungi is occasionally prolific (Krings et al., 2013; Labandeira and Prevec, 2014). Documentation of pathogens in Paleozoic deposits often involved descriptions of fungi in permineralized floras. These include fungal morphotypes that are parasitic, saprotrophic and commensalistic in Paleozoic land plants of Lower Devonian (Taylor and Osborn, 1996; Krings et al., 2013), Pennsylvanian (Dotzler et al., 2011; Krings et al., 2011, 2013), and Permian (Srivastava, 1993; Visscher et al., 2011; Slater et al., 2013; Harper et al., 2016) age. By contrast, mention of fungal damage in Paleozoic adpression floras virtually is absent, as is mention of Late Triassic (Labandeira et al., 2018) and Middle Jurassic (García-Massini et al., 2012) fungal damage. For the Cretaceous, several studies have documented pathogen interactions. These include a white rot fungus described on a conifer from northeastern China (Tian et al., 2020); occurrences from Russia documenting fungal micromorphological detail (Maslova et al., 2016); and evidence supporting insect fungivory from fecal pellets in amber (Schmidt et al., 2010). Cenozoic pathogens typically include well-preserved fungal mycelia, fruiting bodies, and dispersed spores. From Eocene deposits, well-preserved epiphyllous fungi and occasional bacteria have been described from descriptions mostly of dispersed spores from Texas (Daghlian, 1978), Tennessee (Dilcher, 1963; Sheffy and Dilcher, 1971), Baltic amber (Kohring, 1995), and leaf surfaces from New Zealand (Bannister et al., 2016). Pathogens from Neogene deposits include epiphyllous fungi on conifers (Shi et al., 2010) and ascospore-laden resinicolous fungi (Rikkinen and Poinar, 2000).

**Appendix S15: Host-plant specialization data.** Generalized, intermediate and specialized damage types (DTs) on host plants

| Functional Feeding Group and Damage Type | Generalized | Intermediate | Specialized | FFG SI^1^ |
| --- | --- | --- | --- | --- |
| **Hole Feeding** |  |  |  | **1.5** |
| DT 001 | **X** |  |  |  |
| DT 002 | **X** |  |  |  |
| DT 003 | **X** |  |  |  |
| DT 004 |  | **X** |  |  |
| DT 005 | **X** |  |  |  |
| DT 007 |  |  | **X** |  |
| DT 009 | **X** |  |  |  |
| DT 050 | — | — | — |  |
| DT 063 | — | — | — |  |
| DT 126 | — | — | — |  |
| DT 227 |  | **X** |  |  |
| **Margin Feeding** |  |  |  | **1.3** |
| DT 012 | **X** |  |  |  |
| DT 013 | **X** |  |  |  |
| DT 014 | **X** |  |  |  |
| DT 015 | **X** |  |  |  |
| DT 081 |  |  | **X** |  |
| DT 198 | **X** |  |  |  |
| DT 291 | — | — | — |  |
| DT 405 |  |  | **X** |  |
| **Skeletonization** |  |  |  | **1** |
| DT 017 | **X** |  |  |  |
| DT 024 | — | — | — |  |
| DT 056 | — | — | — |  |
| DT 061 | **X** |  |  |  |
| DT 079 | — | — | — |  |
| **Surface Feeding** |  |  |  | **1.6** |
| DT 029 | **X** |  |  |  |
| DT 030 | **X** |  |  |  |
| DT 031 | — | — | — |  |
| DT 276 |  |  | **X** |  |
| DT 333 | — | — | — |  |
| **Oviposition** |  |  |  | **1.3** |
| DT 072 | **X** |  |  |  |
| DT 076 | **X** |  |  |  |
| DT 101 | **X** |  |  |  |
| DT 175 |  | **X** |  |  |
| DT 226 | **X** |  |  |  |
| DT 228 |  | **X** |  |  |
| DT 285 | — | — | — |  |
| DT 331 | — | — | — |  |
| DT 349 | — | — | — |  |
| **Piercing and Sucking** |  |  |  | **1.2** |
| DT 046 | **X** |  |  |  |
| DT 047 | **X** |  |  |  |
| DT 048 | **X** |  |  |  |
| DT 077 | **X** |  |  |  |
| DT 138 | **X** |  |  |  |
| DT 183 | — | — | — |  |
| DT 184 | — | — | — |  |
| DT 281 | **X** |  |  |  |
| DT 330 | **X** |  |  |  |
| DT 338 | **X** |  |  |  |
| DT 344 | — | — | — |  |
| DT 358 |  |  | **X** |  |
| DT 383 | **X** |  |  |  |
| DT 392 |  |  | **X** |  |
| DT 394 | **X** |  |  |  |
| DT 402 |  |  | **X** |  |
| DT 406 | **X** |  |  |  |
| **Mining** |  |  |  | **1.3** |
| DT 036 | — | — | — |  |
| DT 040 | — | — | — |  |
| DT 041 | **X** |  |  |  |
| DT 043 |  | **X** |  |  |
| DT 044 | **X** |  |  |  |
| DT 045 | — | — | — |  |
| DT 059 | — | — | — |  |
| DT 060 |  | **X** |  |  |
| DT 091 | — | — | — |  |
| DT 092 | — | — | — |  |
| DT 093 | **X** |  |  |  |
| DT 105 | — | — | — |  |
| DT 109 | **X** |  |  |  |
| DT 141 | — | — | — |  |
| DT 176 | **X** |  |  |  |
| DT 202 | — | — | — |  |
| DT 208 |  | **X** |  |  |
| DT 210 | — | — | — |  |
| DT 234 | **X** |  |  |  |
| DT 288 | — | — | — |  |
| **Galling** |  |  |  | **2** |
| DT 034 | **X** |  |  |  |
| DT 052 | — | — | — |  |
| DT 080 | **X** |  |  |  |
| DT 085 | — | — | — |  |
| DT 087 | — | — | — |  |
| DT 106 | — | — | — |  |
| DT 117 | — | — | — |  |
| DT 119 |  | **X** |  |  |
| DT 120 |  |  | **X** |  |
| DT 122 | **X** |  |  |  |
| DT 145 | — | — | — |  |
| DT 153 |  |  | **X** |  |
| DT 188 | **X** |  |  |  |
| DT 189 |  |  | **X** |  |
| DT 194 |  |  | **X** |  |
| DT 205 |  |  | **X** |  |
| DT 247 | — | — | — |  |
| DT 254 | **X** |  |  |  |
| DT 265 |  |  | **X** |  |
| DT 266 | **X** |  |  |  |
| DT 289 | **X** |  |  |  |
| DT 303 | — | — | — |  |
| DT 359 | — | — | — |  |
| DT 386 |  | **X** |  |  |
| DT 398 |  | **X** |  |  |
| **Seed Predation** |  |  |  | — |
| DT 074 | — | — | — |  |
| **Wood Boring** |  |  |  | **1** |
| DT 160 | **X** |  |  |  |
| DT 284 | **X** |  |  |  |
| **Pathogen** |  |  |  | **1.5** |
| DT 058 | **X** |  |  |  |
| DT 066 |  | **X** |  |  |
| DT 069 | **X** |  |  |  |
| DT 174 |  |  | **X** |  |
| DT 242 |  | **X** |  |  |
| DT 261 | **X** |  |  |  |
| DT 381 | **X** |  |  |  |
| DT 382 | **X** |  |  |  |
| DT 385 |  | **X** |  |  |
| DT 387 |  | **X** |  |  |
| DT 388 | **X** |  |  |  |
| **Totals (Proportion):** | **50** | **14** | **14** |  |
|  | **64.10%** | **17.95%** | **17.95%** |  |

Note:

1. SI is the Specialization Index, in this table for the functional feeding groups (FFGs).

**____________________________________________________________________________________**

**Appendix S16:** **Percentage removal of host plant herbivorized surface area**

| **Plant group** | **Plant taxa** | **Examined specimens** | | **Herbivorized specimens** | | **Total leaf area** | | **Herbivorized**  **leaf area (cm^2^)** | **HI^1^** | **DT richness** | **DT occur.** | **Feeding event occur.** | **SI^2^** |
| --- | --- | --- | --- | --- | --- | --- | --- | --- | --- | --- | --- | --- | --- |
|  |  | **No.** | **ratio (%)** | **No.** | **ratio (%)** | **area (cm^2^)** | **ratio (%)** |  |  |  |  |  |  |
| Lycopods | **Isoetales** | **1** | **0.05** | **0** | **0** | **1.59** | **0.01** | **0** | **0** | **0** | **0** | **0** | **—** |
|  | *Isoetites* sp. | 1 | 0.05 | 0 | 0 | 1.59 | 0.01 | 0 | 0 | 0 | 0 | 0 |  |
| Fern | **Filicales** | **20** | **0.96** | **2** | **0.21** | **91.14** | **0.76** | **0.89** | **0.98** | **2** | **2** | **3** | **1** |
|  | *Cladophlebis inclinata* | 17 | 0.82 | 1 | 0.11 | 68.81 | 0.58 | 0.80 | 1.16 | 1 | 1 | 2 |  |
|  | *Cladophlebis* *parva* | 1 | 0.05 | 1 | 0.11 | 11.38 | 0.10 | 0.10 | 0.84 | 1 | 1 | 1 |  |
|  | *Cladophlebis* sp. | 2 | 0.10 | 0 | 0 | 10.94 | 0.09 | 0 | 0 | 0 | 0 | 0 |  |
|  | **Gleicheniales** | **5** | **0.24** | **1** | **0.11** | **8.70** | **0.07** | **0.05** | **0.58** | **1** | **1** | **2** | **1** |
|  | *Gleichenia* *delicatula* | 1 | 0.05 | 0 | 0 | 0.54 | 0.00 | 0 | 0 | 0 | 0 | 0 |  |
|  | *Gleichenia* sp. | 1 | 0.05 | 0 | 0 | 3.80 | 0.03 | 0 | 0 | 0 | 0 | 0 |  |
|  | *Matonidium americanum* | 2 | 0.10 | 1 | 0.11 | 3.92 | 0.03 | 0.05 | 1.28 | 1 | 1 | 2 |  |
|  | *Matonidium brownii* | 1 | 0.05 | 0 | 0 | 0.44 | 0.00 | 0 | 0 | 0 | 0 | 0 |  |
|  | **Schizaeales** | **3** | **0.14** | **0** | **0** | **16.76** | **0.14** | **0** | **0** | **0** | **0** | **0** | **—** |
|  | *Anemia dicksoniana* | 1 | 0.05 | 0 | 0 | 4.29 | 0.04 | 0 | 0 | 0 | 0 | 0 |  |
|  | Fern Type 2 | 2 | 0.10 | 0 | 0 | 12.48 | 0.10 | 0 | 0 | 0 | 0 | 0 |  |
| Gymno-sperm | **Pinales** | **14** | **0.67** | **2** | **0.21** | **22.81** | **0.19** | **0.03** | **0.13** | **3** | **3** | **9** | **1.3** |
|  | Conifer Foliage | 13 | 0.62 | 2 | 0.21 | 22.57 | 0.19 | 0.03 | 0.13 | 3 | 3 | 9 |  |
|  | Conifer Cone | 1 | 0.05 | 0 | 0 | 0.23 | 0.00 | 0 | 0 | 0 | 0 | 0 |  |
| Angiosperms (vegetative) | **Austrobaileyales** | **58** | **2.78** | **11** | **1.16** | **244.08** | **2.05** | **1.61** | **0.66** | **7** | **11** | **65** | **1** |
|  | *Longstrethia varidentata* | 58 | 2.78 | 11 | 1.16 | 244.08 | 2.05 | 1.61 | 0.66 | 7 | 11 | 65 |  |
|  | **Chloranthales** | **307** | **14.73** | **238** | **25.03** | **1939.12** | **16.27** | **116.62** | **6.01** | **86** | **458** | **2485** | **1.22** |
|  | *Crassidenticulum decurrens* | 248 | 11.90 | 201 | 21.14 | 1640.60 | 13.77 | 98.45 | 6.00 | 53 | 395 | 2150 |  |
|  | *Crassidenticulum* sp. | 2 | 0.10 | 0 | 0 | 4.67 | 0.04 | 0 | 0 | 0 | 0 | 0 |  |
|  | *Densinervum kaulii* | 56 | 2.69 | 36 | 3.79 | 278.44 | 2.34 | 18.07 | 6.49 | 32 | 62 | 332 |  |
|  | *Lanbonia calophylla* | 1 | 0.05 | 1 | 0.11 | 15.41 | 0.13 | 0.10 | 0.63 | 1 | 1 | 3 |  |
|  | **Laurales** | **618** | **29.65** | **347** | **36.49** | **4597.66** | **38.58** | **168.37** | **3.66** | **144** | **585** | **3365** | **1.01** |
|  | *Pabiania variloba* | 239 | 11.47 | 134 | 14.09 | 1627.76 | 13.66 | 80.16 | 4.92 | 43 | 208 | 1380 |  |
|  | *Pandemophyllum attenuatum* | 115 | 5.52 | 40 | 4.21 | 585.66 | 4.91 | 7.17 | 3.19 | 36 | 67 | 217 |  |
|  | *Pandemophyllum kvacekii* | 244 | 11.71 | 158 | 16.61 | 2178.73 | 18.28 | 74.34 | 3.41 | 53 | 289 | 1685 |  |
|  | *Pandemophyllum* sp*.* | 20 | 0.96 | 15 | 1.58 | 205.51 | 1.72 | 6.70 | 3.26 | 12 | 21 | 83 |  |
|  | **Magnoliales** | **9** | **0.43** | **6** | **0.63** | **99.13** | **0.83** | **2.32** | **2.34** | **7** | **11** | **52** | **1** |
|  | New Genus A | 9 | 0.43 | 6 | 0.63 | 99.13 | 0.83 | 2.32 | 2.34 | 7 | 11 | 52 |  |
|  | **Magnoliidae Unplaced** | **34** | **1.63** | **18** | **1.89** | **366.53** | **3.08** | **8.65** | **2.36** | **25** | **30** | **105** | **1.03** |
|  | *Acritodromum ellipticum* | 8 | 0.38 | 6 | 0.63 | 63.06 | 0.53 | 1.88 | 2.99 | 7 | 9 | 20 |  |
|  | *Dicotylophyllum angularis* | 4 | 0.19 | 1 | 0.11 | 36.90 | 0.31 | 1.96 | 5.31 | 3 | 3 | 10 |  |
|  | *Didromophyllum basingerii* | 16 | 0.77 | 8 | 0.84 | 217.35 | 1.82 | 3.55 | 1.64 | 10 | 12 | 49 |  |
|  | *Reynoldsiophyllum nebrascense* | 5 | 0.24 | 3 | 0.32 | 47.46 | 0.40 | 1.25 | 2.64 | 5 | 6 | 26 |  |
|  | New Genus B | 1 | 0.05 | 0 | 0 | 1.77 | 0.01 | 0 | 0 | 0 | 0 | 0 |  |
|  | **Eurosidae II** | **34** | **1.63** | **26** | **2.73** | **271.33** | **2.28** | **24.48** | **9.02** | **30** | **59** | **269** | **1.09** |
|  | *Anisodromum wolfei* | 32 | 1.54 | 24 | 2.52 | 245.24 | 2.06 | 22.76 | 9.28 | 26 | 54 | 255 |  |
|  | *Citrophyllum doylei* | 2 | 0.10 | 2 | 0.21 | 26.10 | 0.22 | 1.72 | 6.59 | 4 | 5 | 14 |  |
|  | **Unplaced Vegetative** | **15** | **0.72** | **9** | **0.95** | **187.68** | **1.57** | **5.15** | **2.74** | **11** | **13** | **33** | **1** |
|  | *Wingia expansolobum* | 1 | 0.05 | 0 | 0 | 22.84 | 0.19 | 0 | 0 | 0 | 0 | 0 |  |
|  | *Dicotylophyllum microserratom* | 6 | 0.29 | 3 | 0.32 | 41.05 | 0.34 | 0.15 | 0.37 | 4 | 4 | 4 |  |
|  | *Dicotylophyllum myrtophylloides* | 2 | 0.10 | 1 | 0.11 | 7.17 | 0.06 | 0.07 | 0.98 | 1 | 1 | 1 |  |
|  | *Dicotylophyllum rosafluviatilis* | 6 | 0.29 | 5 | 0.53 | 116.62 | 0.98 | 4.93 | 4.23 | 6 | 8 | 28 |  |
| Angiosperms (reproductive) | **Unplaced Reproductive** | **87** | **4.17** | **9** | **0.95** | **225.60** | **1.89** | **0.17** | **0.08** | **4** | **10** | **21** | **1.86** |
|  | *Dakotanthus cordiformis* | 38 | 1.82 | 8 | 0.84 | 58.15 | 0.49 | 0.17 | 0.30 | 3 | 9 | 20 |  |
|  | Flower Type 1 | 3 | 0.14 | 0 | 0 | 0.85 | 0.01 | 0 | 0 | 0 | 0 | 0 |  |
|  | Flower Type 2 | 2 | 0.10 | 0 | 0 | 0.06 | 0.00 | 0 | 0 | 0 | 0 | 0 |  |
|  | Reproductive Morphotype 1 | 9 | 0.43 | 0 | 0 | 12.29 | 0.10 | 0 | 0 | 0 | 0 | 0 |  |
|  | Reproductive Morphotype 2 | 5 | 0.24 | 0 | 0 | 10.01 | 0.08 | 0 | 0 | 0 | 0 | 0 |  |
|  | Reproductive Morphotype 3 | 2 | 0.10 | 0 | 0 | 1.58 | 0.01 | 0 | 0 | 0 | 0 | 0 |  |
|  | Reproductive Morphotype 4 | 1 | 0.05 | 0 | 0 | 0.57 | 0.00 | 0 | 0 | 0 | 0 | 0 |  |
|  | Reproductive Morphotype 5 | 1 | 0.05 | 0 | 0 | 0.41 | 0.00 | 0 | 0 | 0 | 0 | 0 |  |
|  | Other Reproductive Morphotype | 12 | 0.58 | 0 | 0 | 8.00 | 0.07 | 0 | 0 | 0 | 0 | 0 |  |
|  | Seed Type 1 | 1 | 0.05 | 0 | 0 | 0.04 | 0.00 | 0 | 0 | 0 | 0 | 0 |  |
|  | Seed Type 2 | 2 | 0.10 | 0 | 0 | 0.69 | 0.01 | 0 | 0 | 0 | 0 | 0 |  |
|  | Seed Type 3 | 1 | 0.05 | 0 | 0 | 0.12 | 0.00 | 0 | 0 | 0 | 0 | 0 |  |
|  | Seed Type 4 | 1 | 0.05 | 0 | 0 | 0.17 | 0.00 | 0 | 0 | 0 | 0 | 0 |  |
|  | Seed Type 5 | 9 | 0.43 | 1 | 0.11 | 132.63 | 1.11 | 0.00 | 0 | 1 | 1 | 1 |  |
| Unidentifiable | **Unidentifiable Material** | **879** | **42.18** | **282** | **29.65** | **3844.69** | **32.26** | **46.10** | **1.20** | **62** | **371** | **1948** | **1.06** |
|  | Dicot Leaf Fragments | 745 | 35.75 | 269 | 28.29 | 3116.78 | 26.15 | 42.76 | 1.37 | 53 | 357 | 1846 |  |
|  | Unidentifiable Axes | 134 | 6.43 | 13 | 1.37 | 727.91 | 6.11 | 3.34 | 0.46 | 9 | 14 | 102 |  |
| **Total / percent sums** | | **2084** | **100** | **951** | **100** | **11916.83** | **100** | **374.45** | **3.14** | **114** | **1554** | **8357** | **1.13** |

1. HI is the herbivory index, or the percentage of herbivorized surface area.

2. The specialization index (SI) is the plant host specialization assignment based on the damage type (DT): 1, generalized; 2, intermediate; and 3, specialized (Wilf and Labandeira, 1999; Labandeira et al., 2007). The data for calculation of the SI are same as Appendices S15.

3. The total examined specimens are preserved on 353 slabs. Because several slabs preserved more than one leaf, the number of examined specimens (2084) is larger than 353.

**Appendix S17: Composition of plant taxa analyzed by NMDS**

Because many plant hosts are represented by just a few specimens, species other than *Longstrethia varidentata* Upchurch and Dilcher (Austrobaileyales) were lumped into higher-order taxonomic categories for a nonmetric multidimensional scaling analyses (NMDS), as follows: ferns (*Cladophlebis inclinata* Skog and Dilcher, *Cladophlebis parva* Skog and Dilcher, *Cladophlebis* sp. 1, *Gleichenia delicatula* Skog and Dilcher, *Gleichenia* sp. 1, *Matonidium americanum* Skog and Dilcher, and *Matonidium brownii* Skog and Dilcher); Chloranthales (*Crassidenticulum decurrens* (Lesquereux), *Crassidenticulum* sp. 1, *Densinervum kaulii* Upchurch and Dilcher, and *Landonia calophylla* Upchurch and Dilcher); Laurales (*Pabiania variloba* Upchurch and Dilcher, *Pandemophyllum kvacekii* Upchurch and Dilcher, and *Pandemophyllum attenuatum* Upchurch and Dilcher); Eurosidae (*Anisodromum wolfei* Upchurch and Dilcher, and *Citrophyllum doylei* Upchurch and Dilcher); and “*Dicotylophyllum*” (*Dicotylophyllum angularis* Upchurch and Dilcher, *Wingia expansolobum* (Upchurch and Dilcher) Wang and Dilcher, *Dicotylophyllum microserratum* Upchurch and Dilcher, *Dicotylophyllum myrtophylloides* Upchurch and Dilcher, *Dicotylophyllum rosafluviatilis* Upchurch and Dilcher, and *Didromophyllum basingerii* Upchurch and Dilcher). As the ferns are represented by only 100 cm^2^ of surface area, the subsampling procedure was carried out twice. First, each plant host was subsampled to only 50 cm^2^ of surface area to accommodate the ferns. Later, the ferns were removed from the dataset and the remaining plant hosts, represented by at least 244 cm^2^ of surface area each, were subsampled to 200 cm^2^ of surface area.

**Appendix S18: New damage type (DT) descriptions**

Fourteen damage types (DTs) are described below that are new to the Rose Creek plant assemblage that were collected from the Janssen Clay Member of the Dakota Formation. The Rose Creek locality, near Fairbury, Jefferson County, Nebraska, U.S.A. is from the Early Cretaceous period and is assigned to the late Albian stage, pegged to a date of approximately 103 Ma. The new DTs consist of six functional feeding groups (FFGs) and consist of one DT of surface feeding FFG (DT276), two DTs of piercing and sucking FFG (DT383 and DT392), one DT of mining FFG (DT234), two DTs of galling FFG (DT386 and DT398), one DT of borings FFG (DT160), and seven DTs of pathogens FFG (DT174, DT242, DT381, DT382, DT385, DT387 and DT388). For host specificities of these DTs, see Appendix S15 above. These specimens are deposited at the paleobotanical collections of Florida Museum of Natural History, in Gainesville, Florida, U.S.A. See Upchurch and Dilcher (1990) for additional details for paleobotanical data of these plant specimens. Two additional DTs on flowers, DT402 (piercing and sucking), and DT405 (margin feeding) are described for Rose Creek flowers (Xiao et al., 2021a); three additional piercing-and-sucking DTs on stems and leaves, DT384, DT394 and DT406, are discussed in a separate contribution on Rose Creek scale insects (Xiao et al., 2021b).

**(a) DT276 (Surface feeding)**

*Description*: Foliar surface feeding characterized by a linear succession of small, more or less equant patches of a removed upper foliar tissue layer, each 0.5 to 1.5 mm in longest dimension, the entire structure approximately 2 to 10 mm in longest dimension, bounded principally by a primary vein such as a midrib but frequently extending somewhat along adjacent, branching secondary veins; the damage often with contractions and expansions in width, and displaying confluent, bordering reaction rims; present along primary and secondary veins anywhere on a leaf blade.

*Figured specimens*: UF-15713-12996 (Fig. S14A, S14B).

*Feeding event occurrence data*: Pattern occurrence.

*Specialization level*: 3, at Rose Creek.

*Host plant*: Unknown eudicot angiosperm.

*Inferred herbivore*: Unknown; probably Orthoptera or Coleoptera.

*Modern ecological analog*. The damage made by the unassigned weevil, Curculionidae (Coleoptera: Curculionidae) on guasimo colorado, *Luehea* *seemannii* Triana and Planch. (Malvaceae), from Panama (Carvalho et al., 2014).

*Remarks*: This type of surface feeding is frequently confused with skeletonization because the tertiary veins are typically exposed. However, it is the upper surfaces of the veins that are uncovered, and the veins lay on top of a layer of epidermis, with most but not all of the layered leaf tissues removed.

**(b) DT383 (Piercing and sucking)**

*Description*: Moderately dense to sparser, variably shaped clusters of punctures occurring on leaf-blade regions irrespective of veins; each cluster from 2 to 4 mm in maximum dimension on the leaf surface, consisting of 40 to 100 punctures mostly organized in linear to meandering, occasionally intersecting files; the punctures have spacing ranging from touching to several puncture diameters apart, and consist of dark spots, some containing a central hollow depression, and diameters of 0.1 to 0.2 mm; this damage occurs on all regions of the leaf blade irrespective of venation.

*Figured specimens*: UF-15713-7516 (Fig. 2F), UF-15713-12686 (Fig. 3L, 3M), UF-15713-16160 (Fig. 4A, 4B), UF-15713-16112 (Fig. 4C), UF-15713-7247 (Fig. 4F).

*Other material*: UF-15713-4450, UF-15713-4824, UF-15713-4906, UF-15713-4938, UF-15713-5267, UF-15713-5897, UF-15713-62654, UF-15713-7815, UF-15713-78163, UF-15713-78195, UF-15713-16117.

*Feeding event occurrence data*: Pattern occurrence.

*Specialization level*: 1, at Rose Creek.

*Host* *plant*: *Pandemophyllum attenuatum* Upchurch and Dilcher, 1990 (Lauraceae).

*Inferred herbivore*: Several major groups (Hemiptera: Auchenorrhyncha) of treehoppers, leafhoppers and planthoppers produce such clusters of punctures, particularly on mesophyll between secondary veins.

*Modern ecological analog*: The Potato Leafhopper, *Eurypteryx* *aurata* L. (Hemiptera: Cicadellidae), on potato, *Solanum* *tuberosum* L. (Solanaceae), from Devon, England.

*Remarks*: Each cluster of closely spaced to touching to rarely overlapping punctures is a feeding event, and thus constitutes a patterned feeding event. Some leaves have one to several such clusters, indicating that the leaf was extensively used as a resource by multiple individuals. This pattern of damage is particularly notable for *Crassidenticulum* *decurrens* and *Pandemophyllum* *kvacekii*.

**(c) DT392 (Piercing and sucking)**

*Description*: One to four linear or occasionally more irregular rows of numerous punctures occurring on a midrib or other major vein but not on adjacent blade tissue; each row contains irregularly spaced, circular to elliptical punctures whose cross-sections are from 0.1 to 0.50 mm in longest dimension; the presence of this distinctive pattern of punctures indicates the direct access of phloem and possibly xylem from the underlying vascular tissue and can occur on any part of the leaf with major veins.

*Specimens*: UF-15713-7250, UF-15713-28926, UF-15713-7801, UF-15713-4800A, UF-15713-16148 (Fig. S1L).

*Feeding event occurrence data*: Pattern occurrence.

*Specialization level*: 3, at Rose Creek.

*Host plant*: *Crassidenticulum decurrens* (Chloranthales: Chloranthaceae) (The DT392 also observed on the *Pandemophyllum kvacekii* Upchurch and Dilcher, 1990 (Lauraceae)).

*Inferred herbivore*: An auchenorrhynchan (Hemiptera: Auchenorrhyncha) such as a planthopper or treehopper, or a true bug (Hemiptera: Heteroptera), such as a lace bug, were the probable culprits of this damage. Or scale insect such as mealybug or soft scale.

*Modern ecological analog*: The oak lace bug *Corythuca* *arcuata* (Say) (Hemiptera: Tingidae) on an undetermined species of oak, *Quercus* sp. (Fagaceae), from Ohio, U.S.A.

*Remarks*: One or more rows of DT392 punctures occurred exclusively on the major veins of *Crassidenticulum decurrens*, indicating a specialized interaction with a small, phloem-feeding insect such as a planthopper or treehopper that possessed stylate mouthparts (Labandeira, 2019). This DT (piercing and sucking on the major vein of *Pandemophyllum kvacekii*) was previously documented (Xiao et al., 2021b), revealing a scale insect associated with an early angiosperm of the Dakota Formation. The DT392 damage type was observed on un-recorded slab of 15713-16148, which harbors the smaller than 0.5 cm^2^ leaf erea.

**(d) DT234 (Mining)**

*Description*: A medium to long, prominent, curvilinear to sinuate to intestiniform, serpentine mine that avoids primary and secondary veins; the mines have dark, lateral borders, and widths from 0.2 mm in the early stages to 1.3 mm in the latest stage; the frass trail is particulate, displays changes related to instar molts, ranges from gently undulose in early stages to moderately undulate in middle stages to intestiniform in later stages, and ends in a distinctive string of about eight rather large pellets for the last mine stage, each pellet separated by gaps, and followed by a frass-free elliptical chamber 6.5 mm in length.

*Figured specimens*: UF-15713-5400 (Fig. 4I, 4J), UF-15713-16137 (Fig. S1A, S1D), UF-15713-16142 (Fig. S1J, S1K), UF-15713-16144 (Fig. S2G), UF-15713-16098 (Fig. S2L)*,* UF-15713-16140 (Fig. S3D), UF-15713-16145 (Fig. S4C, S4D), UF-15713-12708 (Fig. S5F), UF-15713-16135 (Fig. S9D-S9E), UF-15713-16196 (Fig. S9F), UF-15713-16198 (Fig. S11B, S11C), UF-15713-12724 (Fig. S11I), UF-15713-16138 (Fig. S13B), UF-15713-16134 (Fig. S13C), UF-15713-12716 (Fig. S13D, S13E), UF-15713-16200-2 (Fig. S14F), UF-15713-16126 (Fig. S15J).

*Other material*: UF-15713-8304.

*Feeding event occurrence data*: Single occurrence.

*Specialization level*: 1, at Rose Creek.

*Host plant*: *Pandemophyllum kvacekii* Upchurch and Dilcher, 1990 (cf. Lauraceae)

*Inferred herbivore*: A *Stigmella* or closely related genus of pygmy leafmining moths (Lepidoptera: Nepticulidae). See Labandeira et al. (1994) and Doorenweerd et al. (2015).

*Modern ecological analog*: The pygmy leafmining moth *Stigmella* *speciosa* Frey (Lepidoptera: Nepticulidae) on maple, *Acer* *pseudoplatanus* L. (Sapindaceae), from the United Kingdom.

*Remarks*: DT234 is the most abundant and polyphagous leaf miner of the Rose Creek plant assemblage from the Dakota Formation. This conspicuous *Stigmella* leaf mine, with distinctive frass of the penultimate larval instar and a broad terminal chamber (Doorenweerd et al., 2015), surprisingly lacks plant host specialists. As modern *Stigmella* are overwhelmingly host specialists, this suggests that host specialization is a derived feature in *Stigmella* and possibly other Nepticulidae. DT234 also has been described from localities of the Dakota Formation other than Rose Creek (Labandeira et al., 1994).

**(e) DT386 (Galling)**

*Description*: An isolated, spheroidal to broadly ellipsoidal, foliar gall that avoids primary and secondary veins, often in clusters, and marked by a carbonized covering shed at maturity but preserved at the pathogen center and margin; the mature gall has a surface of 25 to 30 prominent protuberances, each 0.25 mm in diameter circularly arranged about the central chamber; the central chamber is spheroidal, 0.6 to 0.8 mm in diameter and surrounded by a thin, black carbon ring along its outer aspect; the nutritive zone lacks details, often hidden, and is 0.5 to 0.8 mm thick; the outer wall is carbonized and 0.2 mm thick. Colloquially known as the “golf-ball gall”.

*Figured specimens*: UF-15713-12700 (Fig. 2J, 2K), UF-15713-12687 (Fig. 3C, 3E, 5B, 5C), UF-UF-15713-16100 (Fig. 5F, 5G), UF-15713-16131 (Fig. S10E-S10F).

*Other material*: UF-15713-28915, UF-15713-6060, UF-15713-7855, UF-15713-12996, UF-15713-62665, UF-15713-5615, UF-15713-4795, UF-15713-16131, UF-15713-28915.

*Feeding event occurrence data*: Single occurrence.

*Specialization level*: 2, at Rose Creek.

*Host plant*: *Crassidenticulum decurrens* (Chloranthales: Chloranthaceae).

*Inferred herbivore*: Unknown gall maker, possibly a gall wasp or less likely an ascomycotan epiphyllous fungus.

*Modern ecological analog*: The oak apple galler, *Amphibolips* sp. (Hymenoptera: Cynipidae), on northern pin oak, *Quercus* *elipsoidalis* E.J. Hill (Fagaceae), from Georgia, U.S.A.

*Remarks*: This distinctive gall has been referred to as the golfball gall, because of its resemblance to a golf ball from its regular placement of surface bumps and dimples. Although attributable to a gall wasp, gall wasps are not known to occur during the Cretaceous. DT386 also resembles the fruiting structure of certain ascomycotan epiphyllous fungi, although the resemblance is inexact.

**(f) DT398 (Galling)**

*Description*: Blister gall. Equant, foliar galls from 4 to 5 mm in longest dimension that houses a featureless, central, circular chamber approximately 1.5 mm in diameter with 0.1 mm thick wall, surrounded by an extensive, dark flange that projects as several, up to ten, angulate, wispy extensions into intersecting tertiary veins, providing an overall star-shaped appearance, but lacking a discernable outer wall; occurring along the margin and central region of leaves but avoiding the midvein.

*Figured specimens*: UF-15713-12722 (Fig. S9G, S9H), UF-16197-17 (Fig. S15A, S15B).

*Feeding event occurrence data*: Single occurrence.

*Specialization level*: 2, at Rose Creek.

*Host plant*: *Pandemophyllum kvacekii* Upchurch and Dilcher, 1990 (cf. Lauraceae).

*Inferred herbivore*: Probably a cecidomyiid gall midge.

*Modern ecological analog*: The blister gall of *Meunierella* sp. (Diptera: Cecidomyiidae), on common greenbrier, *Smilax rotundifolia* L., (Smilacaceae), from Caroline County, Maryland, U.S.A.

*Remarks*: This gall is similar to DT205, but DT398 lacks a distinctive outer wall and has a smaller central chamber. This blister gall also has been found on “*Sassafras*” *potomacensis* (Lauraceae), from Stump Neck, Maryland, in the two to three million-year-older Patapsco Formation. This suggests a persistence on lauraceous hosts minimally for a few million years.

**(g) DT160 (Borings)**

*Description*: Straight to slightly curved tunnels in wood and bark of tree trunks that are three-dimensionally oriented both perpendicular and parallel to trunk outer surface; each boring 2 to 4 mm in diameter, circular to slightly elliptical in cross section, oriented generally along the xylary grain; with contents of apparently finely macerated material, lacking internal structure but often with fine striae occurring on the surface; occurring in compressed, substantial, woody trunks and branches.

*Figured specimens*: UF-15713-16193 (Fig. S14G-S14I), UF-15713-16132 (Fig. S15H).

*Feeding event occurrence data*: Single occurrence.

*Specialization level*: 1, at Rose Creek.

*Host plant*: An unknown woody axis.

*Inferred herbivore*: The larva of probably an early longhorn beetle (Coleoptera: Cerambycidae).

*Modern ecological analog*: Larvae of the poplar borer *Saperda* *calcarata* Say (Coleoptera: Cerambycidae) on aspen or poplar, *Populus* sp. (Salicaceae), from Minnesota, U.S.A.

*Remarks*: Well preserved borings are rare in compression deposits and typically occur in silica or carbonate permineralized wood. The borings of DT160 on this large, unidentified twig, probably of a shrubby dicot, uniquely preserves single tunnels in longitudinal, oblique and transverse section, allowing three-dimensional reconstruction of this distinctive boring.

**(h) DT174 (Pathogens)**

*Description*: Circular, ovate to broad-elliptical blotch 7 to 9 mm in major diameter on foliage, consisting of an extensive field of dark, necrotized or other altered tissue enveloped by a black, 0.2 to 0.3 mm thick reaction front, often of better defined, thickened, callus-like tissue; the necrotic tissue zone surrounding an inner, concentric, circular core 1 to 2 mm in diameter consisting of an outer lightly hued area and an innermost, flat, dark area in immature blotches, maturing to a thick, central fructification; blotches typically avoid primary and secondary veins and occur throughout the leaf blade except at the margins.

*Figured specimens*: UF-15713-7247 (Fig. 4D, 4E), UF-15713-12677 (Fig. 5K, 5L).

*Other material*: UF-15713-8304.

*Feeding event occurrence data*: Single occurrence.

*Specialization level*: 3, at Rose Creek.

*Host plant*: *Crassidenticulum decurrens* (Chloranthales: Chloranthaceae).

*Inferred culprit*: A basidiomycete rust of the Phragmidiaceae such as *Phragmidium*, containing at maturity a central, noticeable fructification. A lesser possibility is a basidiomycete of the Pucciniaceae such as *Gymnosporangium*.

*Modern ecological analog*: The basidiomycotan fungus Bramble Leaf Rust, *Phragmidium* *violaceum* (Schultz) Winter (Uredinales: Phragmidiaceae) on Himalayan blackberry, *Rubus* *armeniacus* Focke (Rosaceae), from Oregon, U.S.A.

*Remarks*: All known occurrences of DT174 were hosted by early angiosperms, such as *Crassidenticulum decurrens*. When mature, this pathogen forms a circular to broadly elliptical splotch of necrotic tissue that supports a central fructification such as an aecium or telium, that is surrounded by what appears to be a hardened reaction front. Consequently, DT174 is one of the most distinctive epiphyllous fungi in the fossil record (Labandeira and Prevec, 2014).

**(i) DT242** **(Pathogens)**

*Description*: Broadly ovoidal to narrowly elliptical, epiphyllous, isolated to connected splotches oriented along secondary venation, originating as angulate expansions along or at the leaf midrib or primary vein and expanding outward to the margin; each necrotic area darkened, with a bumpy surface, a thick, well-developed bordering reaction front, and an occasional dark, centrally located, prominence representing a fructification; occurrences can reach the leaf margin and be present on both sides of a midvein.

*Figured specimen*: UF-15713-16100 (Fig. 5F)

*Other material*: UF-15709-4820, UF-15713-16138.

*Feeding event occurrence data*: Single occurrence.

*Specialization level*: 2, at Rose Creek.

*Host plant*: *Crassidenticulum decurrens* (Chloranthales: Chloranthaceae)

*Inferred culprit*: A leaf spot fungus such as *Cercospora* Fresen., attributable to an epiphyllous ascomycotan.

*Modern ecological analog*: *Cercospora* Leaf Spot from the epiphyllous ascomycotan, *Cercospora* sp. (Capnodiales: Mycosphaerellaceae) on privet, *Ligustrum* sp. (Oleaceae), from Massachusetts, U.S.A.

*Remarks*: Some mature epiphyllous fungal splotches can have centrally located dark, fructifications that probably are ascocarps, whereas immature splotches lack any evidence of fructifications.

**(j) DT381 (Pathogens)**

*Description*: Dark, typically circular, trapezoidal to polylobate necrotic blotches on foliage, mostly with linear and angulate margins, but occasionally with rounded eggs, and delimited by primary to tertiary, veins and by leaf margins; patch size variable, but from 3 to 4 mm parallel to and 7 to 10 mm perpendicular to leaf axis; occurring throughout the leaf and imparting an overall mottled appearance of light unaffected areas and dark necrotized tissue; the intensity of the color increasing toward adjacent veins.

*Figured specimens*: UF-15713-7516 (Fig. 2G), UF-15713-16098 (Fig. S3H), UF-15713-16123 (Fig. S4F), UF-15713-16215 (Fig. S6F), UF-15713-16101 (Fig. S8E), UF-15713-16206 (Fig. S8F), UF-15713-16178 (Fig. S8G), UF-15713-7835 (Fig. S8H). UF-15713-5401 (Fig. S11G), UF-15713-16099 (Fig. S12F), UF-15713-16118 (Fig. S13G, S13H).

*Other material*: UF-15713-16196.

*Feeding event occurrence data*: Single occurrence.

*Specialization level*: 1, at Rose Creek .

*Host plant*: *Reynoldsiophyllum nebrascense* Upchurch and Dilcher, 1990 (Magnoliidae: family uncertain).

*Inferred culprit*: Most likely a *Mycosphaerella* leaf spot or closely related epiphyllous fungus.

*Modern ecological analog*: The ascomycotan Early Leaf Spot of *Mycosphaerella arachidis* Deighton (Capnodiales: Mycosphaerellaceae) on peanut, *Arachis* *hypogaea* L. (Fabaceae) from India.

*Remarks*: The damage of the DT381 pathogen was widespread on Rose Creek angiosperms and highly polyphagous, infesting the five higher-plant taxa of Austrobaileyales, Chloranthales, Laurales, Eurosidae and unplaced Magnoliidae, with an apparent center of frequency among the several species of Laurales.

**(k) DT382 (Pathogens)**

*Description*: Darkened, typically ovate, broadly elliptical, circular to rarely more elongate, compact, necrotic splotches on foliage, with rounded margins; occurring in a curvilinear row along the leaf margin, and avoiding primary to tertiary veins; each blotch mostly 1 mm in diameter, rarely larger and defined by a wide, dark and conspicuous 0.1 to 0.2 mm wide, encircling and apparently thick reaction front; overall sparsely distributed with splotches dispersed more or less evenly throughout the leaf.

*Figured specimens*: UF-15713-7516 (Fig. 2F, 2G), UF-15713-16100 (Fig. 5I, 5J), UF-15713-16199 (Fig. S12B, S12C).

*Other material*: UF-15713-16216, UF-15713-16150, UF-15713-6144, UF-15713-16108, UF-15713-16160, UF-15713-16163, UF-15713-16115, UF-15713-16199, UF-15713-16105.

*Feeding event occurrence data*: Single occurrence.

*Specialization level*: 1, at Rose Creek.

*Host plant*: *Crassidenticulum decurrens* (Chloranthales: Chloranthaceae).

*Inferred culprit*: A pleosporalean, compact, epiphyllous fungus similar to modern Olive Peacock Spot.

*Modern ecological analog*: The deuteromycotan Olive Peacock Spot, *Venturia oleaginea* (Castagne) Rossman & Crous (Pleosporales: Venturiaceae) on olive, *Olea* *europaea* L. (Oleaceae), from Kerala, India.

*Remarks*: The pathogen DT382 consists of various necrotic blotches with angulate margins along veins on foliage. It was once considered a gall because of its outer wall-like feature that is better interpreted as a hardened reaction front. As well, there is no indication of a chamber in DT382, nor of a fruiting body, indicating it likely is an imperfect (deuteromycotan) fungus.

**(l) DT385 (Pathogens)**

*Description*: Massive swaths of foliar areas containing dark areas of necrotized tissue up to 8 mm by 10 mm or more in dimensions, often occupying major sections of the leaf and occasionally covering most of the leaf surface and surrounded by lighter hued, unaffected leaf tissue; the necrotized areas delimited by very dark to black, distinctive, 0.05 to 1.0 mm thick reaction fronts that also merge or bifurcate, entering the central areas of the necrotic zones and not controlled by veins; the inner necrotized area pockmarked by very small dark dots ca. 0.01 mm in diameter.

*Figured specimen*: UF-15713-12713 (Fig. S6A, S6B).

*Other material*: UF-15713-12698, UF-15713-28926, UF-15713-4938, UF-15713-78181, UF-15713-16143, UF-15713-7807, UF-15713-16164.

*Feeding event occurrence data*: Single occurrence.

*Specialization level*: 2, at Rose Creek .

*Host plant*: *Pabiania variloba* (Laurales, cf. Atherospermataceae, Gormotegaceae, Gyrocarpaceae, Lauraceae and Hernandiaceae).

*Inferred culprit*: An ascomycete such as Black Spot Disease (Helotiales: Dermateaceae) or a closely related epiphyllous fungus.

*Modern ecological analog*: Pierce’s disease, caused by the bacterium *Xylella* *fastidiosa* Wells et al. (Gammaproteobacteria: Xanthomonadaceae), transmitted by the glassy-winged sharpshooter, *Homalodisca* *vitreipennis* Germar (Hemiptera: Cicadellidae), on its host of cultivated grape, *Vitis* *vinifera* L. (Vitaceae), from California, U.S.A.

*Remarks*: DT385 is highly restricted to two hosts of the Chloranthaceae: *Crassidenticulum* *decurrens* and *Densinervum* *kaulii*. DT385 also is one of the few pathogens that likely is represented by a bacterium, rather than a fungus. (The “fingerprint pattern” of DT23 is attributed to a viral blight.) The attribution of DT385 to Pierce’s Disease is notable as this bacterial blight devastated cultivated wine grapes, and consequently the wine industry, in Western Europe during the latter half of the twentieth century, and similarly the disease infected California wine grapes during the period from 1990 to 2010, having a very negative effect on the California wine industry as well.

**(m) DT387 (Pathogens)**

*Description*: Overall circular, ovate to broadly elliptical, very dark splotches with undulose margins on foliage; consisting of a distinct but not thickened outer border, encompassing a necrotized area, in turn surrounding a more lightly hued circular area possibly representing a ring-like depression; in turn, enclosing eight to ten radially deployed, wedge-like structures that represent aecia or other fungal fructifications in immature specimens, becoming loosened in mature specimens and irregularly distributed across the splotch center; the splotches avoiding significant veins and lacking a noticeable, circumscribing reaction front.

*Figured specimens*: UF-15713-12650 (Fig. 6A-6C), UF-15713-12696 (Fig. 6D-6F), UF-15713-12688 (Fig. 6I). UF-15713-6074 (Fig. 6L), UF-15713-16191 (Fig. S5A, S5C-S5E).

*Other material*: UF-15713-12687, UF-15713-16126, UF-15713-12651, UF-15713-12683, UF-15713-4746, UF-15713-7825, UF-15713-4953, UF-15713-28920, UF-15713-28933, UF-15713-28935.

*Feeding event occurrence data*: Single occurrence.

*Specialization level*: 2, at Rose Creek.

*Host plant*: *Crassidenticulum decurrens* (Chloranthales: Chloranthaceae).

*Inferred herbivore*: Most likely a puccinaceous rust with prominent aecia, such as *Gymnosporangium*.

*Modern ecological analog*: The basidiomycotan aecia of Pear Rust, *Gymnosporangium* *sabinae* (Dicks) Oerst. (Pucciniales: Puccinaceae) on pear, *Pyrus* sp. (Rosaceae), from an unknown locality.

*Remarks*: The rust of DT387 is a very distinctive epiphyllous fungus supporting prominent fructifications of aecia that are arrayed in a circular fashion around the center of a mature necrosis. The aecia appear to be solid vertical structures in life but often are preserved as reclining on their sides at various angles.

**(n) DT388 (Pathogens)**

*Description*: Very dark to black circular, ovate, broadly ellipsoidal to polylobate splotches, occasionally with bud-like circular extensions, present on foliage and position controlled by primary to tertiary veins; each splotch ranging from 1 to 2.5 mm in maximum dimension, containing a 0.05 to 0.1 mm thick, upraised outer rim, and a featureless, flat, black, inner area that bears several miniscule, white, circular flecks of unknown origin; the splotches sometimes occurring in rows on the leaf, often paralleling the leaf edge; no indication of fungal fructifications are present.

*Figured specimens*: UF-15713-28918 (Fig. 3H), UF-15713-16137 (Fig. S1A, S1B), UF-15713-16145 (Fig. S4C, S4E), UF-15713-16123 (Fig. S4F-S4H), UF-15713-16098 (Fig. S4I), UF-15713-4802 (Fig. S9J), UF-15713-16193 (Fig. S11F), UF-15713-5401 (Fig. S11G, S11H), UF-15713-16196-13 (Fig. S15E-S15G).

*Other material*: UF-15713-16101, UF-15713-12722, UF-15713-16118, UF-15713-7849, UF-15713-16193, UF-15713-16221, UF-15713-4450, UF-15713-7844, UF-15713-7853, UF-15713-12965.

*Feeding event occurrence data*: Single occurrence.

*Specialization level*: 1, at Rose Creek.

*Host plant*: *Anisodromum wolfei* Upchurch and Dilcher (Magnoliales: unplaced family).

*Inferred herbivore*: An ascomycotan, epiphyllous fungus of the Mycosphaerellaceae.

*Modern ecological analog*: The ascomyotan Late Leaf Spot, *Mycosphaerella berkeleyi* W.A. Jenkins (Capnodiales: Mycosphaerellaceae), on peanut, *Arachis* *hypogaea* L. (Fabaceae) from Virginia, U.S.A.

*Remarks*: Pathogen DT388 is the second most commonly occurring pathogen on the Rose Creek plant assemblage, and is widespread on Chloranthales, Laurales and Eurosidae. DT388 and other pathogen DTs, with the exception of DT385, indicate that pathogens of the Rose Creek plant assemblage are tracking the most abundant taxa rather than targeting particular taxa.

**Appendix S19: Herbivorized surface area comparisons. Rose Creek (A) compared to equivalent data from fossil (B) and modern (C–I) studies of individual species and bulk floras.**

Botanical source of herbivory data *n*^1^ Herbiv. index^2^ Locality Habitat Reference

**Fossil Herbivory Data**

**A. Rose Creek plant assemblage (Albian)** Rose Creek, Fairbury, Coastal shrubland of a This study

Herbivore component communities Jefferson Co.; Nebraska, mangrove swamp

*Crassidenticulum* *decurrens* (Chloranthales) 248 ***6.00*** USA

*Pandemophyllum* *kvacekii* (Laurales) 244 ***3.41***

*Pabiania* *variloba* (Laurales) 239 ***4.92***

Angiosperms only (vegetative occurrences) 1076 ***3.52***

Rose Creek bulk plant assemblage 2084 ***3.14***

**B. Lost Valley plant assemblage (Campanian)** Lost valley locality, Grand Humid subtropical Maccracken

*Catula* *gettyi* Maccracken, Miller, Johnson 156 ***2.10***, 1.36– Staircase–Escalante coastal woodland or et al., 2021

Sertich & Labandeira (Lauraceae) 3.03^3^ National Monument, near forest

Escalante, southern Utah

**Modern Herbivory Data**

**C. *Connarus* *turczaninowii* Triana & Planch. (Oxalidales)** Barro Colorado Island, Tropical Aide and

Total damage for mature leaves, year 1982 263 ***2.81*** ± 0.31 Republic of Panamá forest Zimmerman,

Total damage for mature leaves, year 1983 258 ***6.02*** ± 0.61 1990

Total damage for mature leaves, year 1984 253 ***3.95*** ± 0.38

**D. *Avicennia* *marina* (Forssk.) Vierh. (Lamiales)** Gordon Creek and Mangrove shrubland Burrows,

Gordon Creek, upper canopy 250 ***5.95*** ± 0.60 Saunders Beach, near 2003

Gordon Creek, lower canopy 250 ***8.45*** ± 0.91 Townsville, Queens-

Saunders Beach, upper canopy 250 ***5.90*** ± 0.67 land, Australia

Saunders Beach, lower canopy 250 ***6.75*** ± 0.91

***Rhizophora* *stylosa* Griff. (Malpighiales)**

Gordon Creek, upper canopy 250 ***2.11*** ± 0.35

Gordon Creek, lower canopy 250 ***3.81*** ± 0.67

Saunders Creek, upper canopy 250 ***2.68*** ± 0.37

Saunders Creek, lower canopy 250 ***4.23*** ± 0.63

**E. *Doryphora* *sassafras* Endl. (Laurales)** Dorrigo National Park and Rain forest Lowman,

Dorrigo Natl. Park, shade leaves at 2 m 30 ***9.3***, 2.3 sem^4^ New England National Park, 1987

Dorrigo Natl. Park, sun leaves at 3 m 30 ***1.2***, 0.6 sem New South Wales, Australia

New England Natl. Park, sun leaves at 5m 30 ***13.9***, 3.1 sem

New England Natl. Park, shade leaves at 2 m 30 **7.3**, 2.3 sem

**F. *Croton* *pseudoniveus* Lundell (Malpighiales)** Estación de Biología Tropical deciduous Filip et al.,

Discrete measurement 100–500^5^ ***16.98***, n. s.^6^ Chamela; Jalisco, México forest 1995

Long-term measurement 100–500 ***19.74*** México

*Lonchocarpus* *eriocarinalis* (Fabales)

Discrete measurement 100–500 ***4.93***, n. s.

Long-term measurement 100–500 ***4.14***

*Cordia* *alliodora* (Boraginales)

Discrete measurement 100–500 ***2.61***, n. s.

Long-term measurement 100–500 ***2.48***

*Trichilia* *trifolia* (Sapindales)

Discrete measurement 100–500 ***2.30***, n. s.

Long-term measurement 100–500 ***1.95***

*Bursera* *heteresthes* (Sapindales)

Discrete measurement 100–500 ***1.56***, n. s.

Long-term measurement 100–500 ***1.24***

**G. Warm-temperate bulk floras**^7^ Duke Forest Free-Air Warm-temperate Hamilton

Total damage, June 1999, ambient CO_2_^8^ 243^9^ ***3.1*** Concentration Facility; near deciduous forest et al., 2004

Total damage, June 1999, elevated CO_2_ 243 ***2.9*** Chapel Hill, North Carolina,

Total damage, July 1999, ambient CO_2_ 243 ***5.4*** USA

Total damage, July 1999, elevated CO_2_ 243 ***2.9***

Total damage, Sept 1999, ambient CO_2_ 243 ***6.8***

Total damage, Sept 1999, elevated CO_2_ 243 ***5.0***

Total damage, July 2000, ambient CO_2_ 648 ***5.5***

Total damage, July 2000, elevated CO_2_ 648 ***3.2***

**H.** **Tropical, arborescent** **species**^10^

Years 1998, 1999 and 2000^11^ 300,298,300 ***9.5*** ± 0.003^12^ La Selva Heredia Province, Tropical lowland wet Smith and

Costa Rica forest Nufio, 2003

Years 1998, 1999 and 200^11^ 300,300,199 ***4.9*** ± 0.003^12^ Parque Nacional Corcovado Tropical lowland wet Smith and

Corcovado, Puntarenas forest Nufio, 2003

Province, Costa Rica

**I. DT richness of 22 tropical, arborescent species**^13^

Study of DT and their insect herbivore richness 8.8 DTs^14^ Área Protegida de San Seasonally dry, tropical Carvalho et

Lorenzo, Colón Prov., lowland forest al., 2014

Panamá; and Parque

Metropoliano, Panamá

Oeste Prov., Panamá

1. The designation, *n*, is the number of leaves examined.

2. The herbivory index is the leaf area removed due to arthropod herbivory.

3. Reported as a confidence interval.

4. Reported as the standard error of the mean.

5. The number of leaves examined for each analysis was reported as between 100 to 500.

6. The designation, n. s., indicates that the herbivory values for each discrete and long-term pair were not significant.

7. The four species examined are *Liquidambar* *styraciflua* L. (Saxifragales), *Acer* *rubrum* L. 1753 (Sapindales), *Ulmus* *alata* Michx. (Rosales), and *Cercis* *canadensis* L. (Fabales).

8. Ambient CO_2_ levels are 366 μl l^-1^; elevated CO_2_ levels are 616 μl ^-1^.

9. The number of leaves, *n*, was not directly reported, and determined by a qualitative description for obtaining leaves in the methods section.

10. Leaf taxa were not reported.

11. Years at left match the numbers of leaves (*n*) examined at right

12. Range data for La Selva leaves: 8.8–10.2 percent; for Corcovado leaves: 4.4–5.4 percent.

13. The 22 plant species examined are *Anacardium* *excelsum* L. (Kunth) Skeels (Anacardiaceae), *Astronium* *graveolens* Jacq. (Anacardiaceae), *Bonamia* *trichantha* Hallier (Convolvulaceae), *Castilla* *peltata* L. (Moraceae), *Cecropia* *peltata* L. (Cecropiaceae), *Cordia* *alliodora* (Ruiz & Pav.) Oken (Boraginaceae), *Enterolobium* *cyclocarpum* (Jacq.) Griesb. (Fabaceae), *Ficus* *insipida* Willd. (Moraceae), *Luehea* *seemannii* Triana & Planch. (Malvaceae), *Pseudobombax* *septenatum* (Jacq.) Dugand (Malvaceae), *Spondias* *mombin* L. (Anacardiaceae), *Vitis* *tiliacea* (Kunth) Hemsl. (Vitaceae), *Aspidosperma* *spruceanum* Benth. ex Mull. Arg (Apocynaceae), *Brosimum* *utile* (Kunth) Oken ex J. Presl (Moraceae), *Calophyllum* *longifolium* Willd. (Calophyllaceae), *Cordia* *bicolor* A. DC (Boraginaceae), *Dussia* sp. (Fabaceae), *Guatteria* *dumetorum* R.E. Fr. (Annonaceae), *Jacaranda* *copaia* (Aubl.) D. Don (Bignoniaceae), *Manilkara* *bidentata* (A. DC.) A. Chev. (Sapotaceae), and *Tapirira* *guianensis* Aubl. (Anacardiaceae).

14. An average of 22 species with a DT range of 3–15.

**Appendix S20:** **Reasons for elevated herbivory levels at Rose Creek**

**(a) Methodological issues**

The answer lies in two issues. The first issue is methodological and is associated with recent data improvements of the existing version of the fossil arthropod *Damage Guide* (Labandeira et al., 2007) and subsequently published new DT descriptions. There are five reasons explaining why the distribution of DTs on plants at Rose Creek are considerably elevated when compared to other earlier and later quantified floras (Appendix S21). Notably, 41 of the 114 DTs can be attributed to increased categorization and expansion of the number of DTs for non-dicot vascular plants in the present, working version of the *Damage Guide* (Appendix S21). Fourteen examples are detailed in Appendix S17. The first reason is establishment of two new piercing-and-sucking DTs that record behavioral patterns of new puncture trails that record curvilinear, looping, clustered and other patterns based on the feeding patterns of modern hemipteroid insects. A second reason for the abundance of DTs at Rose Creek is the description seven new DTs that document distinctive patterns of damage from pathogens that formerly were not included or relegated to the default category of DT58. Third, is the identification of nine DTs on unidentifiable foliar elements that were not present on identifiable leaves of the flora at large and typically not included in the DT spectrum in studies of angiosperm floras. A fourth reason is the presence of seven DTs on unattributed stems and other axes that normally would not be included in studies of angiosperm floras. Last, the Rose Creek plant assemblage contained 43 specimens of flowers, of which *Dakotanthus* *cordiformis* harbored three DTs, two of which were new to Rose Creek. It was important that documentation of all DTs in the Rose Creek plant assemblage, previously established and new (Appendix S4, S10) (Xiao et al., 2021a), capture the total richness of arthropod associations with all plant organs during an early phase of angiosperm diversification.

**(b) Intrinsic elevated herbivory richness**

The second issue is that the elevated richness of herbivory at Rose Creek is real and intrinsic, and it records an expansion of arthropod herbivores and pathogens as they partitioned angiosperm tissues and taxa early during their diversification, as predicted by other studies (McKenna et al., 2009; Wahlberg et al., 2013; Isaka and Sato, 2015; Fagua et al., 2017). The 114 DTs of the Rose Creek plant assemblage could be “standardized” by subtracting the 40 DTs that would not be included in quantitative analyses of dicot leaves from a typical Cretaceous–Paleogene boundary interval or from Cenozoic plant assemblages. The resulting DT richness is 74, which is still elevated by comparison to Cenozoic floras. Other types of evidence, such as component community (Figs. 8–10) structure and host-plant specialization levels (Appendix S15), indicate elevated levels of arthropod herbivore richness at Rose Creek.

**Appendix S21: Five methodological reasons for elevated damage type (DT) diversity at Rose Creek**

Functional

Reason and Number of Added DTs Feeding Groups Damage Types^1^ Distribution on Plants .

**1**. More finely defined piercing and sucking DTs Piercing and Sucking DT138, DT184, DT281, DT330, DT281, DT330, DT338, and DT383

based on known behaviors of hemipteroid insects DT338, DT344, DT358, **DT383** developed on many^2^ plant hosts;

such as thrips, planthoppers and true bugs that **DT392**, **DT402**; scale insect DT138, DT184, DT344, DT358,

produce curvilinear, looped and clustered puncture **DT384**, **DT394** and **DT406^2^** DT384, DT392 DT394, DT402 and

tracks. Also, two new scale-insect DTs. **13** added. DT406 on a few plant hosts.

**2**. Establishment of distinctive pathogen types not Pathogen DT66, DT69, DT174, DT242, DT69, DT242, DT261, DT381,

recognized in earlier studies. Previously, pathogens DT261, **DT381**, **DT382**, **DT385**, DT382 and DT388 on many^2^ plant

often were not treated or assigned to the default **DT387**, **DT388** hosts; DT66, DT174, DT385 and

category of DT58. **10** added. DT387 on a few plant hosts.

**3**. The presence of DTs on unidentifiable foliage Skeletonization DT56 All of these DTs are on a few^2^

elements that are not present on other Mining DT92, DT141, DT288 plant hosts.

Identifiable elements of the flora. **9** added. Galling DT85, DT265, DT266, DT289,

DT303

**4**. The presence of DTs on various unidentifiable Oviposition: DT226, DT331 All of these DTs are rare to very

stems and axes that do not occur on identifiable Galling: DT87, DT188 rare on plant hosts.

elements of the flora. **7** added. Seed Predation: DT74

Wood boring: DT160, DT284

**5**. Establishment of small, delicate DTs on flowers, Margin Feeding **DT405**^2^ Both of these DTs are on the

particularly petals, a plant organ not previously Piercing and Sucking **DT402**^2^ same plant host.

damage typed. **2** added, but not counted in

this analysis (see Xiao et al., 2021a).

1. Damage types in bold designate new DTs for Rose Creek (see Appendix S5); seventeen of these DTs are new for the Rose Creek plant assemblage.

2. The scale insect DT384, DT392, DT394 and DT406 were initially described at Xiao et al., 2021b; DT405 and DT402 were initially described at Xiao et al., 2021a.

**Appendix S22: The persistence of herbivore component communities**

Herbivore component communities have been reconstructed for a variety of arborescent plants in the fossil record (Labandeira, 1998; Slater et al., 2012; Labandeira et al., 2016; Xiao et al., 2022). In a relevant context, Futuyma and Mitter (1996) mentioned the persistence of certain lepidopteran leaf mines at the genus level on Platanaceae from the Dakota Formation (Labandeira et al., 1994) as constituting conservatism in the evolution of herbivore component communities. Most of these leaf-miner–host relationships subsequently have been confirmed (Doorenweerd et al., 2015), indicating that, based on the evidence from certain leaf mining lineages, that component communities may evolve intact, although persistence may be linked to shifts, or lack thereof, of host specialization by the leaf miners (see below). However, in a study of extant herbivores on bracken (*Pteridium* *aquilinum* (L.) Kuhn), a widely distributed fern occurring globally on major landmasses, results indicate considerable variability in the number of insect and possibly mite species (Appendix S23), occupying major FFGs (their “herbivore guilds”) on this plant (Lawton, 1978, 1982, 1984). This suggests that there are significant differences in the ecological structure in ecological space, and likely in evolutionary time as well (Lawton, 1984; Lawton and Gaston, 1989).

**Appendix S23: Documentation for Figure 12.** **Herbivore component communities of five modern plant hosts^1^**^–4^

Host plant, locality_ External feeders Piercer & suckers Miners Gallers Borers Seed predators Root feeders References .

***Pteridium aquilinum*** *Aneugmenus Aphis* *fabae*, *Chirosia* *nigripes*, *Dasineura* **0** **0** *Phymatopus*  Rigby and Lawton,

L. (Kuhn) (Polypodi- *fuerstenbergensis*, *Bryocoris* *pteridis*, *Chirosia* *crassiseta*, *pteridicola*  *hecta,* 1981; Lawton,

Iales): bracken; *Aneugmenus padi*, *Ditropis pteridis*, *Chirosia* *histricina*, *Dasineura* *Phymatopus*  1976, 1982; Gaston

Skipwith Common *Aneugmenus* *Macrosiphon* *Chirosia* *pteridis* *fusconebulosa*, and Lawton, 1988,

North Yorkshire, *temporalis*, *ptericolens*, *grossicauda*, *Phytopus pteridis Phymatopus* 1989; Holy- oak

U.K. *Bourletiella* *Monalocoris filicis*, *Monochroa* **3** *sylvina,* and Lawton, 1992

*viridescens*, *Philaenus* *cytisella*  **3**

*Ceramica* *pisi*, *spumarius* *Phytoliriomyza*

*Euplexia lucipara*, **6** *hilarella*,

*Olethreutes Phytoliriomyza*

*lacunana*, *pteridii*

*Phlogophora* **7**

*meticulosa*,

*Phyllopertha*

*horticola*,

*Petrophora*

*chlorosata*,

*Spilarctia lutea*,

*Stromboceros*

*delicatulus*,

*Strongylogaster*

*multifasciata*,

*Strongylogaster*

*macula*

*Strongylogaster*

*xanthocera*

*Tenthredo*

*ferruginea*,

*Tenthredo* sp. 2

**18**

***Salix* *seriacea*** Marsh *Calligrapha* *Mordwilkoja* *Lithocolletis* sp. *Aculops* *Cryptorhynchus* **0** **0** Roche and Fritz,

***–Salix eriocephala*** *multipunctata*, *vagabunda*, *Phyllonorycter* *tetanothrix*, *lapathi*, 1997; Orians et al.,

Mich**–F_1_–F_2_ hybrid** *Caloptila* sp. 1, *Parthenolecanium salicifoliella,* *Euura Saperda populnea,*  1997; Hochwender

**system**: silky willow *Chrysomela* *knabi*, *corni*, *Phyllocnistis* *salicis*, *Xyleborus* *dispar*  and Fritz, 2004;

–heartleaved willow *Chrysomela* *Phloeomyzus salicicolellia* *Iteomyia* **3** Nordman et al.,

–hybrids; Milford, *scripta*, *passerinii*, **3** *salicifolia*, 2005; Charles et

New York State, Leaf-folding sp. 1, *Tuberolachnus*  *Phyllocolpa*  al., 2014

U. S. A. Leaf-folding sp. 2, *salignus* *eleanorae*,

*Nymphialis* **4** *Phyllocolpa*

*antiopa*, *nigrita*,

*Plagiodera* *Phyllocolpa* sp. 1,

*versicolora*, *Phyllocolpa*

*Popillia japonica*  *terminalis,*

**9** *Rabdophaga rigidae,*

*Rabdophaga*

*salicis,*

*Rabdophaga* sp. G

**10**

***Lepidium* *draba*** L. *Colaphellus* sp., *Bryobia* *praetiosa*, *Plutella* *Ceutorhynchus*  *Ceutorhynchus* Root feed. sp. 1, Cripps et al., 2006

(Brassicales): *Brassicogethes* *Eurydema*, *xylostella*, *assimilis*, *cardariae*, Root feed. sp. 2

peppergrass; *aenus*, *ornatus*, Leaf miner sp. 1, *Ceutorhynchus* *Ceutorhynchus* **2**

Western Europe *Meligethes* *lepidii*, *Frankliniella* Leaf miner sp. 2 *cardariae*, *merkii*,

*Phyllotreta* *atra*, *occidentalis*, **3** Leaf galler sp. 1 *Ceutorhynchus*

*Phyllotreta* *Haplothrips*  **3** *obstrictus*,

*erysimi*, *leucanthemi*, *Ceutorhynchus*

*Phyllotreta* *Lygus* *rugulipennis*, *pallidactylus*,

*nigripes*, *Myzus* *persicae*  *Ceutorhynchus*

*Psylliodes* *wrasei* **6** *turbatus*,

**7**  Seed pred. sp. 1,

Seed pred. sp. 2,

Seed pred. sp. 3,

Seed pred. sp. 4

**9**

***Solanum* *carolinense*** *Anthonomus* *Gargaphia* *solani*, *Tildenia* *georgi*, *Frumenta Frumenta* *Ischnodemus* **0** Pienkowski and

L. (Solanales): Caro- *eugenii*, *Bactericera* *Tildenia nundinella, nundinella, falicus* Kok, 1976; Imura,

lina nightshade; *Anthonomus* *cockerelli*, *inconspicuella Prodiplosis Synanthedon Zonosemata* 2003; Wise, 2007

Eastern U.S.A. *nigrinus*, *Scaphytopius* **2** *longifilia rileyana, electa*

*Diabrotica* *un*- *acutus*, **2** *Trichobaris* **2**

*decimpunctata*, *Tetranychus,* *trinotata*

*Epitrix* *cucumeris*, *urticae*, **3**

*Epitrix hirtipennis,* *Thrips* *tabaci*

*Frumenta* **5**

*nundinella,*

*Gratiana pallidula*,

*Leptinotarsa*

*decemlineata,*

*Leptinotarsa juncta*,

*Manduca*

*quinquemaculata,*

*Manduca sexta*,

**12**

***Asclepias* *syriaca*** L. *Cycnia* *tenera*, *Aphis* asclepiadis, *Liriomyza* *Lestodiplosis* *Cycnia* *tenera*, *Rhyssomatus* Weiss, 1921;

(Gentianales): *Danaus* *plexippus*, *Aphis* *gossypii*, *asclepiadis*  *asclepiae*, *Calomycterus* *lineaticollis*, Dailey et al., 1978;

common milkweed; *Euchaetes* *egle*, *Aphis* *nerii*, *Liriomyza* *Rhopalomyia* *setarius*, *Tetraopes* Price and Wilson,

Jokers Hill, Southern *Gymnetron* *Aphis* *rumicis*, *pusilla*, *gnaphalodis* *Glischrochilus* *femoratus* 1979; Betz et al.,

Ontario, Canada *tetrum*, *Lygaeus* *kalmii*, **2** *Tetranychus* *quadrisignatus*, *Tetraopes* 1997; Van Zandt

*Labidomera* *Macrosiphum* *urticae* *Lygaeus kalmii*, *tetrophthal*- and Agrawal, 2004;

*civicollis*, *rudbeckiae*, **3** *Oncopeltus* *mus* Agrawal, 2005;

*Rhyssomatus* *Myzocallis* *fasciatus,* Züst and Agrawal,

*lineaticollis*, *asclepiadis*, *Otiorhynchus* 2016

*Tetraopes* *Myzus* *persicae*, *ovatus,*

*femoratus* *Oncopeltus* *Sitona* *hispidulus*

*Tetraopes* *fasciatus* **7**

*tetraphthalmus* **9**

**8**

1. The taxa represented for these five modern component communities are core or otherwise highly associated herbivore species with the plant host. The total number of taxa for each functional feeding group of each plant host is provided at the end of each list.

2. Insect and mite herbivore taxa were collected from plant-hosts in their native region and represent longer-term associations than in regions where they have been introduced.

3. Some taxa are listed under multiple functional feeding groups, a consequence of different developmental stages (nymph, larva, adult) of the same species occurring on different plant organs of the plant host. For example, a bug that pierces a stem as a nymph but becomes a seed predator as an adult.

4. Some listed taxa remained unidentified at the time of the cited publication.

**Appendix S24:** **Modern methods of assessing arthropod herbivory**

The application of modern studies of arthropod herbivory to the fossil record requires explanation. Most modern studies of herbivory have collected data that is incompatible with the system of FFGs, and DTs used herein (Labandeira et al., 2007, and subsequent published addenda). Although the FFG is equivalent to the concept of a feeding guild used in modern studies (Lewinsohn et al., 2005), the best analog of the DT is the individual herbivore species creating the damage (Carvalho et al., 2014). DTs are a system of fossil and modern qualitative categories from which quantitative data expressing richness and intensity are derived from (Wilf and Labandeira, 1999). Two alternative ways of measuring herbivory richness, that is, the direct presence or absence of DTs measured on plant specimens, are available to modern studies. The first consists of herbivorized surface area captured by digitized measurement of herbivorized perimeters (Smith and Nufio, 2003), a historically older approach that also is used in this report, or the recent method of using a grid over herbarium specimens from which DTs are randomly sampled (Meineke and Davies, 2018), not used in this report. Nevertheless, the FFG–DT system of assessing herbivory also has been used, sometimes with modification, in several modern studies. Such studies include determination of the spectrum of specialist damage on a single host-plant species (Bachelot and Kobe, 2013), arthropod herbivory on a per-leaf basis for assessing continent-wide latitudinal differences (Adams and Zhang, 2009; Adams et al., 2010), or evaluating herbivory along regional environmental gradients (Sohn et al., 2017). Another use is establishment of correlations between folivore insect diversity and the DT richness that they produce (Carvalho et al., 2014). Nonetheless, most recent studies opt for measurement of surface area, especially for determination of herbivory intensity, often in studies when damage often is difficult to assess solely by categorization methods.

**Appendix S25: Hypotheses explaining modern plant–arthropod herbivory**

Based on modern studies, seven major hypotheses have been presented to explain the mechanism of how plant hosts and their arthropod herbivores interact in time and space. The hypotheses are the growth rate–differentiation hypothesis (Loomis, 1953; Herms and Mattson, 1992), diffuse coevolution or escape-and radiate hypothesis (Ehrlich and Raven, 1964; Futuyma and Keese, 1992), resource concentration hypothesis (Root, 1973; Grez and González, 1995), plant apparency hypothesis (Feeny, 1976; Rhodes and Cates, 1976), environmental constraint hypothesis (Bryant et al., 1983; Tuomi, 1991), resource availability hypothesis (Coley, 1987; Endara and Coley, 2011), and enemy release hypothesis (Keane and Crawley, 2002). Details of these hypotheses will not be considered here; Stamp (2003), Agrawal (2007) and Endara and Coley (2011) provide excellent reviews. Although some of these plant–insect interaction hypotheses are difficult to test in lieu of detailed anatomy of the plant hosts, the hypothesis that accords best with these data is the resource availability hypothesis (Coley, 1987; Endara and Coley, 2011). It maintains that plant species growing in resource rich habitats produce more rapid growth rates and thus such species have shorter leaf lifetimes, less intrinsic physical and chemical defenses, and support higher herbivory rates than species growing in resource poor habitats (also see Wilf et al., 2001). These conditions are consistent with data from the Rose Creek plant assemblage, but because of the widespread evidence for herbivory, there is more support for the presence of plant taxa with rapid growth rates than there is for those with slow growth rates that would experience considerably less herbivory. Rose Creek plants with rapid growth rates and elevated herbivory, such as *Crassidenticulum* and *Pandemophyllum*, are consistent with a woody and weedy life habit in a moist, understory and ruderal environment (Field et al., 2004; Royer et al., 2010). A further test of the resource availability hypothesis would require similar flora-wide analyses for other highly sampled floras from the Dakota Formation, such as Hoisington III, Braun’s Ranch and Linnenberger Brother’s Ranch (Appendix S4) that represent different habitats.

**Supplementary material references**

Adams, J.M., Brusa, A., Soyeong, A., Ainuddin, A.N., 2010. Present-day testing of a paleoecological pattern: Is there really a latitudinal difference in leaf-feeding insect-damage diversity? Review of Palaeobotany and Palynology 162, 63–70.

Adams, J.M., Zhang, Y., 2009. Is there more insect folivory in warmer temperate climates? A latitudinal comparison of insect folivory in eastern North America. Journal of Ecology 97, 933–940.

Agrawal, A.A., 2005. Natural selection on common milkweed (*Asclepias* *syriaca*) by a community of specialized insect herbivores. Evolutionary Ecology Research 7, 651–667.

Agrawal, A., 2007. Macroevolution of plant defense strategies. Trends in Ecology & Evolution 22, 103–109.

Agrios, G.N., 2005. Plant pathology. Fifth edition. Academic Press, San Diego.

Aide, T.M., Zimmerman, J.K., 1990. Patterns of insect herbivory, growth, and survivorship in juveniles of a Neotropical liana. Ecology 71, 1412–1421.

Alekseev, A.C., Dmitriev, V.Y., Ponomarenko, A.G., 2001. The evolution of taxonomic diversity. Geos, Moscow (in Russian).

Álvarez, R.S., González-Sierra, S., Candelas, A., Martínez, J.J.I., 2013. Histological study of galls induced by aphids on leaves of *Ulmus* *minor*: *Tetraneura* *ulmi* induces globose galls and *Eriosoma* *ulmi* induces pseudogalls. Arthropod–Plant Interactions 7, 643–650.

Arens, N.C., Gleason, J.B., 2016. Insect folivory in an angiosperm-dominated flora from the Mid-Cretaceous of Utah, USA. Palaios 31, 71–80.

Bachelot, B, Kobe, R.K., 2013. Rare species advantage? Richness of damage types due to natural enemies increases with species abundance in wet tropical forest. Journal of Ecology 101, 846–856.

Bannister, J.M., Conran, J.G., Lee, D.E. 2016. Life on the phylloplane: Eocene epiphyllous fungi from Pikopiko fossil forest, Southland, New Zealand. New Zealand Journal of Botany 54, 412–432.

Bao, T, Wang, B., Li, J., Dilcher, D.L., 2019. Pollination of Cretaceous flowers. Proceedings of the National Academy of Sciences of the United States of America 116, 24707–24711.

Basinger, J., Dilcher, D.L., 1984. Ancient bisexual flowers. Science 224, 511–513, <https://doi.10.1126/science.224.4648.511>

Beamer, R.H., 1928. Studies on the biology of Kansas Cicadidae. University of Kansas Science Bulletin 18, 155–263.

Beck, A.L., Labandeira, C.C., 1998. Early Permian insect folivory on a gigantopterid-dominated riparian flora from north-central Texas. Palaeogeography, Palaeoclimatology Palaeoecology 142, 137–173.

Bell, C.D., Soltis, D.E., Soltis, P.S., 2010. The age and diversification of the angiosperms re-revisited. American Journal of Botany 97, 1296–1303.

Béthoux, O., Galtier, J., Nel, A., 2004. Earliest evidence of insect endophytic oviposition. Palaios 19, 408–413.

Betz, R.F., Rommel, W.R., Dichtl, J.J., 1997. Insect herbivores of 12 milkweed (*Asclepias*) species. In: Warwick, C. (Ed.), Proceedings of the Fifteenth North American Prairie Conference, 7–19. Natural Areas Association; Bend, Oregon, United States.

Bieńkowski, A.O., 2010a. Feeding behaviour of leaf beetles (Coleoptera, Chrysomelidae). Entomological Review 90, 1–10.

Bieńkowski, A.O., 2010b. Anthophagy in the leaf beetles (Coleoptera, Chrysomelidae). Entomological Review 90, 423–432.

Bryant, J.P., Chapin III, F.S, Reichardt, P.B., Clausen, T., 1983. Response of winter chemical defense in Alaska paper birch and green alder to manipulation of plant carbon/nutrient balance. Oecologia 72, 510–514.

Burrows, D.W., 2003. The role of insect leaf herbivory on the mangroves *Avicennia* *marina* and *Rhizophora* *stylosa*. Dissertation, James Cook University, Townsville, Australia.

Carvalho, M.R., Wilf, P., Barrios, H., Windsor, D.M., Currano, E.D., Labandeira, C.C., Jaramillo, C.A., 2014. Insect leaf-chewing damage tracks herbivore richness in modern and ancient forests. PloS One 9, e94950, <https://doi.org/10.1371/journal.pone.0094950>

Charles, J.G., Nef, L., Allegro, G., Collins, C.M., Delplanque, A., Gimenez, R., Höglund, S., Jiafu, H., S Larsson, Luo, Y., Parra, P., Singh, A.P., Volney, W.J.A., Augustin, S., 2014. Insects and other pests of poplars and willows. In: Isebrands, J.G., Richardson, J. (Eds.), Poplars and Willows: Trees for Society and the Environment, 459–526, pls 33–48. Food and Agricultural Organization of the United Nations and Commonwealth Agricultural Board International, Wallingford, United Kingdom.

Childers, C.C., Achor, D.S., 1991. Feeding and oviposition injury to flowers and developing floral buds of ‘navel’ orange by *Franklinella* *bispinosa* (Thysanoptera: Thripidae) in Florida. Annals of the Entomological Society of America 84, 272–282.

Clissold, F.J., 2007. The biomechanics of chewing and plant fracture: Mechanisms and implications. In: Casas, J., Simpson, S.J. (Eds.), Insect mechanics and control. Advances in Insect Physiology 34, 317–372.

Coley, P.D., 1987. Interspecific variation in plant anti-herbivore properties: the role of habitat quality and rate of disturbance. New Phytologist 106, 251–263.

Collinson, M.E., Hooker, J.J., 1991. Fossil evidence of interactions between plants and plant-eating mammals. Philosophical Transactions of the Royal Society B 333, 197–208.

Condamine, F.L., Clapham, M.E., Kergoat, G.J., 2016. Global patterns of insect diversification: towards a reconciliation of fossil and molecular evidence? Scientific Reports 6, 19208.

Correia, P., Labandeira, C.C., Bashforth, A.R., Šimůnek, Z., Cleal, C.J., Sá, A.A., 2020. The history of herbivory on sphenophytes: a new calamitalean with an insect gall from the upper Pennsylvanian of Portugal and a review of arthropod herbivory on an ancient lineage. International Journal of Plant Science 183, 387–418.

Colwell, R.K., Chao, A., Gotelli, N.J., Lin, S.Y., Mao, C.X., Chazdon, R.L., Longino, J.T., 2012. Models and estimators linking individual-based and sample-based rarefaction, extrapolation and comparison of assemblages. Journal of Plant Ecology 5, 3–21.

Crepet, W.L., 2000. Progress in understanding angiosperm history, success, and relationships: Darwin’s abominably “perplexing phenomenon”. Proceedings of the National Academy of Sciences of the United States of America 97, 12939–12941.

Cripps, M.G., Schwarzländer, M., McKenney, J.L., Hinz, H.L., Price, W.J., 2006. Biogeographical comparison of the arthropod herbivore communities associated with *Lepidium* *draba* in its native, expanded and introduced ranges. Journal of Biogeography 33, 2107–2119.

Currano, E.D, Wilf, P., Wing, S.L., Labandeira, C.C., Lovelock, E.C., Royer, D.L., 2008. Sharply increased insect herbivory during the Paleocene–Eocene Thermal Maximum. Proceedings of the National Academy of Sciences of the United States of America 105, 1960–1964.

Daghlian, C.P., 1978. A new melioloid fungus from the early Eocene of Texas. Palaeontology 21, 171–176.

Dailey, P.J., Graves, R.C., Kingsolver, J.M., 1978. Survey of Coleoptera collected on the common milkweed, *Asclepias* *syriaca*, at one site in Ohio. Coleopterists’ Bulletin 32, 223–229.

Danforth, B.N., Poinar, G.O. Jr., 2011. Morphology, classification, and antiquity of *Melittosphex* *burmensis* (Apoidea: Melittosphecidae) and implications for early bee evolution. Journal of Paleontology 85, 882–891.

Dick, M., 1982. Leaf-inhabiting fungi of eucalypts in New Zealand. New Zealand Journal of Forestry Science 12, 525–537.

Dilcher, D.L., 1963. Eocene epiphyllous fungi. Science 142, 667–669.

Dilcher, D.L., 1974. Approaches to the identification of angiosperm leaf remains. Botanical Review 40, 1–157.

Dilcher, D.L., 2000. Toward a new synthesis: Major evolutionary trends in the angiosperm fossil record. Proceedings of the National Academy of Sciences of the United States of America 97, 7030–7036.

Dilcher, D.L., Kovach, W.L. 1986. Early angiosperm reproduction: *Caloda* *delevoryana* gen. et sp. nov., a new fructification from the Dakota formation (Cenomanian) of Kansas. American Journal of Botany 73, 1230–1237.

Ding, Q.L., Labandeira, C.C., Ren, D., 2014. Biology of a leaf miner (Coleoptera) on *Liaoningocladus boii* (Coniferales) from the Early Cretaceous of northeastern China and the leaf-mining biology of possible insect culprit clades. Arthropod Systematics & Phylogeny 72, 281–308.

Ding. Q.L., Labandeira, C.C., Meng, Q.M., Ren, D., 2015. Insect herbivory, plant-host specialization and tissue partitioning on mid-Mesozoic broadleaved conifers of Northeastern China. Palaeogeography, Palaeoclimatology, Palaeoecology 440, 259–273.

Dmitriev, V.I., Zherikhin, V.V., 1988. Changes in the diversity of insect families from data of first and last occurrences. In: Ponomarenko, A.G. (Ed.), The Mesozoic–Cenozoic Crisis in the Evolution of Insects, 208–215. Nauka, Moscow (in Russian).

Dong. J., Sun, B., Mao, T., Yan, D., Liu, C., Wang, Z., Jin, P., 2018. *Liquidambar* (Altingiaceae) and associated insect herbivory from the Miocene of southeastern China. Palaeogeography, Palaeoclimatology, Palaeoecology 497, 11–24.

Donovan, M.P, Wilf, P., Labandeira, C.C., Johnson, K.R., Peppe, D.J., 2014. Novel insect leaf-mining after the end-Cretaceous extinction and the demise of Cretaceous leaf miners, Great Plains, USA. PloS One 9, e103542, <https://doi.org/10.1371/journal.pone.0103542>

Donovan, M.P., Iglesias, A., Wilf, P., Labandeira, C.C., Cúneo, N.R., 2016. Rapid recovery of plant-insect associations after the end Cretaceous extinction. Nature Ecology and Evolution 1, 0012, <https://doi.10.1038/s41559-016-0012>

Doorenweerd, C., Nieukerken, E.J. van, Sohn, J.C., Labandeira, C.C., 2015. A revised checklist of Nepticulidae fossils (Lepidoptera) indicates an Early Cretaceous origin. Zootaxa 3963, 295–334.

Dormann, C.F., Gruber, B., Fründ, J., 2008. Introducing the bipartite package: analyzing ecological networks. R News 8, 8–11, <https://benthamopen.com/contents/pdf/TOECOLJ/TOEcolJ-2-1-7.pdf> .

Dos Santos, T.B., Pinheiro, E.R.S., Iannuzzi, R., 2020. First evidence of seed predation by arthropods from Gondwana and its early Paleozoic history (Rio Bonito Formation, Paraná Basin, Brazil). Palaios 35, 292–301.

Dotzler, N., Taylor, T.N., Galtier, J., Krings, M. 2011. *Sphenophyllum* (Sphenophyllales) leaves colonized by fungi from the Upper Pennsylvanian Grand-Croix cherts of central France. Zitteliana 51, 3–8.

Doyle, J.A., 2012. Molecular and fossil evidence on the origin of angiosperms. Annual Review of Earth and Planetary Sciences 40, 301–326.

Edwards, P.J., Wratten, S.D., 1983. Wound induced defences in plants and their consequences for patterns of insect grazing. Oecologia 59, 88–93.

Ehrlich, P.R., Raven, P.H., 1964. Butterflies and plants: a study in coevolution. Evolution 18, 586–608.

Ellis, B., Daly, D.C., Hickey, L.J., Johnson, K.R., Wilf, P., Wing, S., 2009. Manual of leaf architecture. First edition. Comstock Publishing and Cornell University Press, Ithaca, New York.

Endara, M.J., Coley, P.D., 2011.The resource availability hypothesis revisited: a meta-analysis*.* Functional Ecology 25, 389–398.

Evans, A.M., McKenna, D.D., Bellamy, C., Farrell, B.D., 2015. Large-scale molecular phylogeny of metallic wood-boring beetles (Coleoptera: Buprestoidea) provides new insights into relationships and reveals multiple evolutionary origins of the larval leaf-mining habit. Systematic Entomology 40, 385–400.

Fagua, G., Condamine, F.L., Horak, M., Zwick, A. Sperling, F.A.H., 2017. Diversification shifts in leafroller moths linked to continental colonization and the rise of angiosperms. Cladistics 33, 449–466.

Feeny, P., 1976. Plant apparency and chemical defense. In: Wallace, J.W., Mansell, R.L. (Eds.), Biochemical Interaction between Plants and Insects, 1–40. Plenum, New York.

Feng, Z., Su, T., Yang, J.Y., Chen, Y.X., Wei, H.B., Dai, J., Guo, Y., Liu, J.R., Ding, J.H., 2014. Evidence for insect-mediated skeletonization on an extant fern family from the Upper Triassic of China. Geology 42, 407–410.

Feng, Z., Wang, J., Rößler, R., Ślipiński, A., Labandeira, C.C., 2017. Late Permian wood-borings reveal an intricate network of ecological relationships. Nature Communications 8, 556, <https://doi.10.1038/s41467-017-00696-0>

Field, T.S., Arens, N.C., Doyle, J.A., Dawson, T.E., Donoghue, M.J., 2004. Dark and disturbed: a new image of early angiosperm ecology. Paleobiology 30, 82–107.

Filho, E.B.D.S., Adami-Rodrigues, K., Lima, F.J.D., Bantim, R.A.M., Wappler, T., Saraiva, A.A.F., 2017. Evidence of plant–insect interaction in the Early Cretaceous flora from the Crato Formation, Araripe Basin, Northeast Brazil. Historical Biology 31, 926–937.

Filip, V., Dirzo, R., Maass, J.M., Sarukhan, J., 1995. Within- and among-year variation in the levels of herbivory on the foliage of trees from a Mexican tropical deciduous forest. Biotropica 27, 78–86.

Formiga, A.T., Soares, G.L.G., Isais, R.M.S., 2011. Responses of the host plant tissues to gall induction in *Aspidosperma* *spruceanum* Müell. Arg. (Apocynaceae). American Journal of Plant Science 2, 823–834.

Frame, D., 2003. Generalist flowers, biodiversity and florivory: implications for angiosperm origins. Taxon 52, 681–685.

Futuyma, D.J., Keese, M.C., 1992. Evolution and coevolution of plants and phytophagous arthropods. In: Rosenthal, G.A., Berenbaum, M.R. (Eds.), Herbivores: Their Interactions with Secondary Plant Metabolites. Second edition, volume II, 439–475. Academic Press, San Diego.

Futuyma, D.J., Mitter, C., 1996. Insect–plant interactions: the evolution of component communities. Philosophical Transactions of the Royal Society B 351, 1361–1366.

Gangwere, S.K., 1966. Relationships between the mandibles, feeding behavior, and damage inflicted on plants by the feeding of certain acridids (Orthoptera). Great Lakes Entomologist 1, 13–16.

García-Massini, J., Channing, A., Guido, D.M., Zamuner, A.B., 2012. First report of fungi and fungus-like organisms from Mesozoic hot springs. Palaios 27, 55–62.

Gaston, K.J., Lawton, J.H., 1988. Patterns in body size, population dynamics and regional distribution of bracken herbivores. American Naturalist 132, 662–680.

Gaston, K.J., Lawton, J.H., 1989. Insect herbivores on bracken do not support the core-satellite hypothesis. American Naturalist 134, 761–777,

Gómez-Zurita J., Hunt, T., Kopliku, F., Vogler, A.P., 2007. Recalibrated tree of leaf beetles (Chrysomelidae) indicates independent diversification of angiosperms and their insect herbivores. PloS One 2, e360, <https://doi.10.1371/journal.pone.0000360>

Grez, A.A., González, R.H., 1995. Resource concentration hypothesis: effect of host-plant patch size on density of herbivorous insects. Oecologia 193, 471–474.

Gnaedinger, S., Adami-Rodrigues, A., Gallego, O.F., 2014. Endophytic oviposition on leaves from the Late Triassic of northern Chile: Ichnotaxonomic, palaeobiogeographic and palaeoenvironment considerations. Geobios 47, 221–236.

Grimaldi, D.A., 1999. The co-radiations of pollinating insects and angiosperms in the Cretaceous. Annals of the Missouri Botanical Garden 86, 373–406.

Grimaldi, D.A., Peñalver, E., Barrón, E., Herhold, H.K.W., Engel, M.S., 2019. Direct evidence for eudicot pollen-feeding in a Cretaceous stinging wasp (Angiospermae; Hymenoptera, Aculeata) preserved in Burmese amber. Communications Biology 2, 408, <https://doi.org/10.1038/s42003-019-0652-7>

Grubb, P.J., Jackson, R.V., Barberis, I.M., Bee, J.N., Coomes, D.A., Dominy, N.J., De La Fuente, M.A.S., Lucas, P.W., Metcalfe, D.J., Svenning, J.C., Turner, I.M., Vargas, O., 2008. Monocot leaves are eaten less than dicot leaves in tropical lowland rain forests correlations with toughness and leaf presentation. Annals of Botany 101, 1379–1389.

Gunkel, S., Wappler, T., 2015. Plant–insect interactions in the upper Oligocene of Enspel (Westerwald, Germany), including an extended mathematical framework for rarefaction. Palaeobiodiversity and Palaeoenvironments 95, 55–75.

Hamilton, R.W., 1980. Notes on the biology of *Eugnamptus collaris* (Fabr.) (Coleoptera: Rhynchitidae), with descriptions of the larva and pupa. The Coleopterists Bulletin 34, 227–236.

Hamilton, J.G., Zangerl, A.R., Berenbaum, M.R., Pippen, J., Aldea, M., DeLucia, E.H., 2004. Insect herbivory in an intact forest understory under experimental CO_2_ enrichment. Oecologia 138, 566–573.

Hanski, I., 1989. Fungivory: Fungi, insects and ecology. In: Wilding, N., Collins, N.M., Hammond, P.M., Webber, J.F. (Eds.), Insect–Fungus Interactions, 25–68. Academic Press, San Diego.

Hardy, N.B, Gullan, P.J., 2010. Australian gall-inducing scale insects on *Eucalyptus*: revision of *Opisthoscelis* Schrader (Coccoidea, Eriococcidae) and descriptions of a new genus and nine new species. ZooKeys 58, 1–24.

Harper, C.J., Taylor, T.N., Krings, M., Taylor, E.L., 2016. Structurally preserved fungi from Antarctica: diversity and interactions in late Palaeozoic and Mesozoic polar forest ecosystems. *Antarctic* Science 28, 153–173.

Hartkopf-Fröder C., Rust, J., Wappler, T., Friis, E.M., Viehofen, A., 2011. Mid-Cretaceous charred fossil flowers reveal direct observation of arthropod feeding strategies. Biology Letters 8, 295–298, <https://doi.10.1098/rsbl.2011.0696>

Hellmund, M., Hellmund, W., 2002. Neufunde und Ergänzungen zur Fortpflanzungsbiologie fossiler Kleinlibellen (Insecta, Odonata, Zygoptera). Stuttgarter Beiträge zu Naturkunde B 319, 1–26.

Herms, D.A., Mattson, W.J., 1992. The dilemma of plants: to grow or to defend. Quarterly Review of Biology 67, 283–335.

Hochwender, C.G., Fritz, R.S., 2004. Plant genetic differences influence herbivore community structure: evidence from a hybrid willow system. Oecologia 138, 547–557.

Hodkinson, I.D., 1985. Coevolution between psyllids (Homoptera: Psylloidea) and rain-forest trees: the first 120 million years. In: Chadwick, A.C., and Sutton, S.L. (Eds.), The Tropical Rain Forest: The Leeds Symposium, pp. 187–194. Leeds Philosophical Society, Leeds, United Kingdom.

Holyoak, M., Lawton, J.H., 1992. Detection of density dependence from annual censuses of bracken-feeding insects. Oecologia 91, 425–430.

Hsieh, T.C., Ma, K.H., Chao, A., 2016. iNEXT: An R package for rarefaction and extrapolation of species diversity (Hill numbers). Methods in Ecology and Evolution 7, 1451–1456.

Iannuzzi, R., Labandeira, C.C., 2008. The oldest record of external foliage feeding and the expansion of insect folivory on land. Annals of the Entomological Society of America 101, 79–94.

Imada, Y., Oyama, N., Shinoda, K., Takahashi, H., Yukawa, H., 2022. Oldest leaf mine trace fossil from East Asia provides insight into ancient nutritional flow in a plant-herbivore interaction. Scientific Reports 12, 1–12.

Imura, O., 2003. Herbivorous arthropod community of an alien weed *Solanum* *carolinense* L. Applied Entomology and Zoology 38, 293–300.

Isaka, Y., Sato, Y., 2015. Was species diversification in Tenthredinoidea (Hymenoptera: Symphyta) related to the origin and diversification of angiosperms? Canadian Entomologist 147, 443–458.

Jarzembowski, E.A., Ross, A.J., 1993. Time flies: the geological record of insects. Geology Today 9, 218–223.

Jarzembowski, E.A., Ross, A.J., 1996. Insect origination and extinction in the Phanerozoic. In: Hart, M.B. (Ed.), Biotic Recovery from Mass Extinction Events. Geological Society Special Publication 102, 65–78. Geological Society of London.

Jud, N.A., 2014. Morphotype catalog of a Zone 1 (Aptian–Earliest Albian) flora from Fairlington, Virginia, USA. Bulletin of the Peabody Museum of Natural History 55, 135–152.

Jud, N.A., Sohn, J.C., 2016. Evidence for an ancient association between leaf mining flies and herbaceous eudicot angiosperms. Cretaceous Research 63, 113–123.

Keane, R.M., Crawley, M.J., 2002. Exotic plant invasions and the enemy release hypothesis. Trends in Ecology & Evolution 17, 164–170.

Keathley, C.P., Potter, D.A., 2011. Behavioral plasticity of a grass-feeding caterpillar in response to spiny- or smooth-edged leaf blades. Arthropod–Plant Interactions 5, 339–349.

Kergoat, G.J., Bouchard, P., Clamens, A.L., Abbate, J.L., Jourdan, H., Jabbour-Zahab, R., Genson, G., Soldati, L., Condamine, F.L., 2014. Cretaceous environmental changes led to high extinction rates in a hyperdiverse beetle family. BMC Evolutionary Biology 14, 220, <http://www.biomedcentral.com/1471-2148/14/220>

Klymiuk, A.A., Stockey, R.A., Rothwell, G.A., 2015. Plant–arthropod interactions in *Acanthostrobus* *edensis* (Cupressaceae), a new conifer from the Upper Cretaceous of Vancouver Island, British Columbia. International Journal of Plant Sciences 176, 378–392.

Knor, S., Skuhravá, M., Wappler, T., Prokop, J., 2013. Galls and gall makers on plant leaves from the lower Miocene (Burdigalian) of the Czech Republic: Systematic and palaeoecological implications. Review of Palaeobotany and Palynology 188, 38–51.

Koeniger, G., Koeniger, N., O’Bryne, P., Lamb, A., Phiancharoen, M., Lim, H., Tihoi, S., Bosuang, S., 2017. Scientific note on *Apis* *koschevnikovi* chewing petals of an orchid flower (*Dipodium* species O’Byrne KIP1272). Apidiologie 48, 259–261.

Kohring, R. 1995. Fossile Bakterien und Pilzsporen aus dem Baltischen Bernstein. Neues Jahrbuch für Geologie und Paläontologie Monatshafte 6, 321–335.

Krantz, G.W., Lindquist, E.E., 1979. Evolution of phytophagous mites. Annual Review of Entomology 24, 121–158.

Krassilov, V.A., 2007. Mines and galls on fossil leaves from the Late Cretaceous of southern Negev, Israel. African Invertebrates 48, 13–22.

Krassilov, V.A., 2008a. Mine and gall predation as top down regulation in the plant–insect systems from the Cretaceous of Negev, Israel. Palaeogeography, Palaeoclimatology, Palaeoecology 261, 261–269.

Krassilov, V.A., 2008b. Evidence of temporary mining in the Cretaceous fossil mine assemblage of Negev, Israel. Insect Science 15, 285–290.

Krassilov, V.A., Bacchia, F., 2000. Cenomanian florule from Nammoura, Lebanon. Cretaceous Research 21,785–799.

Krassilov, V.A., Karasev, E., 2008. First evidence of plant–arthropod interaction at the Permian–Triassic boundary in the Volga Basin, European Russia. Alavesia 2, 247–252.

Krassilov, V.A., Rasnitsyn, A. (Eds.). 2008 Plant–arthropod interactions in the early angiosperm history: Evidence from the Cretaceous of Israel. Pensoft, Sofia and Moscow.

Krassilov, V.A., Shuklina, S., 2008. Arthropod trace diversity on fossil leaves from the mid-Cretaceous of Negev, Israel. Alavesia 2, 239–245.

Krassilov, V.A., Lewy, Z., Nevo, E., 2004. Controversial fruit-like remains from the Lower Cretaceous of the Middle East. Cretaceous Research 25, 697–707.

Krassilov, V.A., Silantieva, N., Hellmund, M., Hellmund, W., 2007. Insect egg sets on angiosperm leaves from the Lower Cretaceous of Negev, Israel. Cretaceous Research 28, 803–811.

Krings, M., Taylor, T.N., Dotzler, N., 2013. Fossil evidence of the zygomycetous fungi. Persoonia 30, 1–10.

Krings, M., Taylor, T.N., Taylor, E.L., Dotzler, N., Walker C., 2011. Arbuscular mycorrhizal-like fungi in Carboniferous arborescent lycopsids. New Phytologist 191, 311–314.

Laaß, M., Hoff, C., 2015. The earliest evidence of damselfly-like endophytic oviposition in the fossil record. Lethaia 48, 115–124.

Labandeira, C.C., 1994. A compendium of fossil insect families. Milwaukee Public Museum Contributions to Biology and Geology 88, 1–71.

Labandeira, C.C., 1998. Plant–insect associations from the fossil record. Geotimes 43, 18–24.

Labandeira, C.C., 2002. The history of associations between plants and animals. In: Herrera, C., Pellmyr, O. (Eds), Plant–Animal Interactions: An Evolutionary Approach. , 26–74, 248–261. Blackwell.

Labandeira, C.C., 2014. Why did terrestrial insect diversity not increase during the angiosperm radiation? Mid-Mesozoic, plant-associated insect lineages harbor some clues. In: Pontarotti, P. (Ed.), Evolutionary Biology: Genome Evolution, Speciation, Coevolution and Origin of Life, 261–299. Springer.

Labandeira, C.C., 2019. The fossil record of insect mouthparts: Innovation, functional convergence, and associations with other organisms. In: Krenn, H. (Ed.), Insect Mouthparts – Form, Function, Development and Performance. Zoological Monographs 5: Springer, 567–671.

Labandeira, C.C., 2021. Ecology and evolution of gall-inducing arthropods: The pattern from the terrestrial fossil record. Frontiers in Ecology & Evolution 9, 632449, <https://doi.org/10.3389/fevo/2021.632449>

Labandeira, C.C., Anderson, J.M., Anderson, H.M., 2018. Arthropod herbivory in Late Triassic South Africa: The Molteno Biota, the Aasvoëlberg 411 locality, and the developmental biology of a gall. In: Tanner, L. (Ed.), The Late Triassic World: Earth in a Time of Transition. Topics in Geobiology 46. Springer, 623–719.

Labandeira, C.C., Dilcher, D.R., Davis, D.R., Wagner, D.L., 1994. Ninety-seven million years of angiosperm-insect association: paleobiological insights into the meaning of coevolution. Proceedings of the National Academy of Sciences of the United States of America 91, 12278–12282.

Labandeira, C.C., Johnson, K.R., Wilf, P., 2002. Impact of the terminal Cretaceous event on plant–insect associations. Proceedings of the National Academy of Sciences of the United States of America 99, 2061–2066.

Labandeira, C.C., Kustatscher, E., Wappler, T., 2016. Floral assemblages and patterns of insect herbivory during the Permian to Triassic of Northeastern Italy. PloS One 11, 20161448, <https://doi.10.1098/rspb.2016.1448>

Labandeira, C.C., LePage, B.A., Johnson, A.H., 2001. A *Dendroctonus* bark engraving (Coleoptera: Scolytidae) from a middle Eocene *Larix* (Coniferales: Pinaceae): early or delayed colonization? American Journal of Botany 88, 2026–2039.

Labandeira, C.C., Phillips, T.L., 2002. Stem borings and petiole galls from Pennsylvanian tree ferns of Illinois, USA: Implications for the origin of the borer and galler functional-feeding-groups and holometabolous insects. Palaeontographica Abteilung A 264: 1–84, pls. 1–16.

Labandeira, C.C., Prevec, R., 2014. Plant paleopathology and the roles of pathogens and insects. International Journal of Paleopathology 4, 1–16.

Labandeira, C.C., Sepkoski, J.J. Jr., 1993. Insect diversity in the fossil record. Science 261, 310–315.

Labandeira, C.C., Tremblay, S.L., Bartowski, K.E., VanAller Hernick, L., 2014. Middle Devonian liverwort herbivory and antiherbivore defence. New Phytologist 202, 247–258.

Labandeira, C.C., Wilf, P., Johnson, K.R., Marsh, F., 2007. Guide to insect (and other) damage types on compressed plant fossils (version 3.01). Smithsonian Institution, National Museum of Natural History, Department of Paleobiology, Washington, DC.

Larew, H.G., 1981. A comparative anatomical study of galls caused by the major cecidogenetic groups, with special emphasis on the nutritive tissue (Unpubl. Ph.D. thesis), Oregon State University, Corvallis, pp.1–392.

Lawton, J.H., 1976. The structure of the arthropod community on bracken. Botanical Journal of the Linnean Society 73, 187–216.

Lawton, J.H., 1978. Host-plant influences on insect diversity: the effects of space and time. In: Mound, L.A., Waloff, N. (Eds.), Symposium of the Royal Entomological Society 9, 105–125. Blackwell Scientific Publications, Oxford, United Kingdom.

Lawton, J.H., 1982. Vacant niches and unsaturated communities: A comparison of bracken herbivores at sites on two continents. Journal of Animal Ecology 51, 573–595.

Lawton, J.H., 1984. Non-competitive populations, non-convergent communities, and vacant niches: the herbivores of bracken. In: Strong, D.R., Simberloff, D., Abele, L.G., Thistle, A.B., (Eds.), Ecological Communities: Conceptual Issues and the Evidence, 67–101. Princeton University Press, Princeton, New Jersey.

Lawton, J.H., Gaston, K.J., 1989. Temporal patterns in the herbivorous insects of bracken: A test of community predictability. Journal of Animal Ecology 58, 1021–1034.

Leavitt, H., Robinson, I.C., 2006. Petal herbivory by chrysomelid beetles (*Phyllotreta* sp.) is detrimental to pollination and seed production in *Lepidium* *papilliferum* (Brassicaceae). Ecological Entomology 31, 657–660.

Leppanen, S.A., Altenkhofer, E., Liston, A.D., Nyman, T., 2012. Phylogenetics and evolution of a host-plant use in leaf-mining sawflies (Hymenoptera: Tenthredinidae: Heterarthrinae). Molecular Phylogenetics and Evolution 64, 331–341.

Lewinsohn, T.M., Novotny, V., Basset, Y., 2005. Insects on plants: Diversity of herbivore assemblages revisited. Annual Review of Ecology and Evolution 36, 597–620.

Lin, X.D., Labandeira, C.C., Ding, Q.L., Meng, Q.M., Ren, D., 2019. Exploiting nondietary resources in deep time: patterns of oviposition on mid-Mesozoic plants from northeastern China, International Journal of Plant Sciences 180, 411–457.

Liu, J.K., Hyde, K.D., Jones, E.B.G., Ariyawansa, H.A., Bhat, D.J., Boonmee, S., Maharachchikumbura, S.S.N., McKenzie, E.H.C., et al. 2015, Fungal diversity notes 1–110: taxonomic and phylogenetic contributions to fungal species*.* Fungal Diversity, 72, 1–197, <http://doi.10.1007/s13225-015-0324-y>

Loomis, W.E., 1953. Growth and differentiation—an introduction and summary. In: Loomis, W.E., (Ed.), Growth and Differentiation in Plants, 1–17. Iowa State College Press, Ames, Iowa.

Lopez-Vaamonde, C., Wikström, N., Labandeira, C.C., Goodman, S., Godfray, H.C.J., Cook, J.M., 2006. Fossil-calibrated molecular phylogenies reveal that leaf-mining moths radiated millions of years after their host plants. Journal of Evolutionary Biology 19, 1314–1326.

Lowman, M.D., 1987. Relationships between leaf growth and holes caused by herbivores. Australian Journal of Ecology 12, 189–191.

Lutzoni, F., Nowak, M.D., Alfaro, M.E., Reeb, V., Miadlikowska, J., Krug, M., Arnold, E., Lewis, L.A., et al., 2018. Contemporaneous radiations of fungi and plants linked to symbiosis*.* Nature Communications 9, 5451, <http://doi.org/10.1038/s41467-018-07849-9>

Maccracken, S.A., Labandeira, C.C., 2020. The middle Permian South Ash Pasture assemblage of north-central Texas: Coniferophyte and gigantopterid herbivory and longer-term herbivory trends. International Journal of Plant Sciences 181, 342–362.

Maccracken, S.A., Miller, I.M., Johnson, K.R., Sertich, J.J.W., Labandeira, C.C., 2021. Insect herbivory on *Catula* *gettyi* gen. et sp. nov. (Lauraceae) from the Kaiparowits Formation (Late Cretaceous, Utah, USA). PloS One, in press.

Magallón, S., Castillo, A., 2009. Angiosperm diversification through time. American Journal of Botany 96, 349–365.

Manchester, S.R., Dilcher, D.L., Judd, W.S., Brandon, C., Basinger, J.F., 2018. Early eudicot flower and fruit: *Dakotanthus* gen. nov. from the Cretaceous Dakota Formation of Kansas and Nebraska, USA. Acta Palaeobotanica 58, 27–40, <https://doi.10.2478/acpa-2018-0006>

Martin, N.A., 2017. Hadda beetle – *Epilachna* *vigintioctopunctata* – interesting insects and other invertebrates. New Zealand Arthropod Factsheet Series 28, <http://nzafactsheets.lanndcareresearch.co.nz/ndex.html>

Maslova, N.P., Vasilenko, D.V., Kodrul, T.K., 2016. Phytopathology in fossil plants: New data, questions of classification. Paleontological Journal 50, 202–208.

McCall, A.C., Irwin, R.E., 2006. Florivory: the intersection of pollination and herbivory. Ecology Letters 9, 1351–1365.

McKenna, D.G., Sequeira, A.S., Marvaldi, A.E., Farrell, B.D., 2009. Temporal lags and overlap in the diversification of weevils and flowering plants. Proceedings of the National Academy of Sciences of the United States of America 106, 7083–7088.

McLoughlin, S., 2011. New records of leaf galls and arthropod oviposition scars in Permian–Triassic Gondwanan gymnosperms. Australian Journal of Botany 59, 156–169.

McLoughlin, S., Martin, S.K., Beattie, R., 2015. The record of Australian Jurassic plant–arthropod interactions. Gondwana Research 27, 940–959.

Meineke, E.K, Davies, T.J., 2018. Museum specimens provide novel insights into changing plant–herbivore interactions. Philosophical Transactions of the Royal Society B 374, 20170393, <https://doi.org/10.1098/rstb.2017.0393>

Meng, Q.M., Labandeira, C.C., Ding, Q.L., Ren, D., 2017. The natural history of oviposition on a ginkgophyte fruit from the Middle Jurassic of northeastern China. Insect Science 26, 171–179.

Mikulás, R., Dvorak, Z., Pek, I., 1998. *Lamniporichnus* *vulgaris* igen. et isp. nov.: traces of insect larvae in stone fruits of hackberry (*Celtis*) from the Miocene and Pleistocene of the Czech Republic. Journal of the Czech Geological Society 43, 277–280.

Mithöfer, A., Wanner, G., Boland, W., 2005. Effects of feeding *Spodoptera* *littoralis* on lima beans. II. Continuous mechanical wounding resembling insect feeding is sufficient to elicit herbivory-related volatile emission. Plant Physiology 137, 1160–1168, <https://doi.org/10.1104/pp.104.054460>

Na, Y.L., Sun, C.L., Li, T., Li, Y.F., 2014. The insect oviposition firstly discovered on the Middle Jurassic Ginkgoales leaf from Inner Mongolia, China. Acta Geol Sinica 88: 18–28.

Na, Y.L., Sun, C.L., Wang, H.S., Dilcher, D.L., Yang, Z.Y., Li, T., Li, Y.F., 2018. Insect herbivory and plant defense on ginkgoalean and bennettitalean leaves of the Middle Jurassic Daohugou Flora from Northeast China and their paleoclimatic implications. Palaeoworld 27, 202–210.

Naugolnykh, S.V., Ponomarenko, A.G., 2010. Possible traces of feeding by beetles in coniferophyte wood from the Kazanian of the Kama River Basin. Paleontological Journal 44, 468–474.

Nieukerken, E.J. van, Doorenweerd, C., Nishida, K., Snyers, C., 2016. New taxa, including three new genera showing uniqueness of Neotropical Nepticulidae (Lepidoptera). ZooKeys 628, 1–63.

Nordman, E.E., Robison, D.J., Abrahamson, L.P., Volk, T.A., 2005. Relative resistance of willow and poplar biomass production clones across a continuum of herbivorous insect specialization: Univariate and multivariate approaches. Forest Ecology and Management 217, 307–318.

Nyman, T., Widmer, A., Roininen, H., 2000. Evolution of gall morphology and host-plant relationships in willow-feeding sawflies (Hymenoptera: Tenthredinidae). Evolution 54, 526–533.

Orians, C.M., Huang, C.H., Wild, A., Dorfman, K.A., Zee, P., Dao, M.T.T., Fritz, R.S., 1997. Willow hybridization differentially affects preference and performance of herbivorous beetles. Entomologia Experimentalis et Applicata 83, 285–294.

Peppe, D.J., Hickey, L.J., Miller, I.M., Green, W.A., 2008. A morphotype catalogue, floristic analysis and stratigraphic description of the Aspen Shale Flora (Cretaceous–Albian) of southwestern Wyoming. Bulletin of the Peabody Museum of Natural History 49, 181–208.

Percy, D.M., Page, R.D.M., Cronk, Q.C.B., 2004. Plant–insect interactions: double-dating associated insect and plant lineages reveals asynchronous radiations. Systematic Biology 53, 120–127.

Peris, D.R., Labandeira, C.C., Barrón, E., Delclòs, X., Rust, J., Wang, B., 2020. Generalist pollen-feeding beetles during the mid-Cretaceous. iScience 23, 100913, <https://doi.org/10.1016/j.sci.2020.100913>

Pienkowski, R.L., Kok, L.T., 1976. Major phytophagous insects of selected weeds in Virginia. In: Freeman, T.E. (Ed.), Proceedings of the Fourth International Symposium of the Biological Control of Weeds, 137–140. University of Florida, Gainesville.

Pires, E.F., Sommer, M.G., 2009. Plant–arthropod interaction in the Early Cretaceous (Berriasian) of the Araripe Basin, Brasil. Journal of South American Earth Sciences 27, 50–59.

Pollard, D.G., 1968. Directional control of the stylets in phytophagous Hemiptera. Proceedings of the Royal Entomological Society of London A (General Entomology) 44, 173–185.

Prevec, R., Labandeira, C.C., Neveling, J. Gastaldo, R. A., Looy, C., Bamford, M.A., 2009. A portrait of a Gondwanan ecosystem: a new Late Permian locality from KwaZulu–Natal, South Africa. Review of Palaeobotany and Palynology 156, 454–493.

Price, P.W., Wilson, M.F., 1979. Abundance of herbivores on six milkweed species in Illinois. American Midland Naturalist 101, 76–86.

Radwanski, A., 1977. Present-day types of traces in the Neogene sequence: their problems of nomenclature and preservation. In: Crimes, T.P., Harper, J.C., Jr. (Eds.), Trace Fossils, 2, 227–264. Seel House, Liverpool.

Rajchel, J., Uchman, A., 1998. Insect borings in Oligocene wood, Kliwa Sandstones, Outer Carpathians, Poland. Annales Societatis Geologorum Polonica 68, 219–224.

Rasnitsyn, A.P., 1988. Principles and methods of phylogenetic reconstruction. In: Ponomarenko, A.G. (Ed.), The Mesozoic–Cenozoic Crisis in the Evolution of Insects, 191–207. Nauka, Moscow (in Russian).

Rhodes, D.F., Cates, R.G., 1976.Toward a general theory of plant antiherbivore chemistry. In: Wallace, J.W., Mansell, R.I. (Eds.), Recent Advances in Phytochemistry 10, 168–213. Plenum, New York.

Rigby, C., Lawton, J.H., 1981. Species-area relationships of arthropods on host plants: Herbivores on bracken. Journal of Biogeography 8, 125–133.

Rikkinen, J, Poinar, G.O. Jr., 2000. A new species of resinicolous *Chaenothecopsis* (Mycocaliciaceae, Ascomycota) from 20 million-year-old Bitterfeld amber, with remarks on the biology of resinicolous fungi. Mycological Research 104, 7–15.

Roche, B.M, Fritz, R.S., 1997. Genetics of resistance of *Salix* *serica* to a diverse community of herbivores. Evolution 51, 1490–1498.

Root, R.B., 1973. Organization of a plant–arthropod association in simple and diverse habitats: the fauna of collards (*Brassica* *oleracea*). Ecological Monographs 43, 95–124.

Royer, D.L., Miller, I.M., Peppe, D.J., Hickey, L.J., 2010. Leaf economic traits from fossils support a weedy habit for early angiosperms. American Journal of Botany 97, 438–445.

Rozefelds, A.C., 1988. Lepidoptera mines in *Pachypteris* leaves (Corystospermaceae: Pteridospermophyta) from the Upper Jurassic/Lower Cretaceous Battle Camp Formation, North Queensland. Proceedings of the Royal Society of Queensland 99, 77–81.

Santos, A.A., Sender, L.M., Wappler, T., Engel, M.S., Diez, J.B., 2021. A Robinson Crusoe story in the fossil record: Plant-insect interactions from a Middle Jurassic ephemeral volcanic island (Eastern Spain). Palaeogeography, Palaeoclimatology, Palaeoecology 583, 110655.

Sarzetti, L.C., Labandeira, C.C., Muzón, J., Wilf, P., Cúneo, N.R., Johnson, K.R., Genise, J.F., 2009. Odonatan endophytic oviposition from the Eocene of Patagonia: The ichnogenus *Paleoovoidus* and implications for behavioral stasis. Journal of Paleontology 83, 431–447.

Schachat, S.R., Labandeira, C.C., 2015. Evolution of a complex behavior: the origin and initial diversification of foliar galling by Permian insects. The Science of Nature 102, 14, <https://doi.10.1007/s00114-015-1266-7>

Schachat, S.R., Labandeira, C.C., Gordon, J., Chaney, D.S., Levi, S., Halthore, M.S., Alvarez, J., 2014. Plant–insect interactions from the Early Permian (Kungurian) Colwell Creek Pond, North-Central Texas: the early spread of herbivory in riparian environments. International Journal of Plant Sciences 175, 855–890.

Schachat, S.R., Labandeira, C.C., Chaney, D.S., 2015. Insect herbivory from early Permian Mitchell Creek Flats of north-central Texas: Opportunism in a balanced component community. Palaeogeography, Palaeoclimatology, Palaeoecology 440, 830–847.

Schachat, S.R., Labandeira, C.C., Clapham, M.E., Payne, J.L., 2019. A Cretaceous peak in family-level insect diversity estimated with mark–recapture methodology. Proceedings of the Royal Society B (Biological Science) 286, 20192054. <https://doi.org/10.1098/rspb.2019.2054>

Schmidt, A.R., Dörfelt, H., Struwe, S., Perrichot, V. 2010. Evidence for fungivory in Cretaceous amber forests from Gondwana and Laurasia. Palaeontographica Abteilung B 283, 157–173.

Scott, A.C., Stephenson, J., Collinson, M.E., 1994, The fossil record of leaves with galls. In: Williams, M.A.J. (Ed.), Plant Galls: Organisms, Interactions, Populations. Systematics Association Special Volume 49, 447–470. Clarendon Press, Oxford, United Kingdom.

Scott, A.C., Taylor, T.N., 1983. Plant/animal interactions during the Upper Carboniferous. Botanical Review 49, 259–307.

Shackel, K.A., Polito, V.S., Ahmadi, H., 1991. Maintenance of turgor by rapid sealing of puncture wounds in leaf epidermal cells. Plant Physiology 97, 907–912.

Sharov, A.G., 1973. Morphological features and mode of life of the Palaeodictyoptera. In: Bei-Benko, G.Y. (Ed.), Readings in the Memory of Nicolaj Aleksandrovich Kholodovskij, 49–63. Nauka, Leningrad (in Russian).

Shcherbakov, D.E., 2008. Madygen, Triassic Lagerstätte number one, before and after Sharov. Alavesia 2, 113–124.

Shcherbakov, D.E., Makarkin, V.N., Aristov, D.S., Vasilenko, D.V., 2009. Permian insects from the Russky Island, South Primorye. Russian Entomological Journal 18, 7–16.

Shcherbakov, D.E., 2011. New and little-known families of Hemiptera Cicadomorpha from the Triassic of Central Asia―early analogs of treehoppers and planthoppers. Zootaxa 2836, 1–26.

Sheffy, M.V., Dilcher, D.L., 1971. Morphology and taxonomy of fungal spores. Palaeontographica Abteilung B 133, 34–51, pls. 13–16.

Shi, G.L., Zhou, Z.Y., Xie, Z.M., 2010. A new *Cephalotaxus* and associated epiphyllous fungi from the Oligocene of Guangxi, South China. Review of Palaeobotany and Palynology 161, 179–195, <http://doi.10.1016/j.revpalbo.2010.04.002>

Slater, B.J., McLoughlin, S., Hilton, J., 2012. Animal–plant interactions in a Middle Permian permineralised peat of the Bainmedart Coal Measures, Prince Charles Mountains, Antarctica. Palaeogeography, Palaeoclimatology, Palaeoecology 363–364, 109–126.

Slater, B.J., McLoughlin, S., Hilton, J., 2013. Peronosporomycetes (Oomycota) from a middle Permian permineralised peat within the Bainmedart Coal Measures, Prince Charles Mountains, Antarctica. PLoS One, 8, 70707, <https://doi.10.1371/journal.pone.0070707>

Smith, D.M., Nufio, C.R., 2003. Levels of herbivory in two Costa Rican rain forests: Implications for studies of fossil herbivory. Biotropica 36, 318–326.

Sohn, J.C., Kim, N.H., Choi, S.W., 2017. Morphological and functional diversity of foliar damage on *Quercus* *mongolica* Fisch. ex Ledeb. (Fagaceae) by herbivorous insects and pathogenic fungi. Journal of Asia-Pacific Biodiversity 10, 489–508.

Soltis, P.S., Soltis, D.E., 2004. The origin and diversification of angiosperms. American Journal of Botany 91, 1614–1626.

Song, H., Amédégnato C., Cigliano, M.M., Desutter-Grandcolas, L., Heads, S.W., Huang, D.Y., Otte, D., Whiting, M.F., 2015. 300 million years of diversification: elucidating the patterns of orthopteran evolution based on comprehensive taxon and gene sampling. Cladistics 31, 621–651.

Song, N., Liang, A.P., 2013. A preliminary molecular phylogeny of planthoppers (Hemiptera: Fulgoridea) based on nuclear and mitochondrial DNA sequences. PloS One 8, e58400, <http://doi.10.1371/journal.pone.0058400>

Srivastava, A.K. 1993. Evidence of fungal parasitism in the Glossopteris Flora of India. Comptes Rendus of the Twelfth International Committee for Coal and Organic Petrology 2, 141–146. International Congress of Carboniferous and Permian, Buenos Aires.

Stamp, N., 2003. Out of the quagmire of plant defense hypotheses. The Quarterly Review of Biology 78, 23–55.

Stireman, III, J.O., Devlin, H., Carr, T.G., Abbot, P., 2010. Evolutionary diversification of the gall midge genus *Asteromyia* (Cecidomyiidae) in a multitrophic ecological context. Molecular Phylogenetics and Evolution 54, 194–210.

Stull, G.W., Labandeira, C.C., DiMichele, W.A., Chaney, D.S., 2013. The “seeds” on *Padgettia readi* are insect galls: reassignment of the plant to *Odontopteris*, the gall to *Ovofoligallites* ten. And the evolutionary implications thereof. Journal of Paleontology 87, 217–231.

Szwedo, J., 2002. Amber and amber inclusions of planthoppers, leafhoppers and their relatives (Hemiptera, Archaeorrhyncha et Clypaeorrhyncha). Denisia 176, 37–56.

Szwedo, J., 2004. *Niryasaburnia* gen. nov. for ‘*Liburnia*’ *burmitina* Cockerell, 1717, from Cretaceous Myanmar (Burmese) amber (Hemiptera, Fulgoromorpha: Achilidae). Journal of Systematic Palaeontology 2, 105–107.

Tapanila, L., Roberts, E. M., 2012. The earliest evidence of holometabolan insect pupation in conifer wood. Plos One 7, e31668.

Taylor, T.N., Osborn, J.M., 1996. The importance of fungi in shaping the paleoecosystem. Review of Palaeobotany and Palynology 90, 249–262.

Tian, N., Wang, Y., Zheng, S., Zhu, Z. 2020. White-rotting fungus with clamp-connections in a coniferous wood from the Lower Cretaceous of Heilongjiang Province, NE China. Cretaceous Research 105, 104014.

Tuomi, J., Fagerström, T., Niemelä, P., 1991. Carbon allocation, phenotypic plasticity, and induced defenses. In: Tallamy, D.W., Raupp, M.J. (Eds.), Phytochemical Induction by Herbivores, 85–104. Wiley, New York.

Upchurch, G.R., Dilcher, D.L., 1990. Cenomanian angiosperm leaf megafossils, Dakota Formation, Rose Creek locality, Jefferson County, southeastern Nebraska. United States Geological Survey Bulletin 1915, 1–55.

Urban, J., 2006a. Occurrence, bionomics and harmfulness of *Chrysomela* *populi* L. (Coleoptera, Chrysomelidae). Journal of Forest Science 52, 255–284.

Van Zandt, P.A., Agrawal, A.A., 2004. Community-wide impacts of herbivore-induced plant responses in milkweed (*Asclepias* *syriaca*). Ecology 85, 2616–2629.

Vasilenko, D.V., 2005. Damages on Mesozoic plants from the Transbaikalian locality Chernovskie Kopi. Paleontological Journal 39, 628–633.

Vasilenko, D.V., Maslova, N.P., Herman, A.B., 2016. Galls on the *Compositiphyllum* *retinerve* (Herman) Herman et Kvaček leaves (angiosperms) from the Turonian of the northwestern Kamchatka Peninsula, Russia. Paleontological Journal 50, 653–657.

Vea, I.M., Grimaldi, D.A., 2016. Putting scales into evolutionary time: the divergence of major scale insect lineages (Hemiptera) predates the radiation of modern angiosperm hosts. Science Reports 6, 23487, <http://doi.10.1038/srep23487>

Vincent, J.F.W., 1990. Fracture properties of plants. Advances in Botanical Research 19, 235–287.

Visscher, H., Sephton, M.A., Looy, C.V. 2011. Fungal virulence at the time of the end-Permian biosphere crisis? Geology 39, 883–886.

Wahlberg, N., Wheat, C.W., Peña, C., 2013. Timing and patterns in the taxonomic diversification of Lepidoptera (butterflies and moths). PloS One 8, e80875, <https://doi.org/10.1371/journal.pone.0080875>

Walker, J.D., Geissman, J.W., Bowring, S.A., Babcock, L.E., 2018. Geologic time scale v. 5.0. Geological Society of America Bulletin, doi: 10.1130/2018CTS005R3C

Walling, L.L., 2008. Avoiding effective defenses: Strategies employed by phloem-feeding insects. Plant Physiology 146, 859–866.

Wang, B., Zhang, H., Jarzembowski, E.A., 2013a. Early Cretaceous angiosperms and beetle evolution. Frontiers in Plant Science 4, 360, <http://doi.10.3389/fpls.2013.00360>

Wang, J., Labandeira, C.C., Zhang, Z.F., Bek, J., Pfefferkorn, H.W., 2009. Permian *Circulipuncturites* *discinisporis* Labandeira, Wang, Zhang, Bek et Pfefferkorn gen. et spec. nov. (formerly *Discinispora*) from China, an ichnotaxon of punch-and-sucking insect on Noeggeranthialean spores. Review of Palaeobotany and Palynology 156, 277–282.

Wappler, T., 2010. Insect herbivory close to the Oligocene-Miocene transition –A quantitative analysis. Palaeogeography, Palaeoclimatology, Palaeoecology 292, 540–550.

Wappler, T., Ben-Dov, Y., 2008. Preservation of armoured scale insects on angiosperm leaves from the Eocene of Germany. Acta Palaeontologica Polonica 53, 627–634.

Wappler, T., Currano, E.D., Wilf, P., Rust, J., Labandeira, C.C., 2009. No post-Cretaceous ecosystem depression in European forests? Rich insect-feeding damage on diverse middle Palaeocene plants, Menat, France. Proceedings of the Royal Society B (Biological Science) 276, 4271-4277.

Wappler, T., Labandeira, C.C., Rust, J., Frankenhäuser, H., Wilde, V., 2012. Testing for the effects and consequences of mid Paleogene climate change on insect herbivory. Plos One 7, e40744, <https://doi.org/10.1031/journal.pone.0040744>

Weiss, H.B., Dickerson, E.L., 1921. Notes on milkweed insects in New Jersey. Journal of the New York Entomological Society 29, 123–145.

Whittaker, J.B., 1984. Responses of sycamore (*Acer* *pseudoplatanus*) leaves to damage by a typhlocybine leaf hopper, *Ossiannilssonola* *callosa*. Journal of Ecology 72, 455–462.

Wikström, N., Savolainen, V., Chase, M.W., 2001. Evolution of the angiosperms: calibrating the family tree. Proceedings of the Royal Society of London B (Biological Sciences) 268, 2211–2220.

Wilf, P., and Labandeira, C.C., 1999. Response of plant–insect associations to Paleocene–Eocene warming. Science 284, 2153–2156.

Wilf, P., Labandeira, C.C., Johnson, K.R., Coley, P.D., Cutter, A.D., 2001. Insect herbivory, plant defense, and early Cenozoic climate change. Proceedings of the National Academy of Sciences of the United States of America 98, 6221–6226.

Wing, S.L., Herrera, F., Jaramillo, C., Gómez, C., Wilf, P., Labandeira, C.C., 2009. Late Paleocene fossils from the Cerrejón Formation, Colombia, are the earliest record of Neotropical rainforest. Proceedings of the National Academy of Sciences of the United States of America 106, 18627–18632.

Winkler, I.S., Labandeira, C.C., Wappler, T., Wilf, P., 2010. Diptera (Agromyzidae) leaf mines from the Paleogene of North America and Germany: Implications for host use evolution and an early origin for the Agromyzidae. Journal of Paleontology 84, 935–954.

Wise, M.J., 2007. Evolutionary ecology of resistance to herbivory: an investigation of potential genetic constraints in the multiple-herbivore community of *Solanum* *carolinense*. New Phytologist 175, 773–784.

Wolfe, J.A., 1973. Fossil forms of Amentiferae. Brittonia 25, 334–355.

Xiao, L.F., Labandeira, C.C., Dilcher, D., Ren, D., 2021a. Florivory of Early Cretaceous flowers by functionally diverse insects: implications for early angiosperm pollination. Proceedings of the Royal Society B (Biological Sciences) 288, 20210320. https://doi.org/10.1098/rspb.2021.0320.

Xiao, L.F., Labandeira, C.C., Ben-Dov, Y., Maccracken, S.A., Shih, C.K., Dilcher, D.L., Ren. D., 2021b. Early Cretaceous mealybug herbivory on a laurel highlights the deep-time history of angiosperm–scale insect associations. New Phytologist 232, 1414–1423. doi.org/10.1111/NPH.17672.

Xiao, L.F., Labandeira, C.C., Dilcher, D., Ren, D., 2021c. Arthropod and fungal herbivory at the dawn of angiosperm diversification: The Rose Creek plant assemblage of Nebraska, U.S.A. Cretaceous Research 131, 105088. <https://doi.org/10.1016/j.cretres.2021.105088>.

Xiao, L.F., Labandeira, C.C., and Ren, D., 2022. Insect herbivory immediately before the eclipse of the gymnosperms: The Dawangzhangzi plant assemblage of Northeastern China. Insect Science, 1–38. DOI 10.1111/1744-7917.12988.

Xu, Q, Jin, H., Labandeira, C.C., 2018. Williamson Drive: Herbivory on a north-central Texas flora of latest Pennsylvanian age shows discrete component community structure, early expansion of piercing and sucking, and plant counterdefenses. Review of Palaeobotany Palynology 251, 28–72.

Yao, Y., Ren, D., Rider, D.A., Cai, W., 2012. Phylogeny of the Infraorder Pentatomormorpha based on fossil and extant morphology, with description of a new fossil family from China. Plos One 7, e37289, <https://doi.10.1371/journal.pone.0037289>

Zeng, L., Zhang, Q., Sun, R., Kong, H., Zhang, N., Ma, H., 2014. Resolution of deep angiosperm phylogeny using conserved nuclear genes and estimates of early divergence times. Nature Communications 5, 4956, <https://doi.10.1038/ncomms5956>

Züst, T., Agrawal, A.A., 2016. Population growth and sequestration of plant toxins along a gradient of specialization in four aphid species on the common milkweed *Asclepias* *syriaca*. Functional Ecology 30, 547–556.
